# Targeted knockout of a host peroxisomal peptidase confers field resistance to maize lethal necrosis

**DOI:** 10.1073/pnas.2535202123

**Published:** 2026-04-30

**Authors:** Mark Jung, Zhengyu Wen, Sabrina Humbert, Fengzhong Lu, Alyssa DeLeon, Lisa Marshall, Craig Hastings, Heather Cartwright, Katherine Thilges, Ning Wang, Kassandra Breckenridge, Emily Wu, Larisa Ryan, Kevin Fengler, Kevin Simcox, Shawn Thatcher, Victor Llaca, Grace Woollums, Jeffry Sander, Deping Xu, Mary Beatty, Kent Brink, Maria Fedorova, Mark Jones, Erik Ohlson, L. M. Suresh, Yoseph Beyene, Michael Olsen, Veronica Ogugo, Amos Alakonya, Ann Murithi, Stephen Mugo, James Karanja, Prasanna Boddupalli, Kevin Pixley, Marc Albertsen, Todd Jones, Robert Meeley, Neal Gutterson, Barbara Mazur, Kanwarpal S. Dhugga

**Affiliations:** ^a^ Corteva Agriscience, Johnston, IA 50131; ^b^ International Center for Maize and Wheat Improvement, Texcoco 56237, Mexico; ^c^Corn, Soybean, and Wheat Quality Research Unit, United States Department of Agriculture-Agricultural Research Service, Wooster, OH 44691; ^d^ International Center for Maize and Wheat Improvement, Nairobi, Kenya 00100; ^e^ Kenyan Agricultural and Livestock Research Organization, Nairobi, Kenya 00200

**Keywords:** maize lethal necrosis, genome editing, peroxisome, susceptibility factor, food security

## Abstract

Maize lethal necrosis (MLN) is a severe disease caused by the combined infection of maize chlorotic mottle virus (MCMV) and a potyvirus, most often sugarcane mosaic virus (SCMV). This disease seriously threatens food security across sub-Saharan Africa (SSA). We investigated a major-effect quantitative trait locus for resistance on chromosome 6, named the *maize lethal necrosis susceptibility locus 1* (*qMLNS1*), derived from the Thai line KS23-6. Fine mapping and CRISPR-Cas9 editing of the candidate genes within the narrowed 105 kb interval revealed a peroxisomal *peptidase* as the underlying cause of susceptibility. Confocal microscopy confirmed the localization of the MLNS1 protein within peroxisomes. Targeted knockout of the *Mlns1* gene in the susceptible elite line CML536 from SSA conferred resistance comparable to KS23-6 in field trials conducted in Naivasha, Kenya. This knockout specifically blocked MCMV accumulation without affecting SCMV. The edited lines showed no yield penalty or agronomic defects under disease-free conditions. Our findings uncover a mechanistic link between a peroxisomal enzyme and viral susceptibility. They also establish a rapid, scalable gene editing strategy for incorporating MLN resistance into elite germplasm, offering a model for combating similar viral diseases in staple crops globally.

## Significance

Maize lethal necrosis (MLN) is a serious viral disease threatening food security in East Africa. We found a previously unknown mechanism by which the virus exploits a specific maize peroxisomal peptidase to form replication compartments. This peptidase constitutes a critical genetic vulnerability. Its elimination using CRISPR-Cas technology confers robust MLN resistance. The edited elite African maize lines remain agronomically identical to their unedited counterparts in the absence of the disease. This targeted strategy provides an efficient, accelerated route to protect crop yields against the MLN threat, thereby safeguarding the livelihoods of vulnerable smallholder farmers.

Food insecurity is a persistent concern in sub-Saharan Africa (SSA) where maize (*Zea mays* L.) is a staple crop and two-thirds of the population subsists on small farms (1). In Eastern and Southern Africa, maize grain constitutes one-fourth of daily calories, which is the highest proportion globally (2).

Maize grain yield in SSA is among the lowest in the world (2). Unpredictable environmental stresses further diminish its productivity (3). Maize lethal necrosis (MLN) first emerged in Bomet County, Kenya, in 2011 and rapidly spread to neighboring countries (4). On average, MLN reduces grain yield by one-fourth, although complete crop loss from severe outbreaks is not uncommon (5). Almost all commercial germplasm released prior to the emergence of the disease was susceptible (4, 6).

Maize chlorotic mottle virus (MCMV), along with a potyvirus such as sugarcane mosaic virus (SCMV), causes MLN (4, 7, 8). Commercial maize lines grown in warm regions where SCMV is common exhibit varying levels of tolerance to this virus (9). Even low levels of SCMV in the plant can trigger MLN when MCMV is introduced by the transmitting vectors (4). Currently, only a limited number of natural sources of resistance are known, and the specific genes responsible for this resistance remain unidentified (4).

**Fig. 1.**
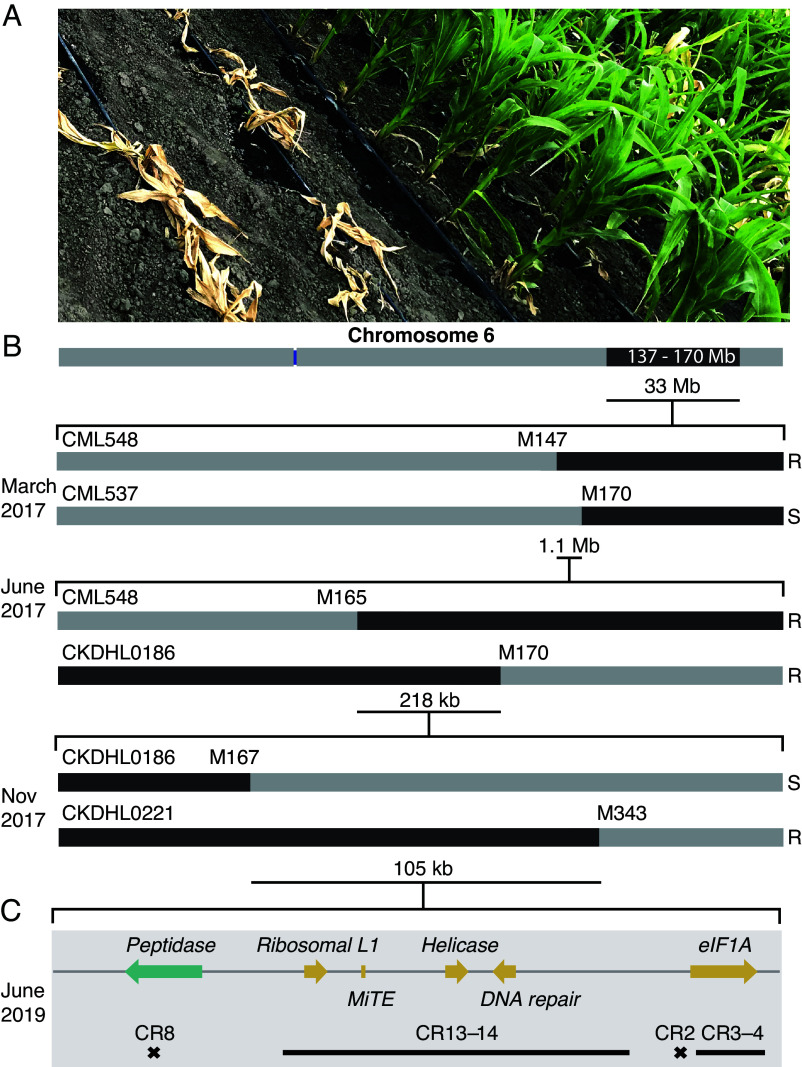
Fine mapping of QTL for resistance to MLN and identification of causal gene. (*A*) MLN-susceptible inbred line CML511 without (*Left*) and with the KS23-6 QTL (*Right*) after MLN inoculation in Naivasha, Kenya. (*B*) Narrowing of the genetic interval in successive rounds of phenotyping and genotyping in maize populations. Dark bars represent the KS23-6 genome; lighter bars represent susceptible, recurrent parents. R, resistant; S, susceptible. (*C*) The 105 kb interval with annotated subregions and CRISPR (CR) designs to identify the causal subregion for MLN resistance. CR with a single number denotes one guide-RNA for introducing mutations and a range denotes flanking guides for target region dropout. Crosses represent frameshift mutations and bars represent dropouts.

A tropical maize line, KS23-6, developed at Kasetsart University in Thailand, exhibited exceptional resistance to MLN (Fig. 1*A*). This resistance, which was recessively inherited, was attributed to a quantitative trait locus (QTL) mapped to a broad interval on chromosome 6 (4, 10–12). We refer to this QTL as the *maize lethal necrosis susceptibility locus 1* (*qMLNS1*). The MLN resistance allele at the *Mlns1* locus has been introduced into a range of inbred lines that have been made available to breeders, but many popular elite lines continue to be susceptible (13).

Conventional breeding to incorporate resistance from an exotic source is a lengthy process, and undesirable genes from the donor parent often persist in the converted lines, resulting in yield drag (14, 15). Developing methods that overcome these challenges would significantly expedite the development of MLN-resistant elite maize lines (15, 16).

In this report, we describe the identification and validation of the causal gene for MLN susceptibility within *Mlns1*, which encodes a peroxisomal peptidase. We demonstrate its function by knocking it out in elite, MLN-susceptible lines from SSA, followed by field evaluation in Kenya. This finding enables the direct editing of MLN resistance into elite maize lines, eliminating linkage drag and significantly reducing the time required to release improved hybrids to farmers.

## Results

### Genetic Fine Mapping of *qMLNS1* for MLN Resistance.

To fine map MLN resistance, we evaluated segregating plants across multiple populations generated from crosses between MLN-susceptible elite CIMMYT lines and donor lines exhibiting resistance (*SI Appendix*, Table S1). MLN inoculation was administered 4 wk after planting in an isolated nursery in Naivasha, Kenya (17, 18). Two weeks after inoculation, disease progression was evaluated weekly over 4 wk using a scale ranging from 1 (resistant) to 9 (susceptible) (https://mln.cimmyt.org). The resistant and susceptible plants were qualitatively distinguishable starting at 4 wk after inoculation.

Before initiating crosses, we routinely screened the inbred lines for homozygosity using 15 markers spread across all ten chromosomes (*SI Appendix*, Table S2). The C6QTL had been mapped to a broad interval (11). Our initial signal detection with several CIMMYT populations gave an estimate of ~33 Mb for the QTL interval (*SI Appendix*, Fig. S1*A*). We developed 15 new F_2_ populations from crosses of the MLN-susceptible, elite CIMMYT lines with the MLN resistance donors KS23-5 and KS23-6 (*SI Appendix*, Table S1). The parents, along with 40 to 50 F_2_ plants from each population, were screened with 68 markers spanning the QTL interval to identify 15 to 20 polymorphic markers for fine mapping (Fig. 1*B* and *SI Appendix*, Table S3).

Screening of F_2_-derived F_3_ progenies helped narrow the interval to 1.1 Mb (Fig. 1*B*). Custom SNP markers and screening of additional progeny further aided in reducing the interval size to 105 kb, which contained open reading frames (ORFs) for the *peptidase* (Zm00001eb291760), *ribosomal L1* (Zm00001eb291780), *helicase* (Zm00001eb291790), *DNA repair* (Zm00001eb291800), and *eukaryotic translation initiation factor* (*eIF1A*) (Zm00001eb291810) genes (Fig. 1*C* and *SI Appendix*, Tables S1 and S4). Although we measured resistance phenotypes on a semiquantitative scale, *qMLNS1*, because of its large effect, segregated as a single locus (*SI Appendix*, Fig. S1 *B* and *C*).

At this point, *eIF1A* was our top candidate for MLN resistance based on previous studies where mutations in this family of proteins had been reported to confer virus resistance across a wide array of plant species (8, 19, 20). A *miniature inverted-repeat transposable element* (*MiTE*) was also present. *MiTEs* were previously reported to influence the regulation of *cis* genes, extending up to 70 kb away (21, 22). We selected all the candidate genes for validation using the CRISPR-Cas9 technology.

### Selection of Inbred Lines for Candidate Gene Validation and Whole Genome Sequencing.

We decided to test the candidate genes directly in CML536, an elite maize line from SSA that was highly susceptible to MLN. This line, along with three other MLN-susceptible lines CML543, CKL05004, and CKL05022, was the parent of two popular, heat- and drought-tolerant three-way cross hybrids that were widely cultivated in Bomet County, Kenya, and were released just before the emergence of MLN (*SI Appendix*, Fig. S2*A*). We plan on reintroducing gene-edited, MLN-resistant hybrid variants to the focal point of the MLN outbreak. This approach will illustrate that, once the causal gene is known, gene-edited, MLN-resistant versions of susceptible lines can be regrown in the field within a 2 to 3-y timeframe. The edited lines can also be crossed to susceptible lines for forward breeding.

**Fig. 2.**
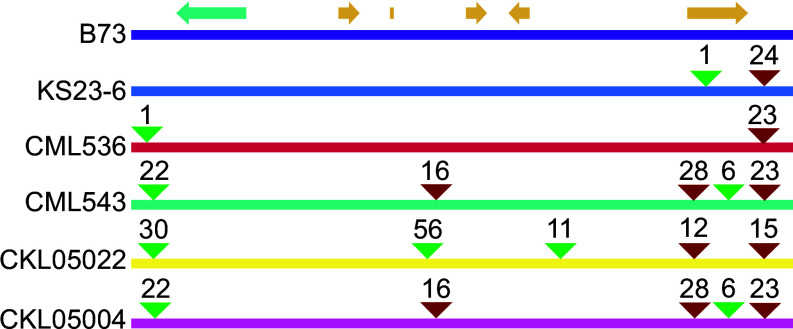
Genome organization of the mapped interval. Structural polymorphisms of the MLN resistance donor (KS23-6) and four elite CIMMYT lines are compared to the B73 reference genome. Triangles colored green and red denote insertions and deletions, respectively. The rounded numbers above the triangle are for the size in kb. Only polymorphisms exceeding 1 kb are shown. The genetic interval spans approximately 105 kb in the B73 reference genome, 83 kb in KS23-6 and CML536, 66 kb in CML543 and CKL5004, and 176 kb in CKL05022. On top are the approximate locations of the predicted ORFs from Fig. 1*C*.

Before proceeding with gene editing, we obtained whole genome sequences for all four lines as well as for CKDHL0186, another susceptible elite line used to create the mapping populations, and the resistance donor KS23-6 (*SI Appendix*, Tables S1 and S5) (23). The fine-mapped interval exhibited structural variability across the lines; however, KS23-6 and CML536 were notably similar (Fig. 2). In both lines, the interval was 22 kb smaller than the reference genome of B73 (24). CML543 and CKL05004 were structurally similar, while CKL05022 was unique.

**Fig. 3.**
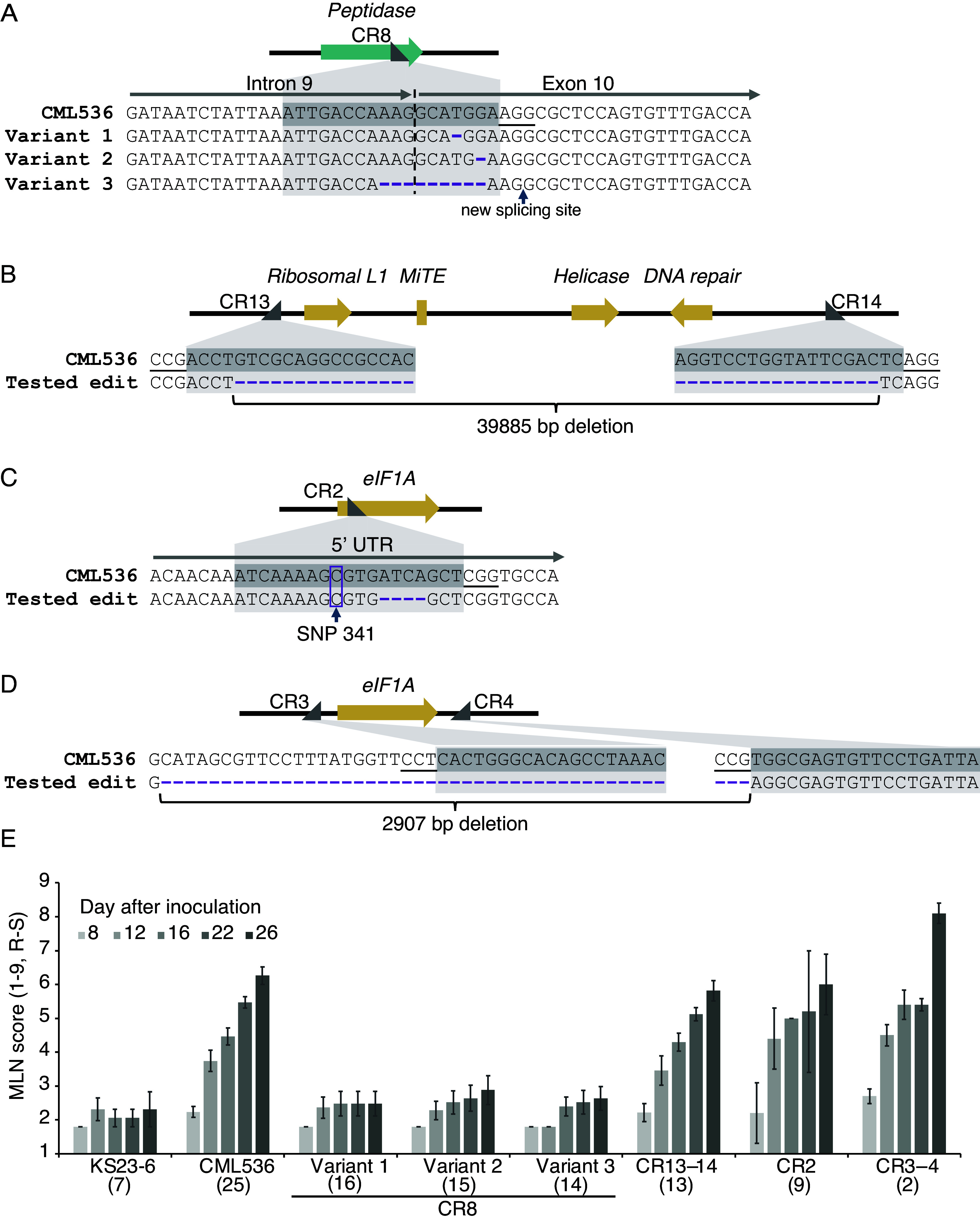
Gene-edited variants and their response to MLN inoculation. (*A*) Three *peptidase* gene variants with nucleotide deletions represented by purple dashes are shown. For the variants 1 and 2, single nucleotide deletions in the tenth exon caused frameshift mutations, whereas a longer deletion in variant 3 spanning the intron/exon junction resulted in an in-frame deletion of three amino acids (*SI Appendix*, Fig. S3). (*B*) An edited variant with a 40 kb deletion encompassing several open reading frames and a *MiTE*. (*C*) An edited frameshift variant of *eIF1A*. SNP341 corresponding to a change from cytosine in CML536 to a thymidine in KS23-6 is highlighted. (*D*) An edited variant of *eIF1A* with the gene and flanking regions deleted. (*E*) Response of the edited variants for the *peptidase* gene (CR8), frameshift (CR2) and dropout (CR3-4) variants of *eIF1A*, and the 40 kb (CR13-14) dropout (Fig. 1*C*) in the greenhouse to MLN inoculation. Numbers in parentheses are for screened plants. Data are presented only for homozygous plants (approximately 25% of the total).

Genome sequences proved essential for designing line-specific guide-RNAs. For example, a unique guide RNA, CRISPR-8 (CR8), designed to edit the *peptidase* gene via nonhomologous end joining was identical in three of the four target lines but contained a polymorphism in CKL05022, prompting us to design a separate guide RNA specific to this line (CR8.1) (Fig. 3 and *SI Appendix*, Table S6).

Routine transformation of tropical maize germplasm had not yet been achieved when we undertook this project. We demonstrated that all four selected lines could be transformed with perfect efficiency using Corteva Agriscience’s genotype-independent transformation system, which incorporates cell morphogenesis genes under the control of specific promoters in the transformation vector (*SI Appendix*, Fig. S2*B*) (25, 26).

### CRISPR-Cas9 Editing of Candidate Genes in the Fine-Mapped Interval.

The process of tissue transformation and progressing to the production of T_2_ seed, where homozygous plants can be tested for MLN resistance, takes approximately a year and a half. Therefore, we decided to simultaneously knock out all the candidate ORFs or subregions within the 105 kb interval, generating six edited alleles (Fig. 3 *A*–*D* and *SI Appendix*, Table S6).

We designed a single guide RNA for the *peptidase* gene to introduce frameshift mutations (Fig. 3*A*). A pair of guide RNAs was designed to dropout the entire 40 kb middle section containing three putative ORFs and the *MiTE* (Fig. 3*B*). Three guide RNA molecules were designed to target the *eIF1A* gene: one to introduce mutations near the 5’-end and a pair of guides to completely drop out the gene (Fig. 3 *C* and *D* and *SI Appendix*, Table S6).

Three independent edited alleles of the *peptidase* gene were advanced. Variants 1 and 2 each had a single nucleotide deletion in the ORF, resulting in a frameshift mutation (Fig. 3*A* and *SI Appendix*, Fig. S3*A* and Tables S7 and S8). A 9-nucleotide deletion, which resulted from 3 nucleotides in the 9th intron and 6 in the 10th exon in variant 3, eliminated the intron/exon junction motif “AG” (Fig. 3*A* and *SI Appendix*, Fig. S3 *A* and *B*). Sequencing of the RT-PCR amplicon from the leaf tissue revealed that the mRNA was processed differently in variant 3. Splicing occurred at the next available “AG” motif, which was located 3 nucleotides downstream of the last missing nucleotide in the genomic copy (*SI Appendix*, Fig. S3*A*). This resulted in a deletion of three amino acids in the coding sequence (*SI Appendix*, Fig. S3 *A* and *B*).

The 40 kb deletion in the central region eliminated three ORFs and the *MiTE* (Fig. 3*B*). With prior knowledge of the role of the eukaryotic translation initiation factors in virus resistance, a unique cytosine-to-thymidine polymorphism (SNP 341) in the 5’-UTR of the *eIF1A* gene in the resistance donor KS23-6 led us to speculate that it might influence the secondary structure of the mRNA and hence its translation efficiency (Fig. 3*C*). One of the two edited alleles had a four-nucleotide deletion downstream of the unique SNP polymorphism, whereas the second had the entire coding region deleted (Fig. 3 *C* and *D*).

### Causal Gene Identification through Screening of Edited Variants for MLN Resistance.

When inoculated with the MLN viruses, homozygous T_2_ plants for each of the three edited variants in the *peptidase* gene exhibited strong resistance, comparable to the resistance donor KS23-6 (Fig. 3*E*). In contrast, plants with the 40 kb deletion containing three ORFs and *MiTE*, or both the edited *eIF1A* alleles, were equally susceptible to MLN as the parental line. Therefore, the *peptidase* emerged as the sole candidate responsible for MLN susceptibility within the *Mlns1* locus (Fig. 3*E*).

**Fig. 4.**
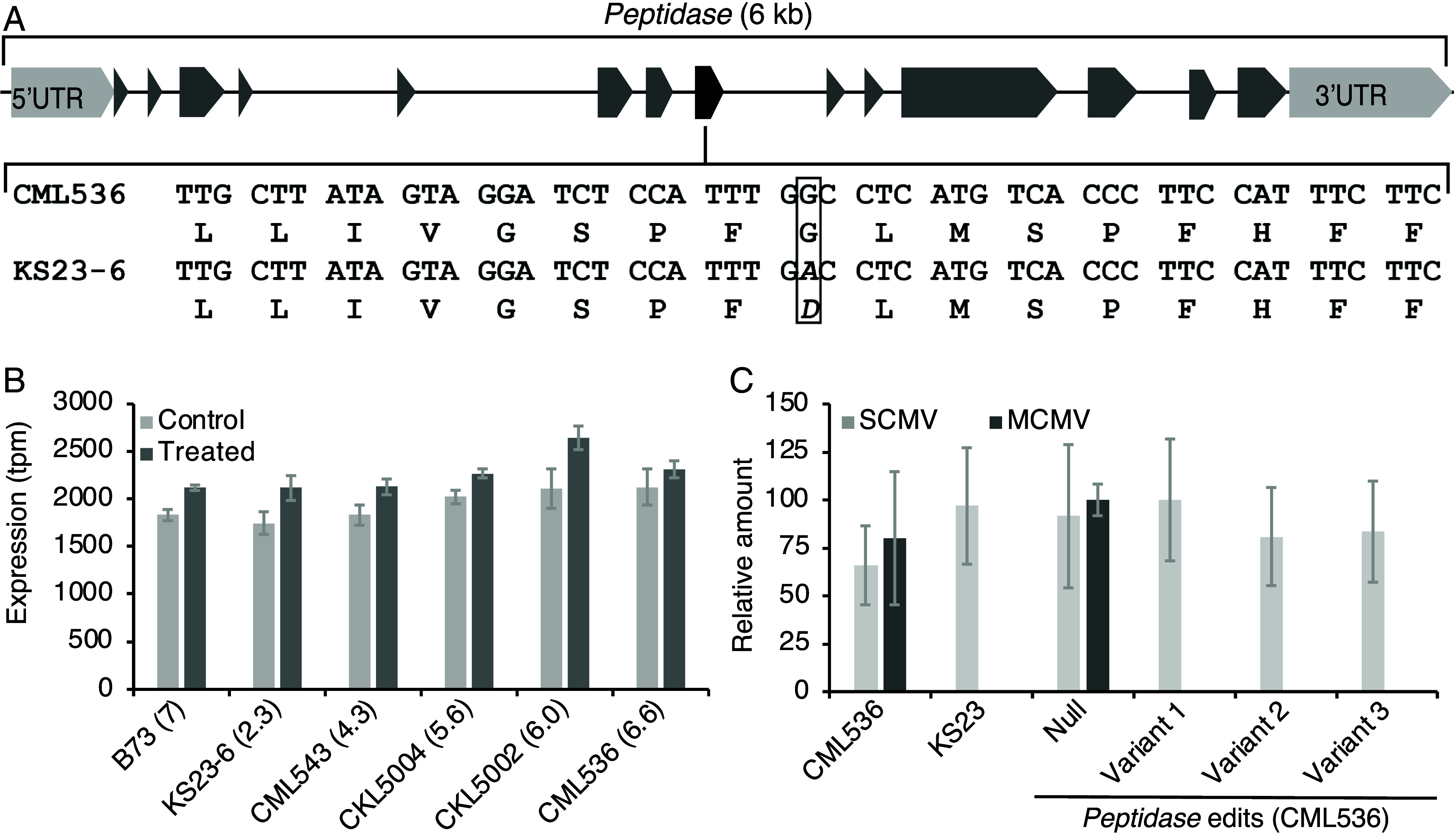
*Peptidase* characteristics, gene expression, and mode of action. (*A*) Structure of the *peptidase* gene. Exons are depicted by solid triangles and trapezoids. A single nucleotide polymorphism unique to the KS23-6 line is highlighted in the 8th exon with the corresponding portion of the open reading frame and translation product shown below. The sense strand of the *peptidase* gene was oriented toward the centromere (Fig. 1*C*) and is flipped horizontally. Sequences for nucleotides and amino acids (242 to 258) around the polymorphic site (boxed) in the 8th exon are depicted at the *Bottom*. (*B*) *Peptidase* gene expression in maize lines. Numbers in parentheses are MLN resistance scores. (*C*) Effect of edited *peptidase* on MCMV and SCMV amounts in maize leaves as measured by qPCR 2 wk after MLN inoculation. Variants 1 and 2 were frameshift knockouts and variant 3 had an in-frame deletion of three amino acids (Fig. 2*A* and *SI Appendix*, Fig. S3 *A* and *B*).

The *peptidase* is a 6 kb, single-copy gene containing 14 exons (Fig. 4*A*). The predicted ORF produced a protein of 722 amino acids with a molecular mass of 78.64 kDa and an isoelectric point of 7.73 (*SI Appendix*, Fig. S3 *B* and *C*). In the resistant line KS23-6, a single guanine-to-adenine polymorphism in exon 8 caused a glycine-to-aspartate change at position 250 (G250D) of the predicted protein (Fig. 4*A* and *SI Appendix*, Fig. S3*B*). The peptidase is present in all the angiosperms we examined (*SI Appendix*, Fig. S4*A*). The glycine residue is conserved across species except in the line KS23-6 (*SI Appendix*, Fig. S4*B*). Homology modeling suggested this substitution, which replaces a small, neutral amino acid glycine with a bulkier, negatively charged aspartate, likely altered the protein’s function (*SI Appendix*, Fig. S5).

The expression of the *peptidase* gene was slightly upregulated upon MLN infection but did not correlate with the MLN resistance scores of the inbred lines, suggesting that a threshold level of functional protein was sufficient for susceptibility (Fig. 4*B*).

To determine whether the peptidase impacted one or both causal viruses for MLN, we screened wild-type CML536, its edited variants, and KS23-6 for response to MLN inoculation in the greenhouse. In all lines, SCMV levels remained unchanged (Fig. 4*C*). However, MCMV was barely detectable in *peptidase*-edited variants and the resistant KS23-6 line but was prevalent among unedited siblings and the parental CML536 line. This suggests the peptidase is required for MCMV accumulation. The peptidase protein is prematurely truncated from frameshift mutations in edited variants 1 and 2 (Fig. 3*A* and *SI Appendix*, Fig. S3*A*). The partial protein, lacking the peroxisome targeting signal, was likely degraded by the cytosolic ubiquitin–proteasome pathway (27).

### The Peptidase Protein Is Localized to the Peroxisome.

The predicted peptidase protein ended in a C-terminal tripeptide, serine-lysine-isoleucine (SKI), which was reported to be a low-probability peroxisome targeting signal (28) (*SI Appendix*, Fig. S3*B*). The canonical signal for peroxisome targeting is serine-lysine-leucine (SKL) (29). We thus determined the subcellular localization of the peptidase by expressing three sets of vectors containing combinations of green and red fluorescent proteins with C-terminal SKI or SKL in maize cells followed by laser scanning microscopy. The first set included a red fluorescent protein (RFP) with either the C-terminal SKI or the SKL motif (*SI Appendix*, Fig. S6 and Table S9). Both constructs targeted RFP to similar punctae (*SI Appendix*, Fig. S6).

The second vector set featured N-terminal GFP fused to the peptidase, with either its native C-terminal SKI or the SKL motif. The GFP-peptidase fusion also localized to punctate structures with either targeting signal (*SI Appendix*, Fig. S6).

**Fig. 5.**
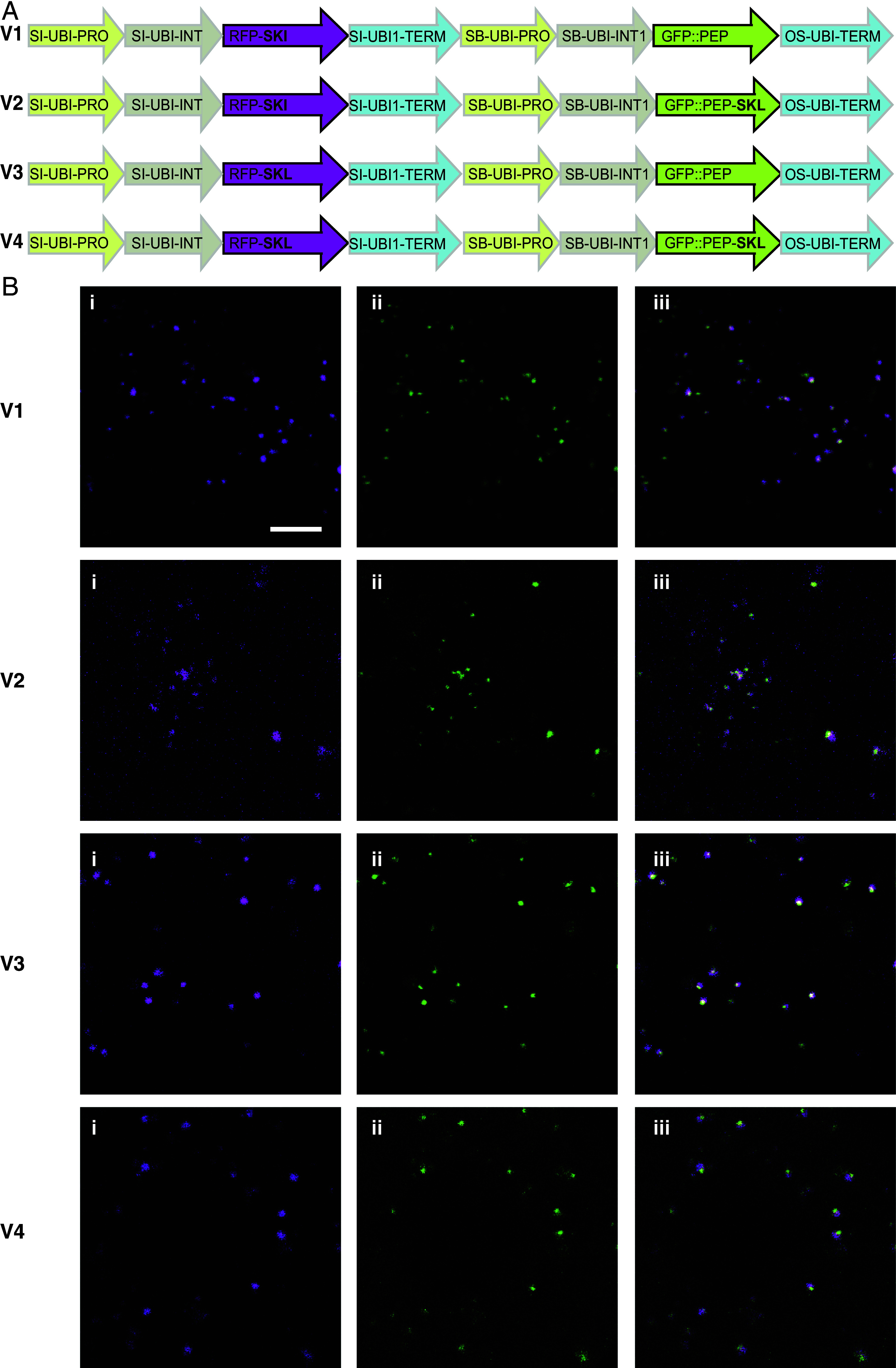
Time-lapse microscopy of subcellular localization of peptidase. (*A*) Vector designs depicting red and green fluorescent protein fusions with peptidase (PEP) with all combinations of C-terminal tripeptide signals SKI and SKL. (*B*) Scanning the cells expressing various vectors with red channel (i), green channel (ii), and overlap of the two (iii). Each horizontal panel corresponds to cells transformed with the corresponding vectors shown in *A*. (Scale bar, 5 µm.) Time-lapse microscopy of subcellular localization and dynamics of peptidase and peroxisomes is available at https://doi.org/10.6084/m9.figshare.28747004.v1.

The third set combined the above two, resulting in four vectors that represented all four combinations of SKI and SKL at the C termini of RFP and GFP-peptidase fusion (Fig. 5*A*). Overlaying fluorescence from red and green channels showed that both SKI and SKL motifs targeted proteins to the same punctate compartments, which we concluded were peroxisomes (Fig. 5*B*). The peptidase protein was targeted to a subregion of the peroxisome lumen (Movie S1) (30). The presence of similar subcompartments within the peroxisome had previously been observed in *Arabidopsis* (31).

### Evaluation of Non-GMO *peptidase* Edits in Field Trials Conducted in Kenya.

**Fig. 6.**
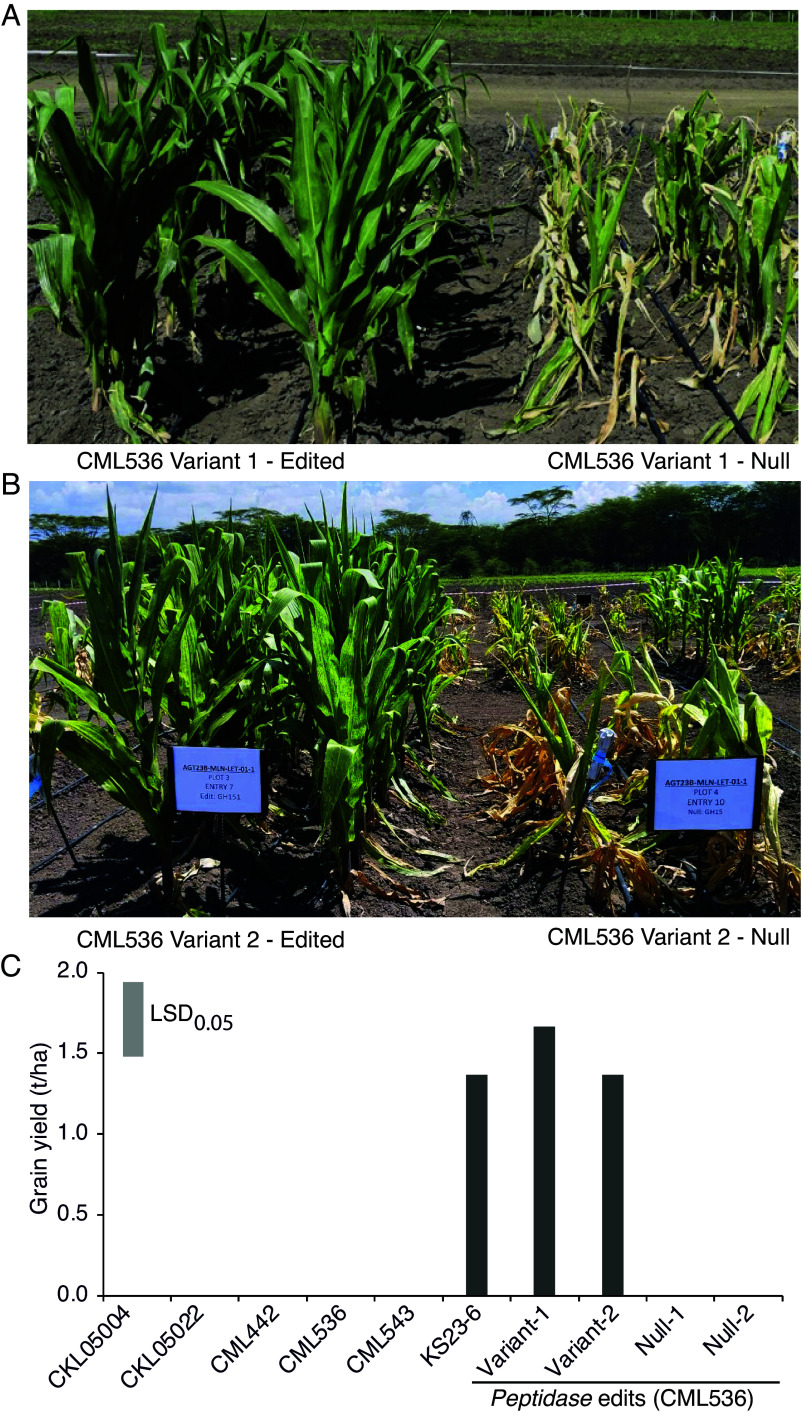
Performance of wild-type and *peptidase*-edited maize lines in the field in Naivasha, Kenya, after MLN inoculation. Edited knockout variants 1 (*A*) and 2 (*B*) along with their nulls (Fig. 3*A*) 4 wk after MLN inoculation. (*C*) Grain yield of elite inbred lines, resistance donor KS23-6, and edited *peptidase* variants with their respective nulls. LSD_0.05_ is least significant difference at *P* = 0.05.

For field trials in Kenya, we confirmed genetic purity and the absence of the transformation vector from the genome of the edited *peptidase* variants (Fig. 3*A* and *SI Appendix*, Figs. S3*A* and S7 and Table S2). Following approval from the Kenyan National Biosafety Authority (NBA) and confirmation that these edits were non-GMO, we conducted open field trials. Under artificial MLN inoculation in Naivasha, the *peptidase*-edited variants were highly resistant, while null segregants lacking the edits succumbed to disease like the parental line (Fig. 6 *A*–*C*).

**Fig. 7.**
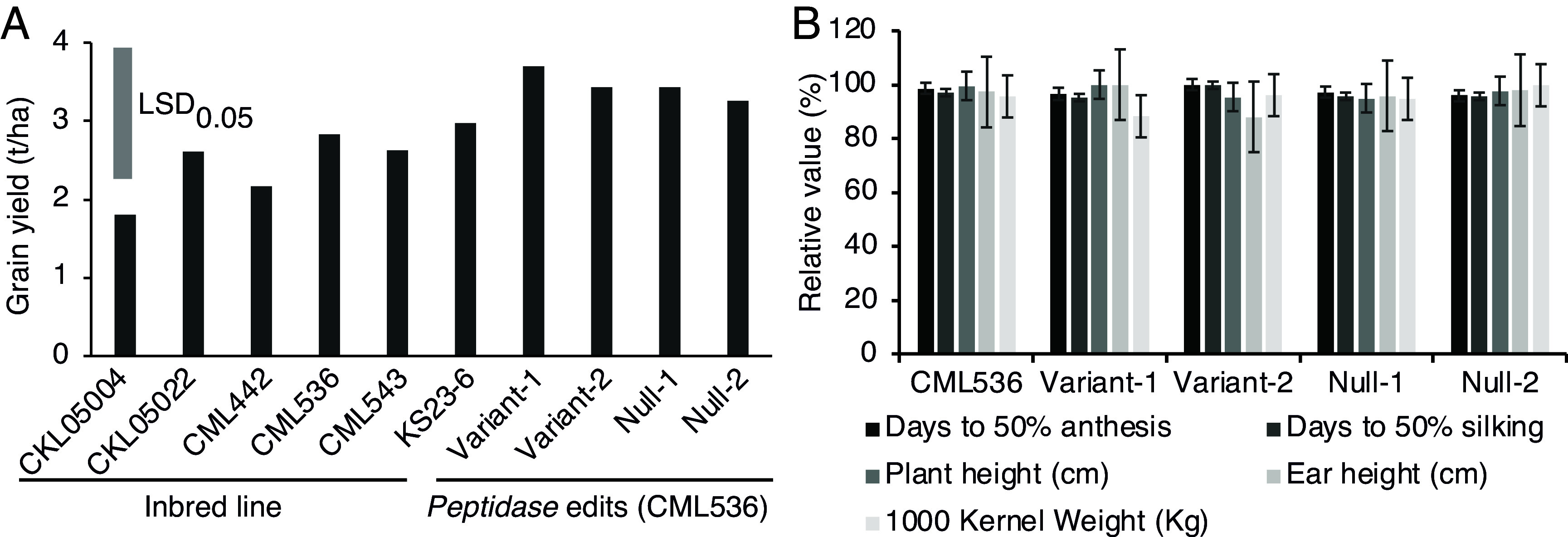
Performance of wild-type and *peptidase-*edited maize lines in the field in Kiboko, Kenya. (*A*) Grain yield. (*B*) Agronomic characteristics. The maximum observed values were days to anthesis and silking, 67; plant height, 220 cm; ear height, 93 cm; and 1,000-kernel weight, 340 g. The MLN disease is absent from the Kiboko environm ent.

In disease-free trials in Kiboko, the grain yield and key agronomic traits, such as days to pollen shed and silking, plant height, ear height, and 1,000 kernel weight, of the edited lines were indistinguishable from the parental CML536 line (Fig. 7 *A* and *B*). This demonstrated that knocking out the *peptidase* gene conferred MLN resistance without any yield penalty.

**Fig. 8.**
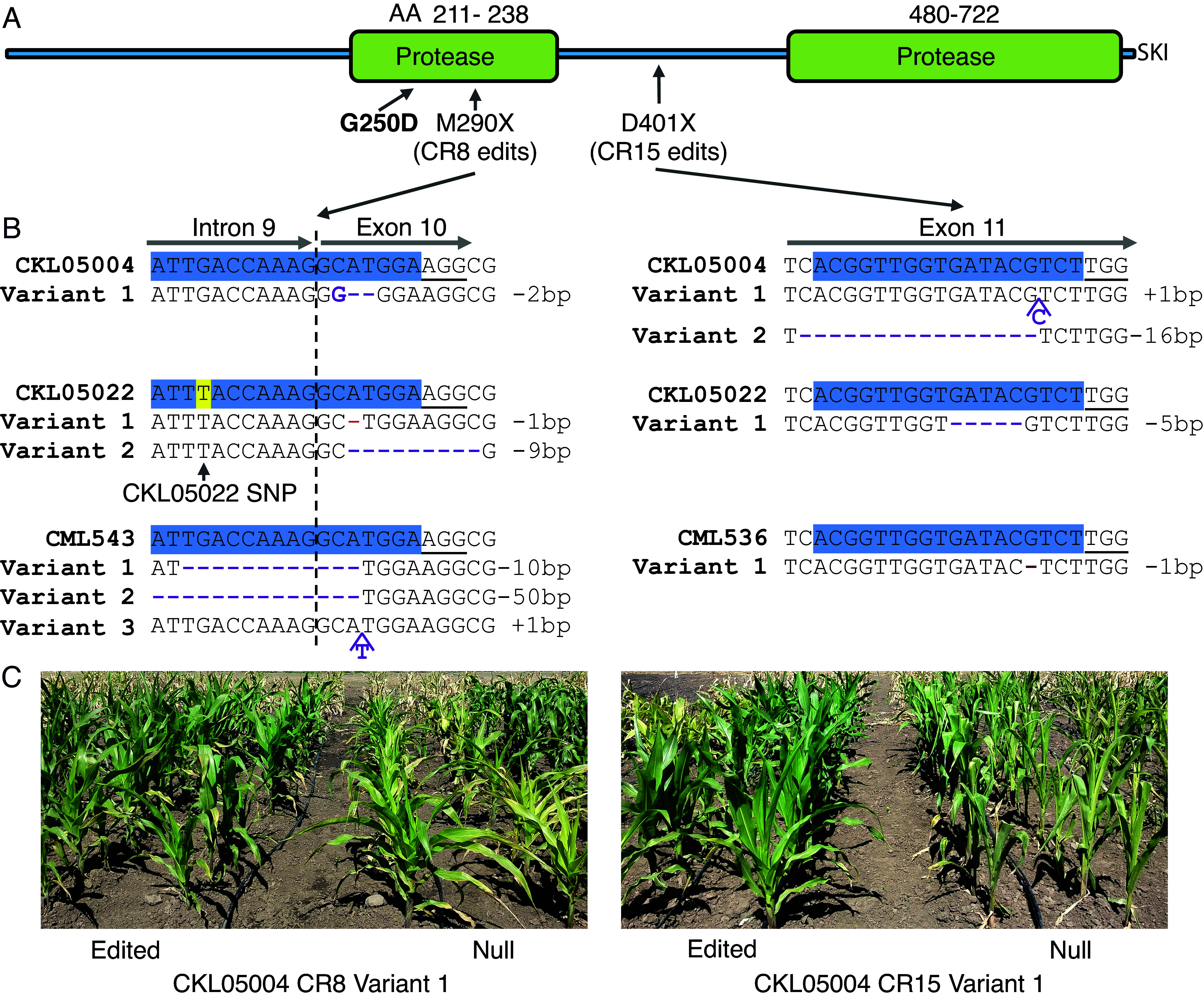
Editing of the *peptidase* gene in elite maize lines from Africa. (*A*) A sketch of the peptidase protein showing two protease domains and the peroxisomal targeting SKI signal. The causal MLN-resistance mutation in KS23-6 (guanosine to adenine, resulting in G250D) is in the first protease domain. Mutations introduced using CR8 and CR15 guides are annotated in the model and shown in sequences below in (*B*). Numbers indicate the corresponding amino acid positions. (*B*) *Peptidase* variant sequences of elite lines edited with CR8 and CR15 guides. A modified CR8 guide (CR8*) was used to introduce edits in CKL05022, accommodating a SNP (shaded yellow) in this line. Mutations in variants are displayed in purple. Hashes represent deletions. Substitutions are in bold purple. Insertions are shown with the respective nucleotides with caps. (*C*) Early results from field testing of the edited variants in the elite line CKL05004 3 wk after MLN inoculation.

We have generated edits in the remaining three lines: CML543, CKL05022, and CKL05004 (Fig. 8 and *SI Appendix*, Tables S6–S8). These edited variants were also confirmed by the Kenyan NBA as non-GMO. Initial results from two independently edited variants of another line, CKL05004, were as expected. Both variants showed strong resistance to MLN (Fig. 8*C*). The three-way-cross hybrids are being currently produced from the edited lines and will be tested at multiple locations in Kenya once ready, paving the way for commercial deployment.

## Discussion

Disruption of a unique peroxisomal peptidase, which acts as a host susceptibility factor, via CRISPR-Cas9 in elite African maize lines conferred strong MLN resistance without compromising agronomic performance in Kenyan field trials (Figs. 3 *A* and *E*, 6, and 7).

**Fig. 9.**
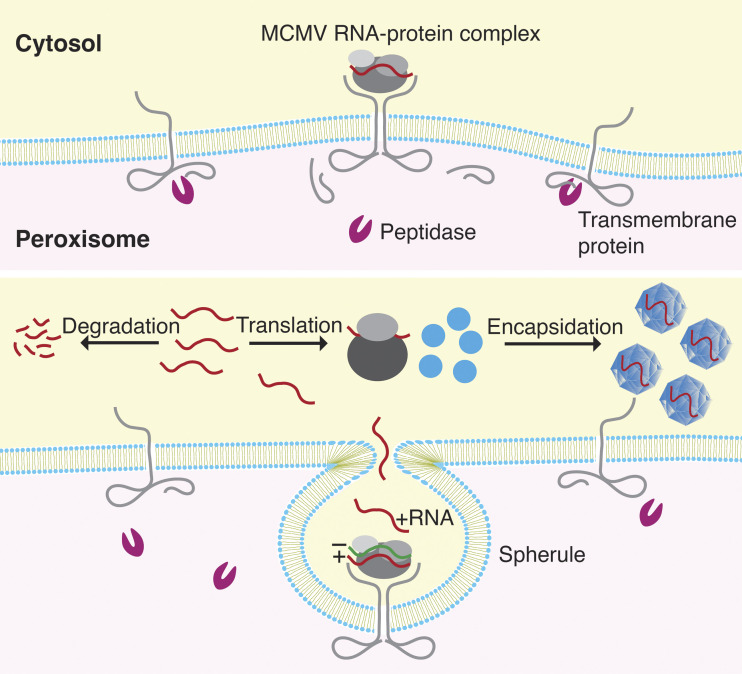
A hypothetical model for the mechanism of action of peroxisomal peptidase in facilitating replication of maize chlorotic mottle virus. After viral entry, the MCMV RNA is translated in the cytosol and then forms a complex with the viral proteins (*SI Appendix*, Fig. S8), possibly including host chaperones. The peptidase processes the luminal domain of the peroxisomal transmembrane protein to activate its cytosolic domain for recognition by the viral RNA–protein complex, thus initiating spherule formation. Whereas peptidase-dependent dimerization is shown in this graphic, it could be a monomer or a multimer that mediates spherule formation. Not to scale.

Viruses evade host defenses by creating replication compartments, known as spherules, within the plasma membrane and the membranes of subcellular organelles (32, 33). Spherules open into the cytoplasm, separating viral replication from the organelle’s luminal contents (Fig. 9). SCMV induces the formation of spherules in the endoplasmic reticulum (ER), whereas tombusviruses like MCMV produce these structures in the peroxisomal membrane (32–37). In agreement, our results demonstrate that the peptidase, which specifically localizes to peroxisomes (Fig. 5 and *SI Appendix*, Fig. S6), selectively promotes MCMV replication while having no effect on SCMV (Fig. 4*C*).

The absence of MCMV in the *peptidase* knockouts corroborates earlier findings that this virus relies on peroxisomal spherules for replication (35) (Fig. 4*C*). Because elite maize lines in SSA possess resistance to SCMV, their agronomic performance is not significantly compromised by this virus alone (Fig. 6*C*) (9).

The mechanism by which MCMV induces spherule formation (38) appears to differ from other tombusviruses (36, 39). The translated product of the first ORF, p33, from the tomato bushy stunt virus (TBSV), the type species of *Tombusviridae*, contains two transmembrane domains (TMDs) near the N terminus. These TMDs were required for peroxisomal localization and subsequent spherule formation (36, 39). However, our analysis showed that the MCMV proteins, except perhaps p7b, a movement protein that localized to plasmodesmata in melon necrotic spot virus, lacked transmembrane domains (*SI Appendix*, Fig. S8*A*) (40). The predicted amino acid sequence of the first MCMV ORF shows no homology with the corresponding p33 protein of TBSV (*SI Appendix*, Fig. S8). The modeled structures of p33 from TBSV and p32 from MCMV were also unrelated (*SI Appendix*, Fig. S8*B*). The p32 protein of MCMV, although important, was not required for either replication or cell-to-cell movement of this virus (41). However, MCMV has been demonstrated to specifically localize to peroxisomes and induce their aggregation (35).

Regardless of which MCMV proteins initiate spherule formation in the peroxisomal membrane, the peptidase appears to act as a critical factor in this process. One possibility is that MCMV proteins and RNA involved in spherule formation interact with the cytosolic domain of a host peroxisomal transmembrane protein (42) (Fig. 9). The activation of the cytosolic domain of the transmembrane protein to bind the MCMV RNA–protein complex requires the processing of its luminal domain by the peptidase. The viral protein–RNA complex then binds the activated cytosolic domain, signaling a conformational change of the membrane. This change paves the way for spherule formation (38). Alternatively, the peptidase may constitutively process the luminal domain, leaving the cytosolic domain continuously activated to interact with viral proteins. The interaction with the viral proteins may signal an alteration in the membrane composition, for example, through recruitment of sterols and phospholipids via the membrane contact sites with the ER, allowing for additional membrane formation (38). The viral protein-transmembrane protein interaction might also lead to localized changes in the luminal matrix to facilitate membrane invagination.

Mutational genetics, the yeast two-hybrid system, or chemical crosslinking could be employed to identify the peroxisomal transmembrane protein responsible for signaling spherule formation. Since the identified protein would need to be validated through mutational genetics, a suitable approach might be to screen transposon-tagged populations for MLN resistance (43). An alternative is to multiplex CRISPR-Cas9 knockouts of the predicted peroxisomal transmembrane proteins (44). Most maize lines, which include experimental lines like B73 and MO17, are susceptible to MLN.

Many reports on virus resistance involve mutations in the eIF proteins, which the virus exploits to translate its genome (8, 20, 45–47). Generally, the viral genome binds eIF4E either through a genome-linked protein or cap-independent translation enhancers (48). However, about a fifth of viruses use 7-methylguanosine (7mG) to cap their genomes, mirroring the cap structure of their host’s mRNA (19). Mutating eIF4E would be ineffective in blocking the translation of the 7mG-capped viral genomes (8, 19). In this study, we introduce an alternative route for virus resistance by targeting spherule formation.

The *peptidase* gene is ubiquitous in angiosperms (*SI Appendix*, Fig. S4). Its conservation across all the examined angiosperms suggests knocking it out could provide resistance to viruses from at least the *Tombusviridae* family in other plant species as well. It is also possible that unrelated peptidases mediate the formation of spherules for other viruses in different cellular membranes.

Knocking out a single gene to confer MLN resistance provides a rapid method to edit susceptible elite lines and return them to the field within 2 to 3 y. This approach also eliminates the risk of yield reduction caused by residual donor parent genes, which would remain a persistent concern with conventionally converted lines (15). Beyond representing a significant advancement in plant virology, our research provides an innovative approach to safeguard grain yields on smallholder farms where MLN remains a persistent threat to maize production.

## Materials and Methods

### Plant Material.

Plant materials were developed at CIMMYT (Kenya) and Corteva Agriscience (Johnston, Iowa), with all field phenotyping conducted in Naivasha or Kiboko in Kenya. The inbred lines KS23-6 (Ames 30911) and KS23-5 (Ames 30910) were procured from GRIN (www.grin-global.org).

### Field Experiments to Screen for MLN Resistance.

Gene-edited lines, along with resistant and susceptible checks, were evaluated under artificial inoculation at the MLN screening facility in Naivasha, Kenya, and under optimal conditions at the research station in Kiboko, Kenya (49). See *SI Appendix* for field experiment details. Data analysis for individual sites was performed using META-R software (50). Similar practices and conditions were used for all the fine-mapping experiments.

MLN screening in Naivasha was conducted under artificial inoculation conditions. See *SI Appendix* for details on viral preparation, inoculation and scoring.

### Genotyping with Production Markers.

A modified TaqMan genotyping assay was used (51). See *SI Appendix* for details.

### QTL Analysis.

Marker trait association analysis of CIMMYT populations (*SI Appendix*, Table S1A) refined the ~90 MB interval to an estimate of ~33 MB (Fig. 1*B*). For this analysis, a single marker association test was used with the MLN1-5 scores analyzed independently. The association tests were performed for each marker that passed the quality control step (filtering by allele frequencies between 0.05 and 0.95). For each marker, a standard *t* test statistic was calculated to reflect the association between the marker genotypes and the phenotypic data, and the corresponding log(1/p) was reported. The log(1/p) = 3 (*P*-value = 0.001) was used as the threshold for significance. The F-test was used for analysis with 3 genotypes, for example in an F_2_ where AA, BB, and AB (H) genotypes can occur at the locus.

### Genotyping with Custom Markers for Fine Mapping.

Genotyping was carried out using KASP^TM^ (Kompetitive Allele Specific PCR, www.biosearchtech.com) technology. See the *SI Appendix* for details.

### Whole Genome Optical Mapping and Sequencing.

DNA was extracted for optical map construction from 0.5 g of leaf tissue using the Bionano Prep™ Plant Tissue DNA Isolation kit as previously described (52). Direct label and staining were also performed as described by Hufford et al., using the DLS Kit (Bionano Genomics Cat.80005). Labeled and stained DNA molecules were loaded onto a Bionano chip flowcell, separated by electrophoresis, imaged, and digitized in the Saphyr System according to the manufacturer’s recommendations. Data visualization, processing, and DLS map assembly were conducted using the Bionano Genomics software Access, Solve and Tools. Solve v1.3 and Tools v3.3 were used for CML536, CML543, CKL050004, CKL05022, whereas Tool version 1.4 was used for CKDHL0186. Molecule and Genome map assembly data statistics and results are shown in *SI Appendix*, Table S5.

For CLR PacBio Sequencing (www.pacb.com) using uHMW DNA, sequencing libraries were constructed according to Pacific SMRTbell® Express Template Prep Kit 2.0 protocol and run in a PacBio Sequel instrument using Sequel® Binding Kit 1.0 (CKL05004, CKL05022, and CML543), 2.0 (CKDHL0186), or 3.0 (CML536). Raw data information is included in *SI Appendix*, Table S5. KS23-6 sequencing was completed using an earlier version of the Pacific SMRTbell® Express Template Prep.

### Genomic Interval Analysis and Gene Identification.

Gene models from B73 v4 maize were used in the fine-mapping interval and verified against the KS23-6 and CML536 maize genome sequences before guide RNA design (24).

### Vector Designs and Plant Transformation for Genome Editing.

Gene editing constructs (*SI Appendix*, Table S6), *Agrobacterium* transformation and NextGen sequencing of gene-edited lines were as described previously (53–56). Briefly, the construct contained a ZmAxig1pro:ZmWus2 cassette, a ZmUbi1pro::ZmOdp2 cassette, a ZmUbi1pro:SpCas9 cassette, a ZmU6pro:gRNA cassette for appropriate guide RNA(s), and a ZmUbi1pro::ZmAa1 cassette, and transformed into 2 mm immature maize embryos extracted from the ears approximately 2 wk after pollination via the LBA4404 (THY-) strain of *Agrobacterium tumefaciens* containing the pVIR9 plasmid (25). This was followed by selection on G418 media (www.thermofisher.com) to select the transformed somatic embryos.

### Genotypic Characterization of Edits by Sequencing.

Gene edits were characterized in T_0_ and T_1_ plants as described in Che et al. (57). The primers used to amplify targeted locus are shown in *SI Appendix*, Table S7.

### Genotypic Characterization of Edits to Identify Gene Variants.

For characterization of edits in T_2_ and later generations, DNA was extracted from 8, 6.25 mm leaf punches by alkaline lysis with mechanical disruption. Following extraction, samples were neutralized for PCR.

TaqMan primers and probes were designed using Applied Biosystems Primer Express Software (www.thermofisher.com). Primers amplified the targeted edit region, while probes specifically detected individual edits (*SI Appendix*, Table S8). A variant assay was developed to target distinctive sequences upstream of the edit, allowing for differentiation of identical edits across different genotypes. See *SI Appendix* for details on digital PCR used for genotyping.

### Phenotyping of Gene-Edited Variants in Controlled Environments.

MLN growth chamber assays were used for phenotyping of the edited plants (initial T_1_ characterization, T_2_ confirmation and qPCR for viral assays on T_4_ plants) with the following modifications: The Ohio SCMV isolate was used with a Hawaii isolate of MCMV in a 4 parts SCMV to 1 part MCMV ratio in 10 mM potassium phosphate, pH 7, buffer containing 0.1% Tween-20 (58). Carborundum was mixed into the buffer to a concentration of 1 mg/mL and plants were directly rub-inoculated with 20 strokes per leaf 21 to 23 d after sowing. At USDA in Wooster, Ohio, phenotypic evaluations were done 35 to 50 d after sowing as previously described every 3 d for a total of 4 to 5 times (10, 58).

### Quantitative PCR for Measurement of Viral Titer in Gene-Edited Variants.

RNA was extracted from maize leaf tissue using the Qiagen RNeasy Plant Mini Kit (Qiagen Cat. No. 74904) and cDNA was synthesized using the Applied Biosystems High-Capacity cDNA Reverse Transcription Kit (Thermo Fisher Cat. No. 4368814, www.thermofisher.com) following the manufacturer’s protocol. qPCR was performed using the IDT’s PrimeTime™ Gene Expression Master Mix (IDT Cat. No. 1055770, www.idtdna.com) on a Qiagen Rotor-Gene Q instrument, with MCMV primers targeting the TG1 Coat Protein and SCMV primers designed against the genetic region corresponding to the Coat Protein. Technical replicates along with negative and positive controls that were validated against a standard curve were run using standard two-step cycling conditions at 95 °C for 3 min, (95 °C for 15 s, 60 °C for 30 s) × 45. Cq values were averaged across technical replicates and converted to estimated copy numbers.

### RT-PCR and Sequencing for Characterization of Edited Variants.

RNA was extracted using the RNeasy RNA purification kit (Qiagen®). After treatment with DNase I (# 11119915001, www.sigmaaldrich.com), and first-strand cDNA was synthesized according to the manufacturer’s instructions (Thermo Fisher #R1362). A translation initiation factor gene was used as a housekeeping gene. cDNA synthesis without reverse transcriptase was used as a negative control. The synthesized cDNA was used for transcript quantification and sequencing. See *SI Appendix* for details on RNA quantification and sequencing.

### Vectors and Transformation for Subcellular Localization of the Peptidase Protein.

Seeds of Corteva Agriscience maize inbred line PH1V69 were used for the transformation experiments. In vitro seed germination, preparation of leaf tissue explants, and the general *Agrobacterium*-mediated leaf transformation method was carried out as previously described (59). Briefly, 15-d-old seedlings were pretreated at 45 °C/70% RH for 2 h, then 2.5-cm-long leaf base segments were harvested from the seedlings and used to prepare leaf tissue explants for transformation. The *Agrobacterium* suspension from each vector was prepared in 50 mL tubes. Each tube contained 20 mL of infection medium, 40 µL of 100 mM acetosyringone, 0.02% (v/v) BREAK-THRU S 223 and Agrobacterium with a final OD_550_ of 0.6. Leaf tissue explants from five seedlings were added to the 50 mL tube containing freshly prepared *Agrobacterium* suspension. Leaf tissues were then plated onto solid cocultivation medium and incubated at 21 °C in the dark for 1 d and then transferred to the resting medium and cultured at 28 °C in dim light for 3 d. Four days after infection, leaf tissues were collected for microscopy and image analyses.

An entry vector was modified by PCR to include either the SKI or SKL C-terminal fusion to the AC-TagRFP. The ZM-peptidase was assembled from 5 gBlocks™ Gene Fragments (*SI Appendix*, Table S9) synthesized at Integrated DNA Technologies (Coralville, Iowa, www.idtdna.com), a vector backbone containing an origin of replication and kanamycin selection and using NEBuilder® HiFi DNA Assembly Master Mix while following the manufacturer’s recommended protocol. The ZM-peptidase targeting signal was altered to SKI using PCR. These two vectors were then used to generate the AC-GFP1 fused to ZM-peptidase constructs with either the SKI or SKL targeting signal and seamless assembly using PCR products. Four entry vectors containing both fluorescent marker genes with each respective targeting peptide were constructed by PCR amplification of three fragments, the TagRFP(SKI) or TagRFP(SKL) expression cassette region, a clone containing the SI-UBI1 TERM+ SB-UBI PRO+ SB-UBI INTRON1, and the appropriate AC-GFP:linker:ZM-peptidase expression cassette. LR Clonase™ II Plus enzyme from Invitrogen™ was used with destination vector and each respective entry vector to assemble the final constructs for transformation using the Invitrogen™ protocol, allowing the reaction to complete at room temperature overnight. All clones were sequence-confirmed using Azenta’s Plasmid-EZ Whole Plasmid Sequencing.

### Microscopy for Subcellular Localization.

Calli were placed in chambered coverslips (Lab-Tek®, #155411), covered in liquid media, and imaged on a Leica Stellaris5 Confocal microscope in LAS X 4.6.1.27508 software. Images were collected in sequential-by-line mode to minimize crosstalk. Line average was set to 2. Tag-RFP was excited at 561 nm and collected from 570 to 620 nm. Ac-GFP was excited at 485 nm and collected from 500 to 550 nm. Pinhole was set to 1 Airy Unit at reference wavelength 580 nm. Images were collected through a HCX PL APO 63×/1.30 GLYC objective onto HyD S detectors. The pixel size was 0.09 μm.

FIJI was used for linear contrast adjustment, image cropping, and addition of scale bar (60).

For time-lapse supplement, images were collected as above at 5-s intervals for 5 min. The raw data are available at https://doi.org/10.6084/m9.figshare.31952067.

### Molecular Characterization of Edited Variants to Confirm the Non-GMO Status with the NBA of Kenya for Open Field Trials.

T_2_ plants from the 3 variants of the line CML536 that showed efficacy for MLN resistance were used to verify the absence of vector DNA sequences, assayed to confirm the variant genotype, and tested for genetic purity using a panel of markers located across the maize genome (*SI Appendix*, Fig. S7 and Table S2). Two of these 3 variants (Variants 1 and 2 with 1 bp deletions) were submitted in the application to the Kenya NBA for consideration as non-GMO. Analysis was conducted using Next Generation Sequencing-based Southern-by-Sequencing (SbS) (61). Plants with no detected vector-derived DNA were selected and advanced. For Variants 1 and 2 in CML536, it was necessary to generate additional seed, so the procedures above were used on homozygous null and edited T_3_ plants; the resulting T_4_ seed was then bulked for shipping to Kenya. For the second round of editing, and second application to Kenya NBA, which included the additional three lines, these procedures were done on T_2_ plants: one variant in CML536, 3 variants in CML543, one variant in CKL05022 and 3 variants in CKL05004 (Fig. 8). These were approved and sent to Kenya for phenotyping and seed increase.

Additional details on materials and methods are presented in *SI Appendix*.

## Supplementary Material

Appendix 01 (PDF)

Movie S1. Time-lapse microscopy of subcellular localization and dynamics of peptidase and peroxisomes corresponding to Figs. 5 and S6. The peptidase protein is shown targeted to a subregion of the peroxisome lumen. (*https://doi.org/10.6084/m9.figshare.28747004.v1*)

## Data Availability

The raw whole-genome sequencing data for the elite maize lines CML536, CML543, CKL05004, CKL05022, and CKDHL0186 and the resistance donor KS23-6 are available at https://hdl.handle.net/11529/10549222 (23). The time-lapse microscopy data and high-resolution versions of the supplemental movie are available via Figshare at https://doi.org/10.6084/m9.figshare.28747004.v1 (62). All other data are included in the manuscript and/or supporting information.
