# Supplementary material for: Targeted knockout of a host peroxisomal peptidase confers field resistance to maize lethal necrosis
Source: Proc Natl Acad Sci U S A. 2026 Apr 30;123(18):e2535202123. doi: 10.1073/pnas.2535202123 (PMC13142949; doi:10.1073/pnas.2535202123)
Supplement: Supplementary file 1 — Appendix 01 (PDF) [file pnas.2535202123.sapp.pdf]

Supporting Information Appendix

Materials and Methods

Targeted knockout of a host peroxisomal peptidase confers field resistance to maize lethal necrosis

Jung et al.

dhuggaks@gmail.com

Field experiments to screen for MLN resistance

Experiments were conducted in a 4 × 6 alpha lattice design with three replications for each environment. Entries were planted in two-row 5 m long plots with inter-row spacing of 0.75 m and plant-to-plant spacing of 0.25 m. Initially, two seeds were sown per hill, and thinned to one plant two weeks after emergence, resulting in a final population density of approximately 53,000 plants/ha. Harvested ears from each plot were weighed, and grain yield was calculated using field weight and grain moisture content adjusted to 12.5%.

Maize Chlorotic Mottle Virus (MCMV) and Sugarcane Mosaic Virus (SCMV) were mass-produced in separate greenhouses using established inoculum preparation and inoculation protocols (www.mln.cimmyt.org). The first inoculation was done at V5 stage (four weeks after planting), followed by a second inoculation one week later. Disease severity was rated on a 1–9 scale (resistant to susceptible) every 10 days starting two weeks post-inoculation, totaling four to five scorings (MLN1–MLN5) (1).

Genotyping with production markers

DNA was extracted with a sodium hydroxide-based method followed by up to 200-fold dilution and transferred to 384-well array tape LGC Biosearch Technologies (www.biosearchtech.com). Master mix containing primers, probes and TaqDNA polymerase was added, the arrays were sealed and run on hydrocycler (LGC Biosearch Technologies) for 40 cycles. The completed assay signal was read on an Araya reader (LGC Biosearch Technologies) and genotype calls were assigned using proprietary software that visualizes the data in the form of a scatter plot and allows clustering by fluorescence values for allele calling.

Genotyping with custom markers for fine mapping

DNA was extracted from lyophilized leaf tissue (8 6.25 mm punches/sample) using a modified CTAB-based extraction method (2). DNA was dissolved in 50 µl of nuclease-free water and diluted ten-fold to a final amount of 30-50 ng for genotyping. Genotyping was carried out using KASP™ (Kompetitive Allele Specific PCR, www.biosearchtech.com) technology with the KASP-TF V4.0 2X Master Mix, 96/384, Standard ROX (LGC Biosearch Technologies Cat. No. KBS-1050). For each SNP marker, one common primer (C) and two allele-specific primers (A1, A2) were designed and tagged with either FAM (GAAGGTGACCAAGTTCATGCT) or HEX (GAAGGTCGGAGTCAACGGATT). A total of 0.8 µl of Primer Mix (12 µM A1, 12 µM A2, 30 µM C) was used per reaction.

Genotyping PCR was carried out in a 384-well reaction setup (6 µl total volume, 2.42 µl ddH_2O, 2.5 µl KASP Master Mix, 0.08 µl Primer Mix, and 1 µl DNA). PCR cycling conditions were as follows: initial denature 94 °C for 15 m, 10 cycles of touchdown (94 °C for 10 s, 65 ºC - 0.5 ºC per cycle for 20 s, 72 ºC for 40 s), 35 cycles with annealing at 57 ºC (94 °C for 10 s, 57 ºC for 20 s, 72 ºC for 40 s), final elongation at 72 °C for 30 m, and cooling to 4 ºC. Fluorescence was quantified in a plate reader (Tecan Infinite® M1000 PRO, www.tecan.com). Data were analyzed using TIBCO® Spotfire® Analyst software (www.spotfire.com) to visualize the FAM/HEX signals and make the genotyping calls.

Genotypic characterization of edits to identify gene variants

Digital PCR (dPCR) utilizing hydrolysis probes was conducted using either Bio-Rad's QX200™ Droplet Digital™ System (https://www.bio-rad.com/) or QIAGEN's QIAcuity™ Eight Digital PCR System (https://www.qiagen.com/us). Regardless of platform, dPCR was carried out in accordance with the manufacturer's guidelines for processing without the use of a restriction enzyme. Assays for detection of each edit were run in duplex with a two copy reference gene. The genotype variant assay was run as a triplex with 2 variant probes fluorescently labeled with either FAM or VIC, and reference labeled with Cy5. Maize High Mobility Group (HMG) gene was universally used as the known reference for copy number variance (CNV) calculations. CNV was either calculated by platform software or was calculated with the following formula:

$$CNV = \left(\frac{Reference\ concentration}{Gene\ of\ Interest\ concentration}\right) x\ Reference\ genomic\ copy\ number$$

RT-PCR and sequencing for characterization of edited variants

Quantification was conducted using a Roche LightCycler 480 II (diagnostics.roche.com), employing the PowerUp™ SYBR™ Green Master Mix (Thermo Fisher # A25742) for RT-PCR. Each primer was run in triplicate, and expression levels were quantified as CP values to quantify transcript levels in the samples. Sanger sequencing of the cDNA was outsourced to Eurofins Genomics (Kansas City, USA, eurofinsgenomics.com). Contigs were assembled for each sample using Sequencher software and aligned for visualization in Geneious Prime v2025.0.2 (www.geneious.com).

REFERENCES

1. M. Gowda *et al.*, Genome-wide association and genomic prediction of resistance to maize lethal necrosis disease in tropical maize germplasm. *Theor. Appl. Genet.* **128**, 1957-1968 (2015).
2. M. Murray, W. Thompson, Rapid isolation of high molecular weight plant DNA. *Nucleic Acids Res.* **8**, 4321-4326 (1980).

Supporting Information Appendix

Figures S1-S8, Movie S1

Targeted knockout of a host peroxisomal peptidase confers field resistance to maize lethal necrosis

Jung et al.

dhuggaks@gmail.com

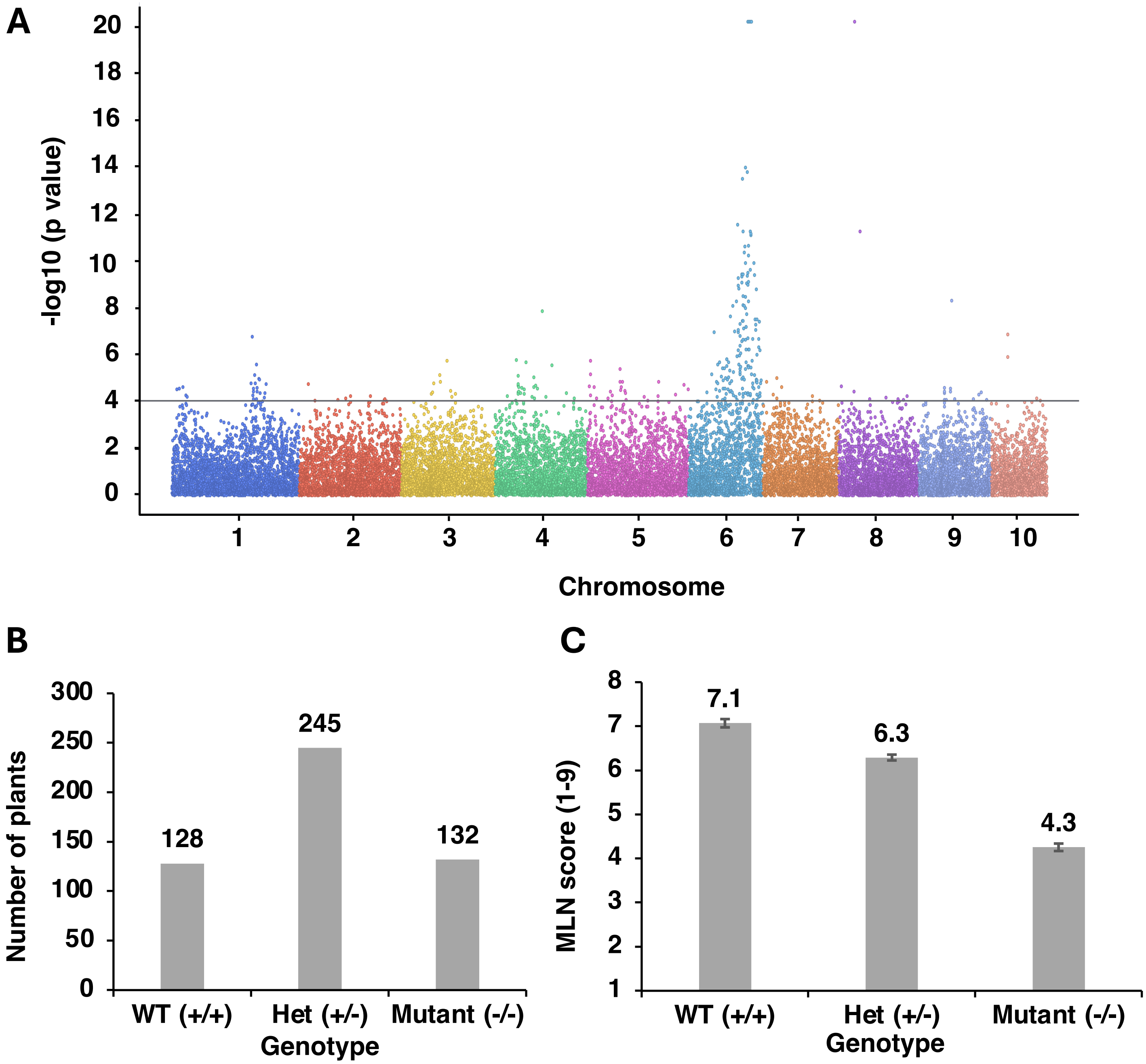

Figure S1. **Signal detection of MLN in maize populations and segregation of the C6QTL locus.** **(***A*) Manhattan plot of marker-trait association results across 8 populations and 437 individuals (Table S1A) for MLN phenotypes from Naivasha, Kenya. The X-axis is the genetic position delimited by each chromosome. The Y-axis is the -log10 of the reported *p*-value, with $p = 0.0001$ as a cutoff, for marker-MLN resistance association. (*B*) Count of genotypes by class across a subset of the F_2 plants that were selfed to generate F_2:F_3 ear-to-row progenies from across the 7 initial populations selected for fine mapping (Table S1B). The genotype, derived from sampling 6–8 random plants in the row, is from either the marker M170 or PZA9758-28, depending on which was polymorphic in the population. Both markers are within 160kb (<0.1cM) of the left border of the fine-mapped interval containing the peptidase gene (Zm00001d038589). The ratio does not deviate from the expected single-factor segregation ($\chi^2 = 0.51$, critical value 13.82 at $p = 0.001$). (*C*) The distribution of MLN5 scores for genotypes in (*B*). Scores were an average of all plants in each row. The slightly lower score of the plants derived from the heterozygous F_2 ears is because one-quarter of the plants in each F_3 row were homozygous recessive, thus were resistant to MLN (e.g., 0.75 x 7.1 + 0.25 x 4.3 = 6.4).

A

Hybrid 1: (CKL05004 x CML543) x CML536

Hybrid 2: (CKL05022 x CML543) x CML536

B

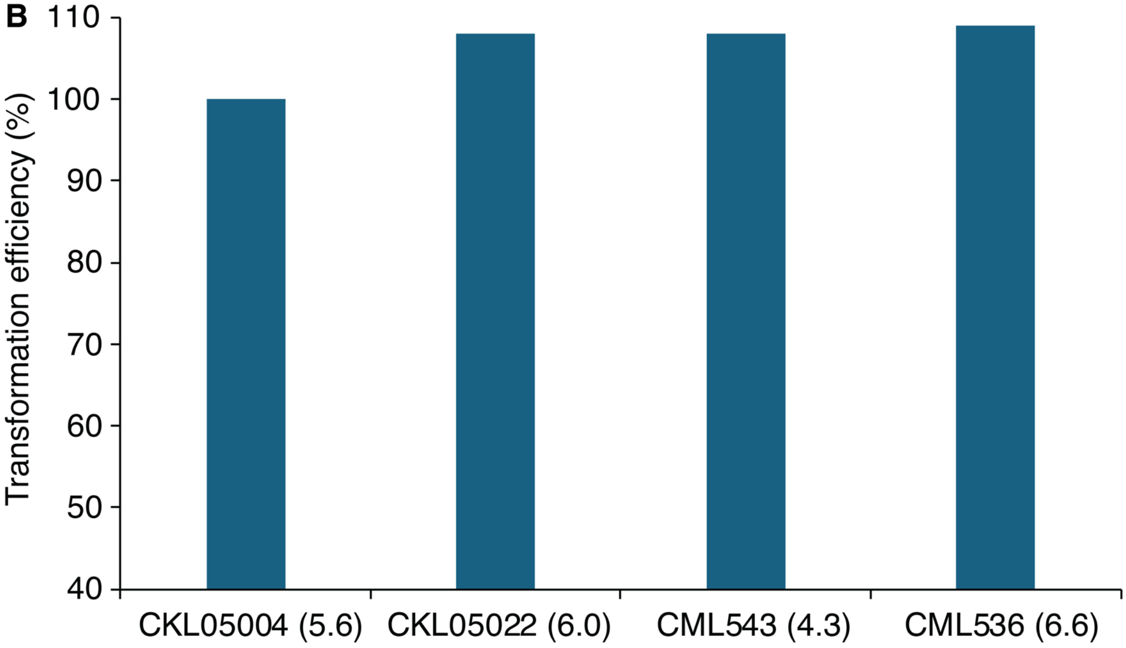

Figure S2. **Commercial three-way cross maize hybrids from Kenya (*A*) and transformation efficiencies of the parents of these hybrids (*B*).** These two heat- and drought-tolerant hybrids were popular in Bomet County, Kenya, before the emergence of MLN. Numbers in parentheses are MLN resistance scores.

A

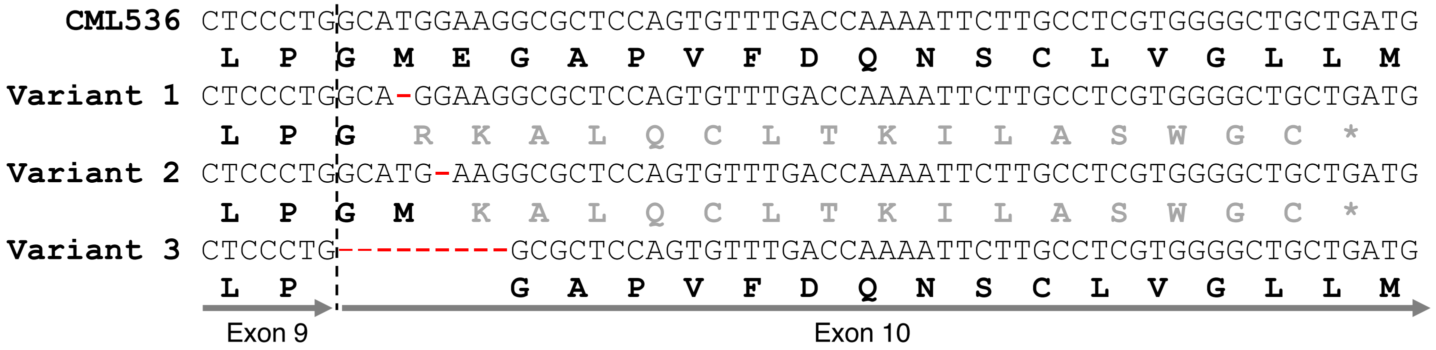

B

```
1   MEAQEIAAAA RHFCAMVRIV GPDPKAVKMR RHAFHFHHSG STTLSASALL LPRGALAEPP
61  PFLDHICSAH GHTAGDVALT AASLVEPFLV AEQRNNSGEE LQPRLVPETR LDVFVEYELG
121 NAQDGKSGPP RWLPARLLAM VDVPTAAVSA LSLLRHDDSF IRRPTWDVGW SLADANQKQV
181 SLFIESKSSL ESNRNNSSLE SVDSLMLAKS ATRIAILGIS TSNLNDARRI NVSVMQQRGD
241 PLLIVGSPFG LMSPFHFFNS VSVGAVANCL PPCTARSSLL MADMHCLPGM EGAPVFDQNS
301 CLVGLLMNPL TQKGSNIEVQ LVITWDAICT EWNSKKLEEI ERPPRKLPND KNTDSKSMEL
361 RHVYNYVRVF SSTDNKTNQH CISPRSLREA ISAVVLVTVG DTSWASGIVL NKRGLVLTNA
421 HLLEPWRFGR TSPSDLQASF AGEHLNAGEN KSLQPQQGKI SNEDAVKHKV SSFNLGFKRG
481 KRISVRLDHE ERQIWCNASV VFISKGPLDV ALLQIEKVPV ELNTIRPEFV CPTAGSPVYV
541 VGHGLFGPRS GLHSSLYSGV VSKVVQIPAN QLSHLARAEA DNMDIPVMLQ TTAAVHPGAS
601 GGVLVNTHGL MVGIITSNAK HGGGSTIPHL NFSIPCKLLV AVFEYSANGN LVVLEQLDKP
661 NEVLSSVWAL APSSSPFVRS SPEKGKEEKV LEFSKFLSDK QQALKSNVDL KELFRYKTPS
721 KI
```

C **Protein Statistics:**

Length: 722 aa (722 codons)

Molecular weight: 78.641 kD

Isoelectric point: 7.73

Charge at pH 7: 4.59

Extinction Coefficient: 60,555

A[280] of 1 mg/ml: 0.77 AU

Figure S3. Transcript sequences of the edited variants for the *peptidase* gene and properties of the maize peptidase protein. (*A*) As anticipated, single-nucleotide deletions in variants 1 and 2 caused frameshift mutations. In contrast, variant 3 exhibited alternative processing of the 10th exon, resulting in a predicted in-frame deletion of nine nucleotides. (*B*) Amino acid sequence of the peptidase. The polymorphic amino acid, G250, is highlighted in red. The three-amino-acid deletion (MEG) in variant 3 is also highlighted in red. The peroxisomal targeting signal, SKI, is highlighted at the C-terminus. (*C*) Biochemical properties of the peptidase protein.

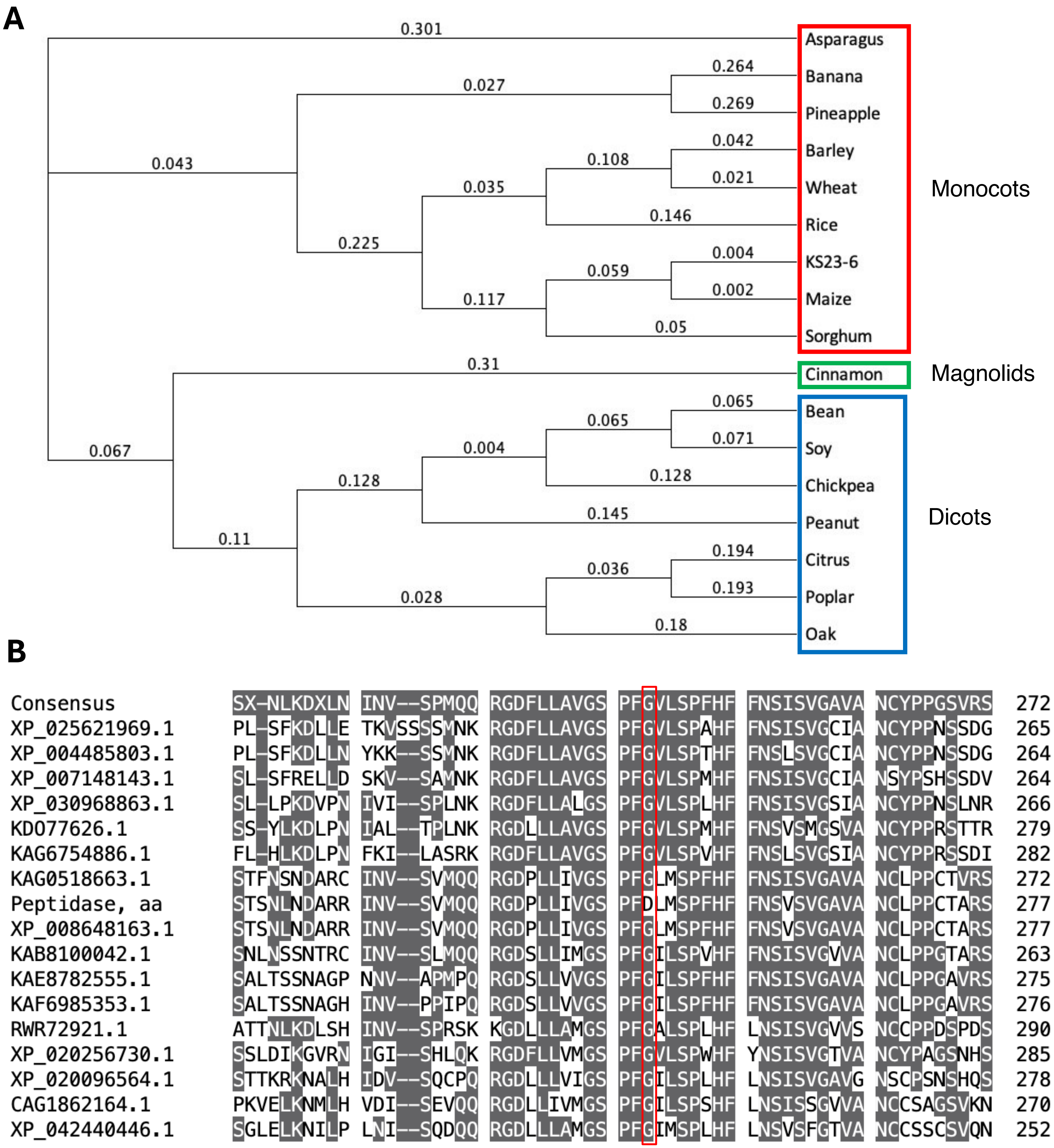

Figure S4. Phylogenetic analysis of peptidases across plant species. (*A*) The peptidase protein is present in all angiosperms we examined. (*B*) The amino acid G (red box) at position 250 that is apparently required for the peptidase to facilitate MLN development is conserved in all the species except in the maize line KS23-6, the resistance donor, where it is replaced by a D.

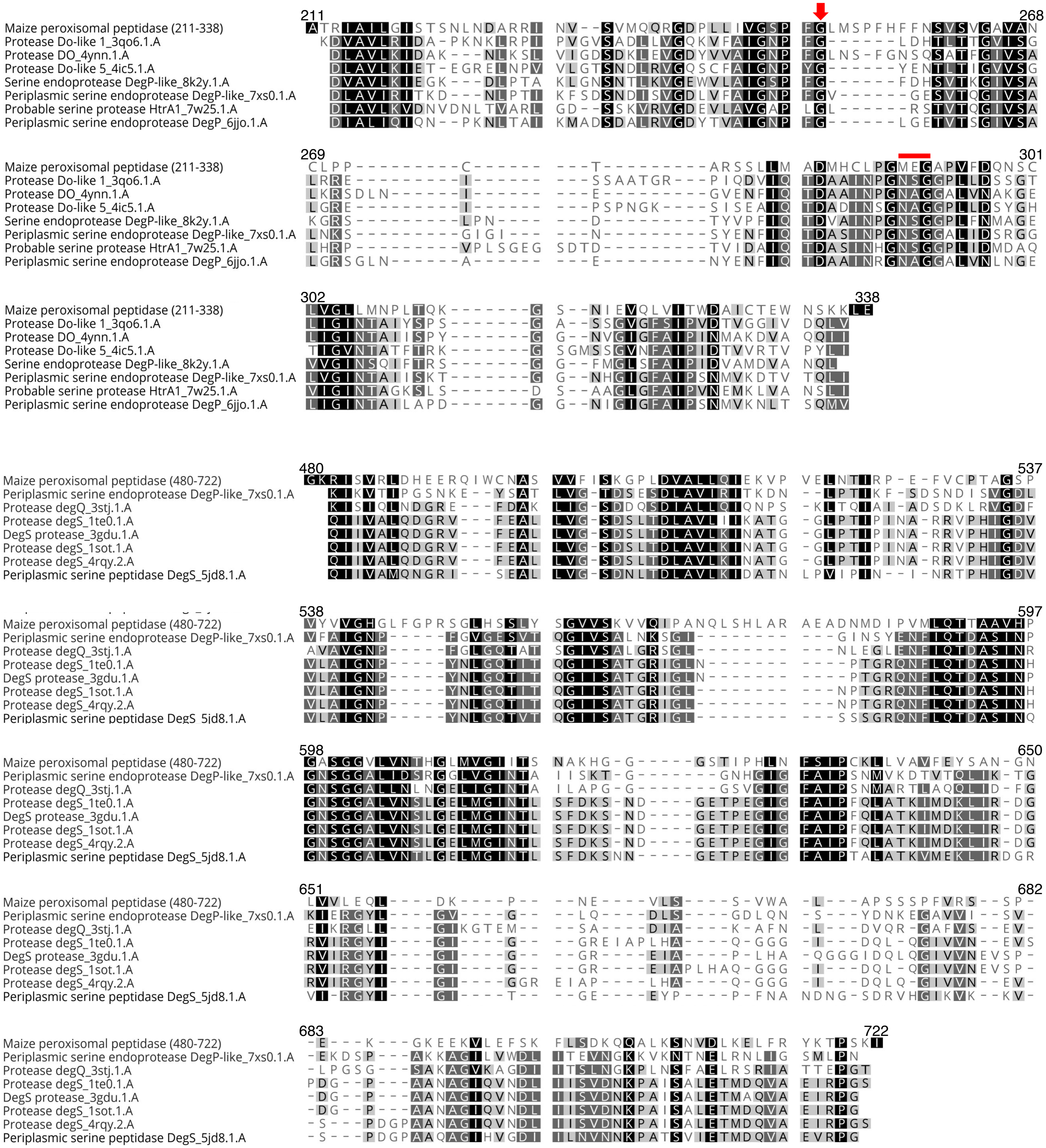

Figure S5. Homology structural modeling using the amino acid sequence of the CML536 peptidase in the SWISS-MODEL server (https://swissmodel.expasy.org). Two distinct domains, spanning amino acids 211–338 and 480–722, exhibit structural similarities to proteases or serine proteases. The amino acid residue associated with MCMV susceptibility, G250 (red down-arrow), is conserved in all the proteases from other species, suggesting that the G250D mutation in KS23-6 may alter the function of the peptidase. The G292 residue of the in-frame, 3-amino acid deletion (highlighted with red bar on top) of one of the edits is also conserved and could explain the resistant phenotype when it is deleted.

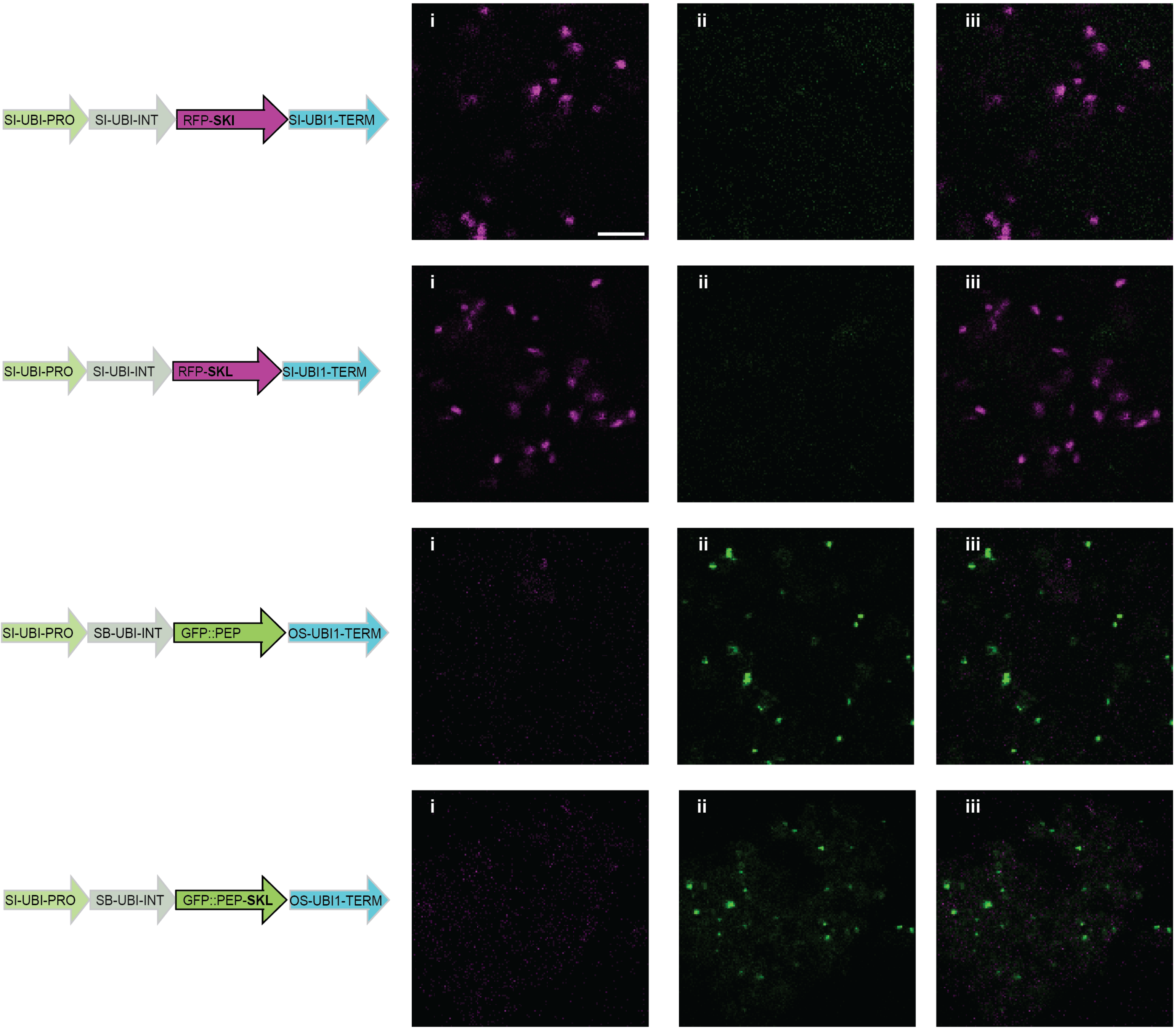

Figure S6. Expression of fluorescent proteins in cells expressing individual green and red fluorescent proteins and GFP::peptidase fusions with either SKI or SKL as the targeting signal in each vector. A sketch of the corresponding vector is to the left of each panel. **i**, red channel scan, **ii**, green channel scan, and **iii**, overlap of **i** and **ii**. Scale bar, 5 μm.

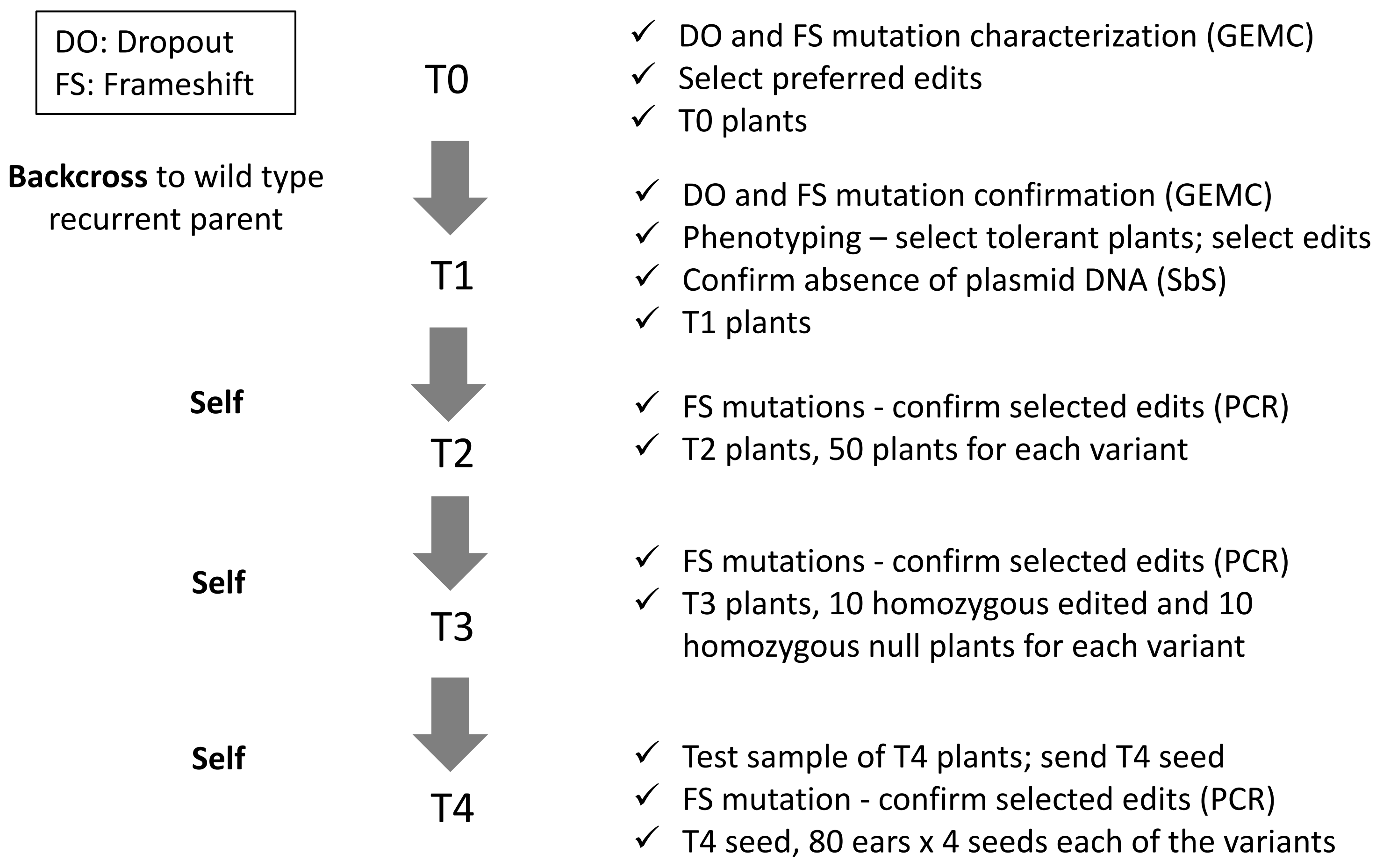

Figure S7. Schematic for pure seed preparation to apply for approval from the National Biosafety Authority (NBA) of Kenya to plant these seeds in open fields as non-GMO. The following assays were used for the edits: Edited locus sequencing, genome edited molecular characterization (GEMC), variant assays, Southern-by-Sequencing (SbS), and marker genotyping for genetic purity (see Materials and Methods for details and protocols).

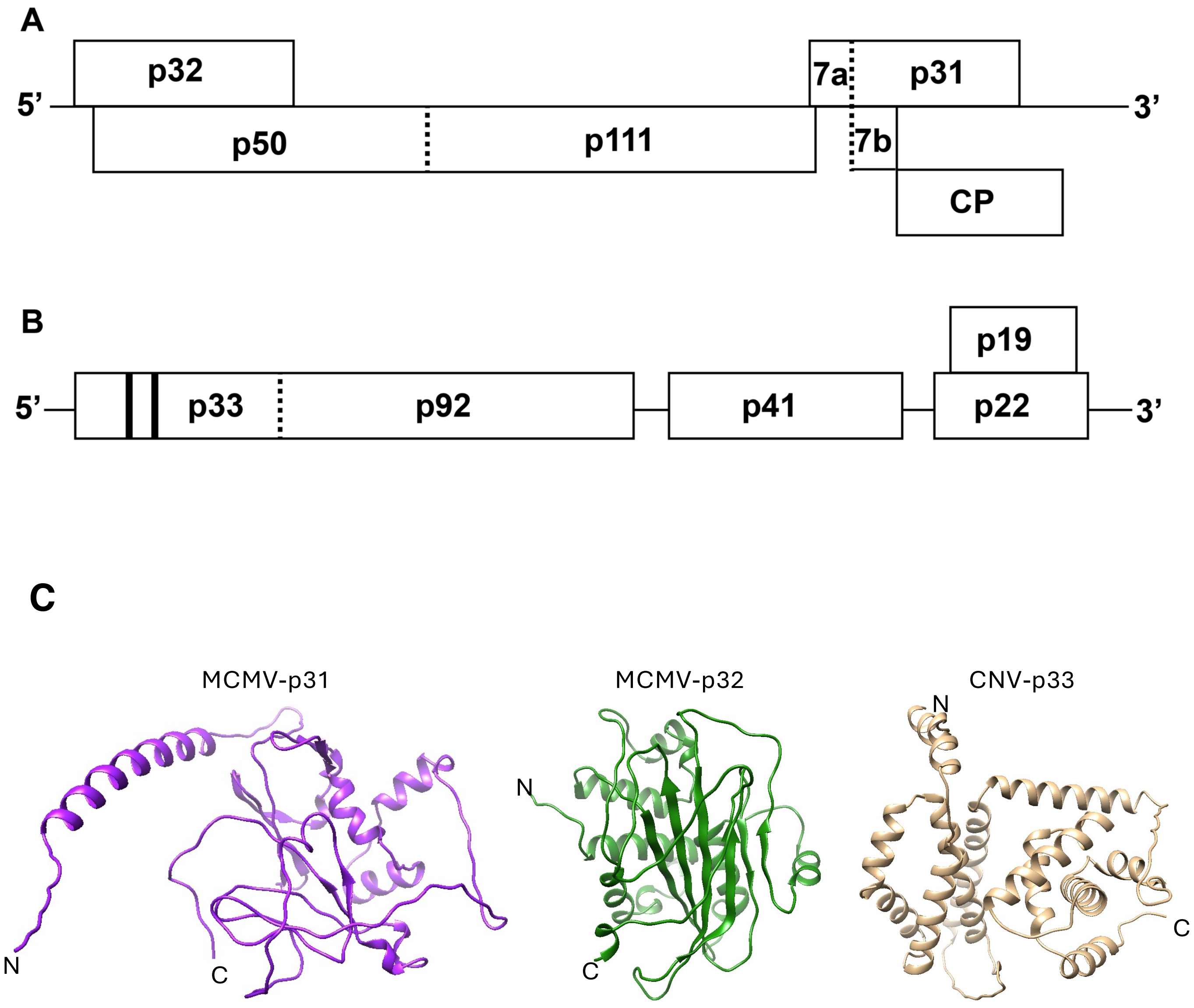

Figure S8. Genomic structure and predicted protein structures of MCMV and tomato bushy stunt virus (TBSV). (*A*) and (*B*) Cartoons of the genomes of MCMV and TBSV (Sheets, 2016; McCartney et al., 2005). Dotted vertical lines point to the readthrough codon for the respective ORF. (*C*) Predicted structures of p31 and p32 from MCMV and p33 from cucumber necrotic virus (CNV). The MCMV p31 structure is composed of *α*-helices loosely connected by loops, suggesting a flexible overall conformation. In contrast, the predicted structure of MCMV p32 features a more organized central domain consisting of *β*-sheets and alpha helices. The CNV p33 structure consists predominantly of *α*-helices. The structural models were predicted using the BiooFold 1.0 server (https://www.hidimension.cn/).

The peptidase protein is shown targeted to a sub-region of the peroxisome lumen.

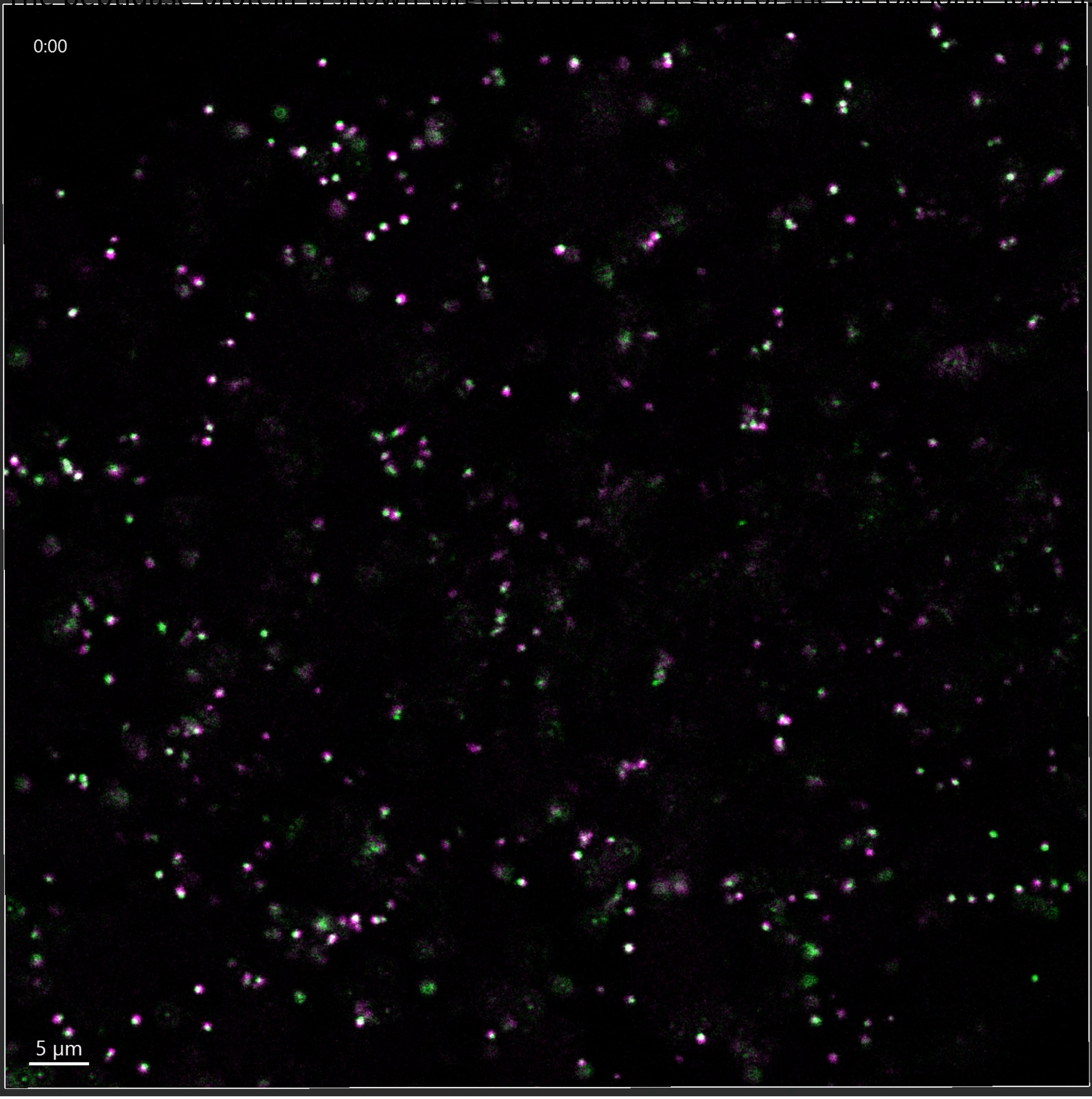

Movie S1. Time-lapse microscopy of subcellular localization and dynamics of peptidase and peroxisomes corresponding to Figs. 5 and S6. The peptidase protein is shown targeted to a sub-region of the peroxisome lumen. (*https://doi.org/10.6084/m9.figshare.28747004.v1*)

Supporting Information Appendix

Tables S1-S9

Targeted knockout of a host peroxisomal peptidase confers field resistance to maize lethal necrosis

Jung et al.

dhuggaks@gmail.com

Tables – Key to page numbers

P. 1: Table S1. Populations developed and screened for response to maize lethal necrosis (MLN) in Naivasha, Kenya. (**A**) Populations with pedigrees and the number of individuals analyzed (N) per population for initial detection of signal for MLN-resistance. (**B**), Initial group of populations (F2 plants or F3 families) used to fine map the C6QTL. (**C**), Additional populations used in fine mapping where N was the number of plants analyzed in each population.

P. 2: Table S2. Markers to test genetic purity of the inbred lines used to generate mapping populations, and the edited variants.

P. 5: Table S3. Markers used for initial mapping of the C6QTL in the segregating populations shown in Table S1.

P. 19: Table S4. Markers used to fine map the C6QTL in populations generated from crossing MLN-susceptible elite maize lines with KS23-5 and KS23-6.

P. 24: Table S5. Genome sequencing of tropical maize inbred lines. Sequence assembly, genome optical mapping assembly and hybrid scaffolding results are shown along with the names of the inbred lines.

P. 27: Table S6. Vectors used to transform maize lines for editing sub-regions of the 105 kb genetic interval. CR2, CR3-4, CR8, and CR13-14 were used initially, then CR8, CR8.1 and CR15 were used to edit additional inbreds.

P. 28: Table S7. Primers for gene-edited events molecular characterization (GEMC).

P. 29: Table S8. PCR primers and assays to distinguish edited variants of four elite inbred lines from sub-Saharan Africa.

P. 31: Table S9. Sequences used to assemble vectors for subcellular localization of peptidase and various fusion proteins.

SI Appendix

Tables

Table S1. Populations developed and screened for response to maize lethal necrosis (MLN) in Naivasha, Kenya. (**A**) Populations with pedigrees and the number of individuals analyzed (N) per population for initial detection of signal for MLN-resistance. (**B**) Initial group of populations (F_2 plants or F_3 families) used to fine map the C6QTL. (**C**) Additional populations used in fine mapping where N was the number of plants analyzed in each population.

A. Initial signal detection

**Pop Name**	**Pedigree**	**N**
Pop1	KS23-5/CZL00025	35
Pop2	KS23-5/CZL00025	28
Pop3	KS23-5/CML545	79
Pop4	KS23-5/CML545	48
Pop5	KS23-6/CZL03018	56
Pop6	KS23-6/CZL03018	52
Pop8	CZL068/CML494	72
Pop12	CML442/(DTPYC9-F46-1-2-1-2-B)	67
All	-	437

B. Initial group (marker screen, fine mapping)

		**Aug-16**	**Nov-16**	**Jun-17**	**Jun-18**	**Apr-19**
**Recurrent parent**	**Donor**	**N**	**N**	**N**	**N**	**N**
CML312	KS23-6	45	52	30	151	
CML442	KS23-6	50	86	99	155	
CML537	KS23-5	42	72	83	193	
CML539	KS23-6	44	74	89	77	
CML548	KS23-6	48	49	69	245	
CKDHL0186	KS23-6	50	64	68	4826	448
CKDHL0221	KS23-6	50	97	98	100	
		329	494	536	5747	448

C. Additional group (fine mapping)

		**Nov-18**
**Recurrent parent**	**Donor**	**N**
CEL08034	KS23-6	53
CKDHL0323	KS23-6	88
CML548	KS23-6	76
CML591	KS23-5	12
CZL1339	KS23-6	123
CZL1346	KS23-6	5
CZL1369	KS23-6	58
TL139555	KS23-6	47
		462

Table S2. Markers to test genetic purity of the inbred lines used to generate mapping populations, and the edited variants.

Marker	Chr	B73 V4 Physical Position	SNP	Sequence
C0031KD-001	1	48,336,941	C/G	CTCCACGGCAAAAGGGCTCTTTCCCTTGTTTGTTGTACACATGCATGTTCTCCAATTTCT[C/G]GAAGAGGGACGAGTCCAAAGCTGGTCTATCCTGATCGACAAAGAGTGGTCCCAGTTCATC
PZA9292-24	1	198,847,827	A/G	CTGTCAAATTWWGTCTGACATTTAACGCACKACTCMACTATGAAGTTCTGANTTTTTTNATGCTTAATTAATGTCGATTGTTGCAGGCTTGAGCGTCCAGGAGGTTTGATTGGATACATG[A/G]CAACGTCGGGTCGAACCTCTAGRGCATCTTTATCATYAGCAGAGCATGCTGTTTATGTAGTTTTGGAYYTTAAGCTATTAGANNNDNNNNNNNTGGATTTGTGGATCTTGAATTTTTGGA
PZA4196-27	2	199,698,626	T/A	ATGCCTCTGCATCTCGGCAGCCTTCATAGCGCTAACCTACGTTGTCGTGGGTCGAGACGASGAGTGGCTGGCCTGGTGTACTATGGCAATTGGCACTGTTACCATGGTGGCCACTGTCGGTTCCATGTGCTACTGCGTTGTCGCTCACAGGATGGAAGAGAAGAGC[T/A]TGAGGAAGATCAGGAGGACCTCCATGAGCCAGTCATGGTCCATATCMATTGACTCGGAAGCAGAGCTGATGAACAGTGAGTACAAGAAGATGTATGCCCTTTAGGATGATGTCYGTKCTTGTCGGCTGAGTCTTGTATATATTGGTACCCATTTGCCTGAGACCAGTCCATGGCATTGAGTGGGATTGACT
PZA10310-4	2	243,174,619	G/C	TGATGTTGCATTTTTTTCAAAATGTTTCAGGTTGGGACTGTGACAGCAATTTGTGTTACCTGCTTCCTGATAAGATGTGTTGTGGTGAGTATGTTGTTCCCACTTTTTACTGATTGGTTGGTGTT[G/C]AAGGAAGGAAGGAAGGTTTAATTATTCCTTTGGTTGRCCGAAAGTAAATGTATGATGTTGACCTGTCTCGTACAAACATAGCACACACAGACAGTAGACTAGTTTGTACCGCACGGGATGCTGCGTGCTGTGCTGTTCTGRGAAGACTGACAAAACGAACGGATTTGTAGTTTGCTAATTATCTTTAAAGAAAAGACACCCCCACATGTAAATGCATATAAATGGTGCAGTGTTTATTTCTTCTGATA
PZA14776-11	3	115,124,885	A/G	GGAATCACTTCGGAATGGCTTCTGTCTTGGGAGAGTTGTTTAYAGYAAACTGAACAACAAGCTGTTRGAAATGGACAAAGTTGAGACCGCATATAATTTGTTCAAGAAAGTCAAACATGC[A/G]CGTGCACTTACGAATTCACRCAATTATTGCCGTGCTAATGGTTGGCATTTTTGAGCATAACCAGTTTGTCTGCAGAAGAGCCATGKAGTCCRTTCSGCATCGGGGARCRCTGTTTACGTG
PZA13478-33	4	19,770,259	C/G	CATCTGGACTGCACCTGTGTTGACCCAGGCCCAAGAATACGAGATCTACGATCTGAAGCC[C/G]TTCTTCTCCAGCGCGTGCTTCAGGGACAACGGCTTCSTTCTKGATGARGGGCGTGGGATC
PZA9253-19	4	244,802,363	T/C	TTTGCTAGTACCTGATATTTGRTATTTTATTAGATGCAGACTCATCTGTGAAAACTGCATCCAAGCCATCTGGCCGCCCCTGGGATAAGATCGCTGCAAATCTGATGGCTTGTCTTATGG[T/C]TATTGTAATGTTGGTTCGATCARTCAAAGATCTTGCCACAATGAGATTGTCATMTAAAGGCGGGTCAGAAGAAAGCYACTGTACATTGTAYCCGGACTCCGTCCAGAAAGAGGAATTCCG

PZA13227-8	5	146,540,589	A/G	atatgatggcttctttggTaAATCCACAGTGTGCCCTTCTTTgAGATcaTCTACAGCCCAGTTGCTGGACGCGCTTCTGCTTGCGAAGTGACAAACCAGTCATGTTCCATATCCGGCCTTGTTTGGTTACATGAGTTGAAATCCCACCATAGATTTAATCTGGATATGGATTGGGTGAATCCATATCTCACACCCAATCTCATGGTGGGATTAAATCCATCAACT*AATCCATGTGTCTCTCAAAGCTAGTTATTCTTTTAATCCATAGATTCTAATGTAAACTTGTGCAATATCTTCATGGATTTGTTCCATACCTAATGAGTTATGGATAGAAACCATGCCTTCCATAGTTTAAAAAATTCCTAAACCTTTGGATTATATTACATGATGGGTTTAACCAAATAAAAAAATT*AAGTAGTCTTGGATTATTTTTGGACATGGATTGTGACATGGATTTAATTCCAATCCATGCTAATCCAGACCTGGATTGGTGTAAACGAATAAAGCCTGATATGGAACCACGACTGGTTTGTCACTTCGCAAGCAGAAGCGCGTCCAGCAACTGGGCTGTAGATGATCTTAAAGAAGGGCACACTGTGGATTTACCAAAGAAGCCATCATATAGAGCATAATTAACAGTAGCAGATTTGATCTCTGCATCAACTTG[A/G]CGTGGATCTTCATCTTCCGAAATGGAGATCTACACAATAAAAGTTAACAGCATTCAAATTGAGCTCCCTCTCTACGTAAATCTCTACAAGCCCCCTTTTTATACTTTTATTTATGCACAAACTTGTTAATTTTTTCC*AGAATGCAATGATCATTCTGTTCCGTAA*CTAACAAAATAAAA*TTTAATGTTAAAA*TATT*CAAAATTTGAA*T*AAATCC*ATCAGTAG*ATAAAAGGA*AATCTGG*CTAA*CAAACAGAATTCCAGCTTTACTGTCAAATCTTTG*ATGC*AATTCAAGCTTCTT*GTCAACCTCAACTAAAGCTTTAA*gg*tctAcaaTTT
PZA9550-19	5	178,968,634	C/G	TCTTCTGCTGTCCCCTAGGTCAGNNNNNNGCTCAGGCAGGCAGCAGCAGGCACGCGGAGGCAGCCTGCCGCCGYCGTCGTCCGAGGTCCGGGACGGCGCGGCGTTCGAGCAGGAGGTGGC[C/G]CAGCTGATGGAGAGCGACATGACGACGGCGATGCAGTACCTGCAGAGCAAGGGCCTGTGCCTCATGCCCGTCGCGCTGGCGTCGGCGATATCGGGCCAGAAGGGGGCGTCCTCCGCGGCG
C00256F-001	6	86,010,625	G/T	CAGGTGCTCTTTCTGCATACTCTGTTCATTTCTGCATCATTGACTGTTGC[G/T]GCTGCGCATATTGATGGACTGTGAGATGTAATCTTATGGTTTGCTGCAGG
PZA7663-17	7	173,378,554	T/G	ACTGGAGAGTGGAGCGATGGCAGAATAACCAATGTATGNGCNTGCCTACTGCGTTAGACACGATGGTATCAAGGCTCTAACAAATACTAACCTCTTGTTACCTTTGTCAACAGGCAGTAGAGCTCTGTATGGATGCTTGCA[T/G]CAAGCTGGGTGACATATTGCGTGACCGCTTGAAAGATACCGCTACCTTGACGTGTGAATGACGTGTTAGAGAGAGCACTGGCAACAACAGATGTTCTTAGCTGTACAAATTCTGTAGGCAGGGGAGGCTGATCGGCCTGATGTTTGAGTGAGCATGATGCATGGTTATGATGATGGTCTCCTTCACGGCAAAAGCTGTCAAACCGGGAAGAAATTGCAGGGCAG
PZA18296-17	8	161,319,804	C/G	tttcggtatACTgtaTctgtcTATTCCTCAAATAAATCATGTCAGTTTTTTTTTGGTTTCCTCCATCATCAATATTAACTCGTAATTTCAGTTCATATATTTACATATAGGTATATTAANCTANGTGGTGTGTAATTGTACAACAACNAGTAGGTTGGCTGGCACCAGTAGCATCNTTTTTTTTTGGATGATGAGAATTCACTCGAAATATGTTGCAATGTACCTTTTCAACTGGATGGAACACTATACTACTACAACCAGCTGCTTCCTTCTTTCTTCTGGCAAAGGCCATTGGACTCATGCCTACTATGGCTGATCAGGTCAGTTACCTTGTATCGTCT[C/G]AAGCGGTTTGTTCGGAACAAATGAGACATCCATCCTTTAATAAAGCGGGCGGGAGAGGAGGCAGATGACAAGACAAGTGCAGTGTGTGTAACCACTACCAGGCGTTCCTTTGATGAATGTCATTTTTCTTCTCTTTAGTCCTGAATAAATTCTGAtaccatatcttttgggtctgggatgcaaaactctgctagcttatcattgcca
PZA10564-6	9	155,131,622	C/G	TTCAAATGCAGCANTTTGTGAGAGTGAGGCACCTGTAGCAGATGAGGACGCAGAGCTTGATGAAACTCAGTCTAGAGCTGCTCTCCCCACACCGCAACATGCTGAAGCTGATCATAC[C/G]GAAGCAGCTGTGGATGAGAATAAACATACAGGAGNAGGGAAGGTTACTGCTACTGCANCGGANGACGATGCAGTAGAAAAGAGTCATGGAGACCCAAGAACCTCAGAGACCTGCAATCCTGCTGGACCTACAGACAAAGCGGAGAAACCTGGAAGGTACTCNGAACAAGGCGCTGNTGATGTGACTGAAGATCGTCCAGAAAAAGAAGCCCAAGCTGTTGGGACCAGCGACACTGGTTTCGTGTTTGA
PZA7977-10	10	56,829,285	G/T	GAGATTTCCACCACAACCGTGTCATCGTCCTTGGGAAGGGGCTGCTTCCA[G/T]GGCTCTCCGTCAACCCTCATGAACGTGTGCTCTGCTGCGCCTTTGTGGAA

PZA686-27	10	124,410,337	A/C	GAGGGAACAAGCGGAGCGGTTTTGGTCGGGAGCTCGGAGAATGGTGGGTGTTGTACGCGACGTCAAAAGATTG[A/C]ACAAATTGTAGCTATTATACTCCCTAAGCAAAGGTTTTGTAAGCTGTATCATCTGAATTGCCTTCACTATTACTTTCAGGGGTCTTGATAACTACCTGACAGTTAAGCAAGTCACCAAGTATTGCTCGGATGAACCGTGGGGATGGTACCAGCCTCCATCTAAGCTGTGAAGGGGCATTCACTCCAGGGAATGTAATGCATCATTAACTGWCAGCATCAGTCCTGTTAATGGACAGGAGCTCGTGTACAGTACATGCTCGTAAAAAAAGCTCTTTGATGGTCAGGGATCATAAAAGTTGCTTG

Table S3. Markers used for initial mapping of the C6QTL in the segregating populations shown in **Table S1**.

Marker	Public Name	Chr	B73 V4 Physical Position	SNP	Sequence
PZA788-18		6	141,206,807	A/G	CCAYGTTGTCCCGGCGAAGGCCGTAGACCCCGGCCTGGTGTACGACCTCG GCGTCCACGACTACGCCGGCTACATCTGCAGGCTCCTCGGCGAGGCGGCC CTGAAAATCATAGCCATAAACASCAACTTRACTTGCGCTGAGCTCGAGCC TGTCACCGGRGCGCAGCTGAATTACCCGGCGATACT[AG]GTGCCGCTGC GAGCTGAGGCGTTCGCCGTGAACCGGACGGTGACGAACGTCGGACCAGMG AGGTCGAACTACACCGCCAAGATCGAAGCGCCGAAAGGCCTGACAGTCAA GGTGGAGCCGGCGGAGCTGGAGTTCACGAAGGTAAATGAGAGGAAGACGT TCACGGTCACAGTGAGCGCC
PZA14535-11		6	143,883,776	C/T	GTATGGGACTATTGACCTCTACTGGATTGTAAGGGATGGAGGATTGATGC TTCTTCTGTCCCAACTCCTGCTGACAAAGGAGAGCTTTGAGAGCTGTAAG ATCCAAGTCTTCTGCATATC[C/T]GAAGAGGATACCGACGCAGAGGAGC TGAAAGCTGATGTCAAAAAGTTCTTGTATGATCTTAGGATGCAAGCTGAG GTCATTGTTGTCACTATGAAATCATGGGAGTCACACATGGAGAGC
PZA236-8		6	144,428,098	C/G	TATATATAGCCGACTGCGGCTAACCATCTGCATATGTTAATTCACTGTYG TTGNNGCTTTAATTACACGAGTAGTGTGGCTACCTGATATGTTCTGAAAT GTTGCTTGCACGTACTGAGTGATTGCCTCTACGTTTGCAGGGCGCAGCCG CGGAGGCCAGACTCGCAGGGA[CG]CAGCTGGGGCAGCAGGGGTACAGCG AGATGGGGAAGAAGGGCGGTCTGAGCACCACGGACGAGTCCGGCGGCGAG CGCGCCGCCAGGGAGGGCGTCARCATCGACGAGTCCAAGTTCACCAAGTA GTCCTATMGNGTGCGTGNNCGTACGCAGCGTTCGTAGCTGTCTGCGTAGT GTACCTTGGCCGTAGCTAGTGCTACTCTYTAC
PZA2555-21		6	145,415,622	C/T	TNGGCTTGCTTCCTGATTACCTGGATAGAGCTGCTGTACTGACTGCACTG CGGAGGAACATACAGGAATCCGGCANCCTAGAGTCCTACTTGAAGCTTTG CAAGCGACTATCTGAGACAGATCTGATTGGACCATGTATTGTGTATCTAT ATGTCCGCAAATTCAAATTGTGGATGGCACACATGCTTTAGAGTTCTGA[C/T]GAACCCTTTCGTCATATATCCACTCATGGCAGGTACAAAATGGAGA TCTCNAAAAAANTGTGNGTGGCTCNAACGCTTGTCCCTGATGAACAANNN GACATAGAANCTTCAATTTNGGTGAACCGGTTGGTACATAATAATGGTTC CTTGTATATT
C00097-01		6	145,728,239	A/G	CCGTGCACTTNGGTTCGCCTGTCTCAGTTACNAGAGGAGNNNNNCCTAAT CCCATNNNNNNCGTCGCTCGNNNNNNNNNNNGATGTCNGCATCAACGACC AGCGNNGCAGCCAGCATGNNNNNNNNNNNNNNNNNNNNATCAGGGGGTGCC GTCTCTATCCTTCNTTCAGCGATTCGCTTTCTGAAAAGAAC[A/G]TTGC ACAAAAACAGCAAGTAATGTTTCACACANATANNNNNNTATATATGCGCT GCTAGNNNNTTTTCATGNATATCGAGANNCTGACATCTGTTTTNGTTTGN TTNTAACAGGATGGCTCTNGGTGCGGATGA
PZA16666-16		6	145,942,253	A/G	TTGTAGCGGCAGCNNNNNNATCTCAGCAATACCAGTCCACTTCGAGAACC CCTACCATGCCACCGAACTTGAGGCCTCTACTACAGCAGTCGCATTCTTT TGGAGTACCRCTTCCAACCGCGAGTCTGGACTCGCCAATAAATGGGAACC TGAAGCAACCGCTGTCTGCTGGGGCTCGCAGGCCATCTTATGTTTCTTCC AACAAGTTCTTCGACGACTTGTTTGAGAGGAACTCGGATGGTAGCCTGAA AGTAGGCAGCACAAGGCAC[A/G]GTGATGTCTATGGAAAATAAATGTCG

					TTCTCTTGTCCTGCYTGCGTTTGATGTTATCAGCTGCCTGCRTATTAGAA CGATTNNNNNGATGCTTCTGGCSAACTTGCTKGCCTTTYCATAAA
PZA8813 -5		6	146,679,100	G/C	TGCCCGAGGAACAAGTTGATCATCTCCAGGAGGAGAAGAACACCTGAAGA GGAGGAATACTTGCGAGGTTTCAATCCACGGGCACCGAATTTTCTTCCTC TGGAACTGGATCCAGACGCAGAGAAGGTTGACCTCAAGCATCAAATGATG GGTGAGCGGAAGAATGCTGAAGAATGGATGATTGATTATGCGCTTAGGAG GGCGGTGACAAACTTAGCTCCTGCTCGGAAGAAGAAAGTGGAGCTCCTTG TCCAGGCTTTCGAGACTGTCCTACCACATGACGAAGATGACAAGAAAAAT GTCTCACCCTCAAGGCCTGTCCAAGCCTGCAACTGATAACATTAGAGGCA AGCGTTTCTTTCTTTGAAATGTTGTTCCTGGTATTAGTTTTGTCATTTCA ACAAATTCTTTACACTAACTACTCTTTTGCTTTTTTGTGAATATTTTGCA GTTAGTGGTTACACTGATGTTGATGTCGACGCGCAAGCTACTAACTCGGA GGTTGCTGCAATATTATCGC[G/C]AGTGACCTGCTCCAAGTTTGAATGA GAGAGCGCTACTTACTAATTTGCTAGAGGTAACTAGAGAATTAGAGATCT CTAGCTGAGCTGTGACAGAGGAGCTCACTCCTGCTCCACAAGGAAGGATA ACTACTCATTAGTCATTACTGATCAAGGTATCCCTATCCAGTTGCTGAGT TGCAAAGAGGATGCTCCCGGCTTGAGTTATGTAATTTAAAAGTGTGTATA CATGTCTCGTGTGCTAATGTTGTCTGATATTGTGCTGGTGGATCTGTGAG GATTGGAAAATGTCAATGGTGATGAGGTCCATGCTCCTTTGGCAAAGATC AATAAAACTCTTCTTGAGTGCTCAATTGTTGTTACTGAGGTTGAATCTGC GTCACAATAGCACCCATGCACACATGTTTATATTTGTAAATCCTGAGAGC CATGTGCCTTTCTAGGTGCACACGCTATGAGGTTTCGTGAACACAGTCCA GTACCAGTTACTGAATCGTCTGAAAATCATCAGGTCGATGTAGCCGAAGG TGAACACAGTAACCGATCTGACGGCTGCAGCTTAGCTGTCTCAGTTGTAG AACAGTATATGCCGGTGCTCACTGC
PZA8813 -10		6	146,679,170	A/G	GCCTGCAACTGATAACATTAGAGGCAAGCRTTTCTTTNNNNGAAATGTTG TTCCTGGTATTAGTTTTGTCATTTCAACAAATTCTTTACACTAACTACTC TTTTGCTTTTTTNGTGAATATTTTGCAGTTAGTGGTTACACTGATGTTGA TGTCGACGCGCAAGCTACTAACTCGGAGGTTGCTGCAATATTATCGCSAG TGACCTGCTCCAAGTTTGAATGAGAGAGCGCTACTTAMTARTTTGCTAGA GGTAACTAGAGAATTAG[A/G]GATCTCTAGCTGAGCTGTGACAGAGGAG CTCACTCCTGCTCCACAAGGAAGGATAACTACTCATTAGTCATTACTGAT CAAGGTWTCCCTATCCAGTTGCTGAGTTGCAAAGAGGATGC
C19784-001		6	146,935,010	C/T	GGGCAAATTTATATAGGTCTAGTGGGTACCCGGCTACGGATAGATATGAT GCTGCACTGCACATTKGCTATATCTGAGGCTCCTGCGCGCGCCTTGGCCC TTGGCCAGGTGTCTGTCATGCGGGCGATGCCGCAGGAAGARGAAGCCGCG GTGGCGACGACGACCATGGCCGGGGGCAAGGTGGYGGCGCTGCTGGCCAC GGCGGCCGCGCTGCTGCTGCTGCTCCCGCTGGCGCTGCCGCCGCTGCCGC CGCCGCCCACGCAGCTGTTGTTCGTCCCCGTGGTCTTGCTGCTCCTCGTG GCGTCCCTCGCGTTCTGCCCCGCCGCGACC[C/T]CCTCGCCGTCGCCGA TGCATGCCGCCGACCACGGGTCGTTCGGGACCACTGGATCACCGCACCTA TGTTGACGCTGACGATGAGAGRTCTCGTCGATCGACGGTCGGCTTGCAAT GGCGCGTCGGAATCAAGCAGTTCGCAGTTCGCACACGCATGCATATGCCT GACGACGAYGCCATGGATCGGAGCRAGGAAAAAAAATGTACAGTACAGGG TAAACTTGCTAGTAGAGTAATTGGATTGCTTAGGCGCTTATAGCTTAGCT GCGGCCTTGTAGCG

C19787-001		6	146,935,200	A/G	GGGCAAATTTATATAGGTCTAGTGGGTACCCGGCTACGGATAGATATGATGCTGCACTGCACATTKGCTATATCTGAGGCTCCTGCGCGCGCCTTGGCCCTTGGCCAGGTGTCTGTCATGCGGGCGATGCCGCAGGAAGARGAAGCCGCGGTGGCGACGACGACCATGGCCGGGGGCAAGGTGGYGGCGCTGCTGGCCACGGCGGCCGCGCTGCTGCTGCTGCTCCCGCTGGCGCTGCCGCCGCTGCCGCCGCCGCCCACGCAGCTGTTGTTCGTCCCCGTGGTCTTGCTGCTCCTCGTGGCGTCCCTCGCGTTCTGCCCCGCCGCGACCYCCTCGCCGTCGCCGATGCATGCCGCCGACCACGGGTCGTTCGGGACCACTGGATCACCGCACCTATGTTGACGCTGACGATGAGAGRTCTCGTCGATCGACGGTCGGCTTGCAATGGCGCGTCGGAATCAAGCAGTTCGCAGTTCGCACACGCATGCATATGCCTGACGACGAYGCCATGGATCGGAGC[G/A]AGGAAAAAAAWTGTACAGTACAGGGTAAACTTGCTAGTAGAGTAATTGGATTGCTTAGGCGCTTATAGCTTAGCTGCGGCCTTGTAGCG
PZA8022-5		6	147,385,258	C/G	AAAATTCATCACAAAAATCATGTATATAGTACACCTAAAGTTAAGTGTGGCCACAATAATTCAATTTAAAATTCATCTTTGTTGTTGAGATTAAAAAACAGAAGTGCCACTGTCAGCACATGATGAGCAGGCCCAGGTTGACTGCTTGTCTATTTTGTTTCTTGACATCTAGGATGAATTTCAAACTGAATTGTTGCGGCTAAACTTTATGTTTATTACATGCATGATTTTCATGATGATTTTTAGAATATTGCAACATGATATTTTAGCTAACGAGGGCACCAAATCAGCACTCATTAGAATAGTTATCAACTACTGCACTACAAAATGCTTTCATATCTCTCCGCTTCTTGACTAGCATCATTTCATGGAATAATATTAATGTCATTTACATACAGATAAATGCTTAAGCATAAGCTATGGAATTTTGTTTTTCTTGAACATGTAGGAGAGTAACATTTCATTAATATAAAAAAATACAAACGAGGTAGAGAATGGTAATAAAAACACAAACCAAGACCACCACCACTCACATTTGTCCTGGCAACTTGATTTCAGTAGGGACCTCGTTAGGAAATTCCCATGCTTGCAGTGGGTGTA[C/G]GATACTATCAAACTGATAGGCACAAAGTGCTCCCCATTTTAAGATTAGTAAGAACTTTGTAGATACCCGTTGACTTATGCACGAGAATGACTACTAACTATTATGTCATTGATTCTTGATTTTATTTGTATTATATTTCTAGTTTGCTATTTACAGTTAATAAAGCTTTGGACATTTCAATCGATGAACAACAGGTCAAAGACAGCATCCCGTGCGTCAACTAGATTCTTTTTGTCAAGCTTTTGATGTCGCAACTTTGTGAGCAATACTCCTTGTTTATCCATACTTCATAGGACATGAATAGAAGGTATGACAAGTGCAAGCATAGTTATGTAATATACAGTGGCTAGTTGCCAGAAAATGAGATTTAGTTGTGTAGAGCTGTTTGTACATATTGAGATGGTTGTTTCAGTTCAATCTCAACAGGTTTGAGGAAAATATCCAACGAAATGATACAGTTTTAATGCTAAATTAGTTATTTTGTACAGATGATTCCAATGCTCGCATGATTCTATGGCAAACATCCTTTGCTATAAAACCATAGCTCGTAATTTTGTAATGGGACATAGCAGTTTTGATTAGCTTAATTACAAGTGTAGA
PZA8022-7		6	147,385,366	C/A	AAANNTACAAACGAGGTAGAGAWTGGTAATAAAAACACAAACCAAGACCACCACCACTCACATTTGTCCTGGCAACTTGATTTCAGTAGGGACCTCGTTAGGAAATTCCCATGCTTGCAGTGGGTGTASGATACTATCAAACTGATAGKCACAAAGTGCTCCCCATTTTAAGATTAGTAAGAACTTTGTAGATACCCGTTGACTTATGCACGAGAATGACTACTAACTATTATGTC[CA]TTGATTCTTGATTTTATTTGTATTATATTTCTAGTTTGCTATTTACAGTTAATAAAGCTTTGGAYATTTCAATCRATGAACAACAGGTCAAAGRCAGCATCCCGTGCGTCAACTAGATTCTTTTTGTCMAGCTTTTG

C00308-01		6	147,780,305	A/G	CAAAAAAACTGAAGGACAAATCAAAATGGTTACACATCAACCGTCCCATTTCATTGTTAA[A/G]ATATTTGCTAAAGAACGATGCACAGTTATCAATTACTGAATTACCCGCCAGCTGAGCAAA
PZA12799-5		6	148,081,071	C/T	CGCGAGCAAAGGCGAGGTTGTAGGTGTGGATGCAAATGGAAAGCTGAGGATAAAGTTTCGGTGGAGGGATAGACTTTGGATTGGGGACCCTGCTGATATTGTTCTTGATGATGTCCACTTATTGACGGAGGCTTCTAATGGCTTGGCTTTTTGTTCATAGGCTTGTGGTATTCTTT[C/T]GATACCATATCTGATATATTGCACCCATCCATTTTGCAGCATTTCAAATTTAGCAAAATCTTAACCTGCAGTGGACTTGCAAGTTGCACATAACTATCATCGCATCGGCATGCCAGGGTGTATTTCATGTTGATAACCGGGGAATGAGAAGACAACGATAGTGTAGAGCTGGTAACCATCACCTAGTTTTTTCTTCTT
PZA3962-4		6	148,945,814	T/C	ACCAAACATTTACATGACGTCATTTAGTGTTCATCGCCTGCTTATTACAAGTGTTGTTGTTGCTGCCAAATTCATAGATGATGCGTAAGGACCCACCATAACAGCTTTAAGTTACATTTG[T/C]GCATATGTACTTTGTATGGAAGTATGATCTTACATACTGTTGGCCATAGACTGATATCAGGTCTTCTGTGTGATACTGATCATAGATGCAAACTCAAACTTAGCTGAGTCAATAAACTAA
PZA1204-19		6	150,034,674	C/G	TTGGTNTTKGTGTGCAAACAGGTGGGCATTTGTGGAGAACACGGTGGAGAGCCTTCSTCTGTGGCCTTCTTCGCGAAGGCTGGGCTGGATTACGTTTCTTGCTCCCCTTTCAGGTCGGTT[C/G]AGTCACTGATAAACTCGTGATYGAATNNCCAATAAGCGTATCCTCTTATGTTAACGGTAGCAAAAATGTTCACTGTTTTCTTTGA
PZA2362-22		6	150,513,394	T/C	TCTCTTCTTCACTGCATTCTTYTCATCACAAGAGCTGGACTTCCTCTGATAGGACCACCGAAGCCAATGGGTCTCTAGATAAGCACACCCTTGCTGAACCTGCAAGCCCAGCGCGCAAAGATCTGRATGGCGACGAGTGCACTGTGCAGGTGTCAGATGGCAACGACACTGG[TC]AAAGGCGTTTTGAAGCGTGTGGATTCCAAGGACGGCGATGGATGGGAGCTTTGCTGAAGGTGTTCAGGAATAGCGCGGTTGCGAGTGGATGCTGAAGCATAGAAATTACTATAAACTAGTAATAATGGTGAATAAACGTTTACTAGTGCGTTGCTAATCTAGTCGTAATGTGKGAACTGTC

PZA18967-7		6	151,255,898	A/G	ATAACCAGTTGAGTGGTGACATTCCAGGGCAGTTCAGCGCCCTTGCTCGGTTACAGGAGTTCAATGTTGCTGACAACCAGCTGTCAGGGACTATCCCAAGCTCTCTACAGAAATTCCCGGCATCAAACTTCGCTGGTAATGACGGACTGTGTGGGCCTCCATTAGGTGAATGCCAAGCTTCAGCGAAGAGCAAGAGCACTGCATCGATCATCGGGGCTGTTGTTGGCGTGGTAGTCGTCGTCATTATCGGTGCAATAGTTGTGTTCTTTTGTCTGCGGAGGGTACCGGCCAAGAAGGCGGCAAAGGATGAGGATGATAATAAGTGGGCGAAGAGTATCAAAGGAACAAAAACTATCAAGGCAAGTTGTTAACTTATCATTTGAATTTTTATTTTCTTTTTTTCTAAGTAACCTTTCTCACAAAAAGTAACCAGGAACTTCTCGGTGACATAATGATCATTTCTATTATTATCCAGGTATCTATGTTTGAGAATCCAGTTTCAAAGATGAAACTGAGTGATCTCATGAAAGCCACAGACGAGTTCAGTAAAGAGAACATCATTGGTACTGGGAGGACAGGGACTATGTACAGGGCGGTGCT[AG]CCTGACGGCTCCTTCCTAGCCGTGAAAAGGCTACAGGACTCGCAACATTCCGAATCACAGTTCGCATCGGAGATGAAGACACTTGGCCAGGTGAGGCACCGGAACTTGGTTCCGCTCTTGGGATTCTGCGTCGCCAAGAAGGAGAGGTTGCTGGTGTACAAGCACATGCCCTTGGGCTCGCTCTACGACCAGCTAAACAAAGAGGAAGGTAGCAAGATGGACTGGGCGCTGAGGCTACGGATCGGCATCGGTGCAGCGAAAGGGCTTGCGTATCTCCACCATACCTGCAACCCTCGAGTTCTCCACCGCAACATCAGCTCCAAGTGCATCCTCCTGGACGAGGACTACGAACCAAAGATATCGGACTTCGGTCTCGCTAGGCTCATGAACCCAATAGACACCCATCTCAGCACCTTTGTCAACGGGGAGTTCGGAGACCTCGGCTACGTGGCGCCGGAGTACGCGCGTACTCTGGTGGCTACTCCCAAGGGCGATGTCTACAGCTTTGGCGTGGTTCTCCTTGAGCTCGTCACCGGTGAGAGGCCCACCCACGTCTCCTCAGCCCCGGAGAATTTCAGAGGAAGCCTAGTGGAATGGATC
PZA11985-27		6	151,269,888	A/G	CAGATTTCAGCAAGATTTTTAAGTTCAAGTTTGTCCATTTGGGCGGAGATGAAGTAAACACAAGTAAG-CTTGCAGAAATACGTRCTGTACAACTKWTATATGCATGCTAGAATCTATTCATTCTTTATTATGATGTCAGGTTGCTGGAGTAYCA-MACMA-S[A/G]CATTAAGTCGTGGTAAGAAATTATCTTGTAATATGGCAAATAAATAAAAGGAAATTTTGCTAAYCTGATATGACAGTTGAKAAYCTTTCTGTTCGTTGTAAT-K-TACAARCAACAAACTGYYCTG-CTCAGTCATC-AKYYK-A-TC-DR---MNDNNSCKADWWWAC
PZA4856-36		6	151,716,358	G/A	CTCACAGGGATGGATAATGTCTGAATCACTTCTTAACCCGGGACGAGCAAGATTGTGTGGAGCNGTACCACAATGATCCANGCCTTTTTCGNTTTTCATGATGAAGAGGATGAGTTCTTTTTCATACAATTGGTATGTGAGTCAGCGAAACTAACTTNAAGTCNACAATCAGCTCAAGTACATAAAATGCACCAAATTGTCATGAGTNCTGATCCTGNAGGAACAGTACNTGAANCTGGTTAGTTCTCGCACTAGAACACAGTTTCTACTATTTTACTAANATGTGCAGTAAATCTTATCGGCANGATTTCCTGTATGCAGGAGCGTTAG[G/A]TCGCAACAACCACCATGACAGGGGGGTGCTGACTCAGGAAGGGTCCGT

PZA18354-15		6	153,563,186	G/A	ATCAGTTGTAACTTATAGTGTTCCTGAAAACTTGGTTGCTGCAAATGAAAGGTCCAGGGATAACTCTTTAATACCATCGGTATCCATTTTGCATATGATTCATAACACACTGCTAGTTATTTCTTTTGACTAAGGACATGTTTGGATACCAAGGGCTGATTGTTAGGCCGTGGCTGATCCAAACAGGCCCCAAGACAACATGGAACTGTAGAATGCCATTTTATGTGCTTAATGTTCTTTTACTGTCAAATGTTTAGCGAACTCTTTAGTTTGTACTTTGATCAGCAAAAAGTTGTAGAAATCTGTTGGAGATCGAACTGGAATCTTGTTTTTGAACAAAGACGAACTGGAGTCTGACCAGAATAAATTTTCATTCAACCTGTTCTGTTCCCATTTATCTGAGATGATTTCCTGCTTGCTGCAGGCCTGCTGGTATCACATGTGAAATGACCGAATCCCGTTAATAGTTTTGGTTCACGTTGTGTTATATATTACCACAGTCCACGCTGTACTCATAGTGATCACGCTTGTGCTGTGTCATGGATTCTGGAGGACCATCATTGTTTCAAAGCATTTCAATGTTGTGCTCCAATGTGCCAT[GA]CCCTACAGATGGTAGTTCTGGTTGCTTGCTTGTCAGTGGCATGATCTGTCATAAAAAATATTATTCACTAATCCCTGTATTTATTCAGCACCAACACATGATATAAAAGTTTTTGCTCATGCATCATATATAACTCAGAATTGGCAGGTCAACGAGAGTGGAATTTCTGGGGTGCGGTTACCTAGTGTTAATATATTGTTATGAAAGTGAAGGTCGAATTGAATAGAAATGATCCATTGTATTGAACCTATAGTTTGTAACTGCCCTTACCTGGGATATTGTATCAAATACGGTTTCCTGCATGGACCTGGAACTCTCTCCAATTGCTGCCATTTCGTTTGACTTATCAGATTCATTGAGCAAGGACGCCCTTCCAGGGTTCGTTCTCATCTGCTTGCTGTGCTGGTGCGTCCTTTTGGTGCCTGTCAGTTAAGTTCCCAATCATGCATGCATACAGCAACAGGTACAAGGAGAACAAGTGCAAGTTTTTATGTACCAGGGAATCCAGGATGAACTTTTCTTTGTCGACCTTTTTTTTGGGGGGGGGGGGGGGAACACTCTTTTGATCGCCTGATTGAGGCAAGAATTTCTATATATTTTT
PZA2122-36		6	153,937,150	T/C	TGAACTAAACATGCATCTTTTTGCTAGATAATATGGACAGATAGTATAAATAATGCTTATTGGTTACAGAAATTCCATGCATGTTTCAGCTAATGATAAATTACTGGGACATGAATAGGTTCCTTGTCGAACAGCTATGAGGGGTAGTTTCCCACTTAATGGCACTTACTTTCAAGTCAATGAGGTAAACAGAGTGACATTTAATGTCTTACTCCTAGTACTGCTCCTAAGCCTCCTGTGTCAATGGATACACTTTTTGTGACGTGTGGCTGTCCATACAGGTATTTGCTGACCACAGATCTAGCCACAACCCAATCCATGTGGAAAGGGAGATGCTATGGAACTTGCAAAGGCGCATGGTCTTTTTCGGGACTTCAGTACCCACCATATTCAAAGGTTGTCTCCCTCATAAACAATTCATTGAAATTATATTTGTTCTGGTACCAACTACAGTTGTTTTGATTATGTAGGTCTAAGAACAGAAGAAATACAACAATGCTTCTGGAGGGGTATGAAAAAAATAATTCCATTTGTTCTGTCATGACATTCTATTCCTAGGAAACGTCTTCTTGCCAATTGTCATCAGACAGTTCTGCATAA[TC]GCAGATCCTGTGATAGACCAGTATTCTGGACCATAGTATATTCAATCTCAGCTTACAAGATTCTCATGATTATCACACAGTGACTATTCAGTGATGAAGAAAATCGTTCAGTCACTACATGATTGATCTGTAGTCATCAAAGTTTTTAAAATAACCTGAGAATAGTATAGGCATCTTGTAATAGTGGAACAATTAATGCTAGAGACCAAACTTCCAGTATGGATGCAGAGACAAAGGAATTGATGTGCATGTTTATTCATAAGCTTACATTTCAGACACACAAAGGTCATGAACGGAAGCAGTCTCTAGGTTTCAACTCCACAATGAATTTCTGAACGAAACAAGTAAACTGTGGCTGTCTTACGCAGGATTTGTCTGTGTGCGAGGATTCGACATGGAGACTAGAGCACCAAGGCCTCTGTGCCCCCATTTGCACATTATAGCAAGGCCGAAGGCCCGCAAGACAGCAGCAACTGAGCAAGTACTCTAATCAGCAAAGATATCAGTTTTATGAACATCATGTTTATTGTGATATGACATCAATAG

					CTCTCGACCTATGCATGGAATCATATTACTTCCTGCCTGGCGATGGCCATTGTT
PZA2122-26		6	153,937,314	G/T	GGTCTTTTTCGGGACTTCAGTACCCACCATATTCAAAGGTTGTCTCCCTCATAAACAATTCATTGAAATTATATTTGT[G/T]CTGGTACCAACTACAGTTGTTTTGRTTATGTAGGTYTAAGAACAGAAGAAATACAACAATGCTTCTGGAGGGGTATGAAAAAANTAATTCCMTTTGTTCTGTCATGACATTCTATTCCTA
C0032KY-001		6	154,807,103	T/C	TGCAGGATGTCTTATCAACATCTGCACAAGAATCGCCGCCACATCAGCAAAATCGTATGA[T/C]CTTTCTCCAATATCATCACATATAGCNAGCTTGATTGCAGATGTCTGGTCACTAATTCGC
C0032KR-001		6	154,809,398	G/T	GCGGGGGATTAGCCGAAACGGGTGGCCGGGTGGGCTCACACTCCCACAGCGTTAACGGGT[G/T]CCGGGGGATTAGCCCTTCGTCAGCTTCTTGTGTTGCGTGTGGTTGAGATCCTCGTTTATT
PZA15770-15		6	155,639,013	T/C	GAATATGCATATGGAGCTGCAGGCTCRCCACCAGAGATACCTCCTAAGTGAGTTCCTCTACCACCTATCTTCGAAGTGGTAACGAGTTTCCCCAATTTAACAATGTCGKTANTTCTTTGTGTATGTAGTGCAACCCTTATTTTTGAGGTGGAGCTAGTAGCTTGCAGGCCGAGGAAAGGTTCAAGCTTAGG[TC]AGTGTATCTGATGAGAAAGCCAGACTTGAGTAAGACATCTCCTATCATTCCATCTTTAACATANTAGCACAGAAAAAAWNNCAGTTATTATATGATTTAGCACAACTGTACATGTTCCCTCGAGTGAACTCTTGWTGACAATGCAATATTCAGAGATAGAAATCACAACGKTGTATGCAC
PZA15251-5		6	157,149,203	A/T	TAGCTATGGGAGTACTGTACTGCCTGCAGTATATGCACCAGCAGAACACTCCTGTAACCCTAAAGAATCTAAACAGTTCATACATATACTTGACTGAGGACGATGCCGCAAAGGTTTCAGACATCAGCTTCAGCGTTGACAAGAGAGAAGATGATGAGTAYGATGCTCCTGATGAGTATAGCACAGTGTATAAGTTTGCTTTGCTACTGCTTGAGACCATCTCCGGAAGGCGTCCATATTCTGAAGACGATGGCCTCCTTGTTCT[A/T]TGGGCACGCAGATATCTCACCTGCGCTAGCCCGGTGATGGGTATGRTTGATCCAACGCTAAATTCAGTCCCCGAGGAGCATGCCAGAGCATTATCAGAGCTGATTCGGTTGTGCCTAAGTGAGGACCGAA
PZA15251-3		6	157,149,308	T/C	TAGCTATGGGAGTACTGTACTGCCTGCAGTATATGCACCAGCAGAACACTCCTGTAACCCTAAAGAATCTAAACAGTTCATACATATACTTGACTGAGGACGATGCCGCAAAGGTTTCAGACATCAGCTTCAGCGTTGACAAGAGAGAAGATGATGAGTA[T/C]GATGCTCCTGATGAGTATAGCACAGTGTATAAGTTTGCTTTGCTACTGCTTGAGACCATCTCCGGAAGGCGTCCATATTCTGAAGACGATGGCCTCCTTGTTCTNTGGGCACGCAGATATCTCACCTGCGCTAGCCCGGTGATGGGTATGNTTGATCCAACGCTAAATTCAGTCCCCGAGGAGCATGCCAGAGCATTATCAGAGCTGATTCGGTTGTGCCTAAGTGAGGACCGAA
C001UNF-001	PZE-106102646	6	158,825,991	C/T	TCTGCTTTCCAGTTTGCAGCTTATTTCAAACACATGGCCAAACACCCAAA[C/T]GTGCCAAAAAGAGAGGTTTTAACAGCTTAGAGTACTTCTCCACAAAGGAC
C002TNE-001	SYN30733	6	159,316,877	C/T	TGACTCAGCTTAAACAATCTTATTCATTAATCTAAAAGGGTAATTAGCTTGCACTCCTGC[C/T]GTTCCAATTTGTAAGTCATTTAGGACTGCTAGCCAGAATGCTTGAGATAGCCCATTGTTC

PZA18337-9		6	159,592,671	C/G	TCTGCATCAGGACGCGCTNTTCAGAAGGCACGAGAGCTTCAGCTTCGGCAGGCCNCCGCAGAGACACGGGCCCAGGCTCCAGCCATGCNTTGCCCTGGAGGAATCCAGCCTCGGCGAAGC[C/G]AGCGCGAGCGGTTTCCAGAGGCAGTTCAGCGACAGGAGCGTGTCGTCCAGGCTCAGCGTCGTCTCCGAGTGCGACACGGTCTCTTCGGCCGGCGACCAGGAGCACAACGAGCTCGTACGG
PZA18337-7		6	159,592,703	T/C	CACCATAGTCTGGGACCCCAGGAACCGATGCCAGCTCTGCATCAGGACGCGCTNTTCAGAAGGCACGAGAGCTTCAGCTTCGGCAGGCCNCCGCAGAGACACGGGCCCAGGCTCCAGCCATGC[T/C]TTGCCCTGGAGGAATCCAGCCTCGGCGAAGCNAGCGCGAGCGGTTTCCAGAGGCAGTTCAGCGACAGGAGCGTGTCGTCCAGGCTCAGCGTCGTCTCCGAGTGCGACACGGTCTCTTCGGCCGGCGACCAGGAGCACAACGAGCTCGTACGGAACTACATTCGCGGGGTGCGCGAGTCGCCTGGCCTGCTCGGGCAGGACGNCGANGGCGACGCTGTGTGTACCAGTACCGCCGGAAACGAGTGCTCGGAC
C001UT6-001	PZE-106104150	6	159,598,074	A/G	GAGGAAGGGCGCGGCGTCTGGAAACCAGCTGTGGGTTTTGTCATGGCGGA[A/G]TGCTTTTGAAATTTGAATTGGGCGGGACTCAACCTAGCGCGTGACTTTGA
PZA13773-6		6	160,392,479	A/G	GAACAGCAAGCCCAACAAAAGAACCCAGACGCAGTGACAAACAGTGNTCCGAGCCAAGGCCAGGAGCCCTTGAACAC[A/G]TTTCCAGATTCAGACGATGGAAACACNAACACCGGTCCGTTGAATGGCCANGCAGCAGCTCATGTGAACATGGAAGCTGCCNTCTCNACGGAGGACGTCATACGGGCTGGCGGGTTCGGN
PZA13773-11		6	160,392,530	C/T	GAACAGCAAGCCCAACAAAAGAACCCAGACGCAGTGACAAACAGTGKTCCGAGCCAAGGCCAGGAGCCCTTGAACACRTTTCCAGATTCAGACGATGGAAACACRAACACCGGTCCGTTGAATGGCCA[CT]GCAGCAGCTCATGTGAACATGGAAGCTGCCWTCTCYACGGAGGACGTCATACGGGCTGGCGGGTTCGGWGCGAAGGAYGACATYGGCAGCCTCCTCCCGACGGCAATTGATTCCACCGATTTTGAGGCCTCGCTGCGGGATKCCCGTGGTTTCGAGGGCGAGAAAGCGGCGCCGTCGCATCCTGGACTAGGGTGGAAGGGGGAGAAAGCTGATGGTGAACCAAACTGGCAGATGTGGCGAGCA
C00128-01		6	160,664,513	G/T	GATCCTCAAGGATCTGAAGAAGGAATTCTGCTGCAATGGTACTGTAGTTCAGGACCCAGAGCTAGGCCAGGTAAGANNCGAGAACAATGCNTTTCAAGCTNNNAAAANTGGTATCTGCNGGTTGGTGNNTATACTGATCTNTTTGTNNCNGCTNNGCAGGTCATTCAGCTCCAAGGTGANCAGCGCAAGAATGTTGCTACTTTCCTAGTTCAGGTATTCAGAATCTTCAGACCTGGCCNAGNTGAATACTGTTTNACCATANNGATANATGTTCNATCTGTTAATACTGATNGTGNNATTATTNCTTGTCTTGGTAGGCTGGGATTGC[G/T]AAGAAAGAGNACATCAAGATTCACGGGTTCTAAGGGACCTGTAAATGCTTGTGNCCTATATTGTGTGCCTCNACATATTGGGGAGCTTGNAGCATCGACANGTTNCNAGTCATTGCTTNACTTATATAAGAACATAAGTAGTATTTNGCTATTGTCAAGTGTGCCTTGCTTGA
PZA9758-28		6	160,665,937	G/T	AAACTCGTGAACGCAAGGACCAATGGACGTAAGGTACAGCTGCACCAAGCGCTAGGACAGATGATATAGYATGGCATGCTCGTTCATAYTMGTCGTCGTCAGAAAGAACACCATGGTCTT[G/T]GCCTGGTGAGGGAGGTATCTGAAGCGGRCACCTTTGCCTGKTGAGGGRGGTATCTGATGCGGCATTGGTCCTCCATCGAGTGTTATAGCTCGCTGCCCTTG

PZA9758-20		6	160,666,066	C/T	CCCGATGCCGCTCTTTCCCCTGATCTCAAGAACGGTGAGGCGTGCCAAGTCTCACCGGGTGTTGGGGAATGGAGTCCGGACCTGCTGAACCCTCATGTGGTCCGNGCTATGAAAGGGCGCGTCGAATGGCCCCAGNNNNNNGGCACCCAAAAGCGCAGC[C/T]TGAAGTCCAAACTCGTGAACGCAAGGACCAATGGACGTAAGGTACAGCTGCACCAAGCGCTAGGACAGATGATATAGNATGGCATGCTCGTTCATANTNGTCGTCGTCAGAAAGAACACCATGGTCTTNGCCTGGTGAGGGAGGTATCTGAAGCGGNCACCTTTGCCTGNTGAGGGNGGTATCTGATGCGGCATTGGTCCTCCATCGAGTGTTATAGCTCGCTGCCCTTG
PZA8127-62		6	161,706,411	T/C	TAGGTCGTTGATATAAAKGATTGTATGGATATTATGACAACAAYATATATAGGCAGGGCCAGCACACCAATRAAGAATTTAGAATTCATCACTCCCGTGGACGAAATTAGCGAAAATMTGTACCAGTGTTCATAGATCTTAGCTGCATAGTTTTAAGTCTCAATAGTATGGCACAGTGCTTTATATATCGACCATATGCTGCGTTGCTGACATTTTTGTTCCTTTCCTCTGCAT[TC]TGCAGCGTCATAGGCACTACGACTACCATGTAAGTTACGAAGTAKCTTCATTTCTTTTCTTGGAACGCCCTAAGCTACACCAGAAAAGCTACCTGACGTTATAAATTGTTTGTTTCGTGTTCAGGACGAGCTATAGATCMTGTATTCT
C00373-01		6	161,968,526	C/T	GCCCCGCTCGTGGCACTGTTCCAACAAGAGGAACAGCAGCGACCGAGCCATGGACGACTC[C/T]GCGAACTACAAGAACTCGAACCATGACTACTGGCAGGTACCCAAGCACGAGGACAGCTAC
PZA597-18		6	161,968,528	T/C	GTAAGCGGTATTTATACACNGGTGCTGATTTGGANCACAGCGAGGGCGAACATGACTTCGTTGTGGAGCGCTCGCCTCGTCTGCAATCNCCTATCTCGAAGGAGTCTTCCTTCCACCAAACCCCACCTCATCTCAGGGCGTCAAGCAGAGACGCGCACGTTTTCCCACACGCGATATCCCAGTGGCAGGCGCTGCCCCGCTCGTGGCACTGTTCCAACAAGAGGAACAGCAGCGACCGAGCCATGGACGACTC[T/C]GCGAACTACAAGAACTCGAACCATGACTACTGGCAGGTACCCAAGCACGAGGACAGCTACAGCATATCGAAATGCGTCCAGATCCTGAACGGCATGGCGGAGCTGTCG
PZA597-12		6	161,968,745	C/T	GCAGAAGGCATAATGGCTGACGACAGGTAAGCGGTATTTATACACTGGTGCTGATTTGGA[C/T]CACAGCGAGGGCGAACATGACTTCGTTGTGGAGCGCTCGCCTCGTCTGCAATCACCTATC
PZA8065-28		6	162,390,684	C/G	TCATTTGCTTAAGCTACCATAACTTATGAGTGCATTTGTTCTTTGCCTTCTTATCCTTCACATTTGAGCTTTCCTGTTCAAACTTGCCTAGGCAATTAGGCATAAGGYTACTGGGTATCTCTGACCTGATGTTGCTTTCCMCTGCAGATCGGTTTCTGGAAGAAGTTCAACCTCATATTCCTCTTCTTCCTGCTCCGGAAGCTGATACTACCYTTCTACTCCTTCACCCTCTTCTGCGT[C/G]ATCCTCCCCATGACGATGTTCGTCCCCGAAGCCGAGCTCCCCGCGTGGGTGGTGTGCTACATCCCGGCGACGATGTCCATCCTCAACATCCTCCCGTCCCCGAAATCSTTCCCGTTCATCGTCCCGTACCTGCTGTTCGAGAACACCATGTCGGT
PZA9392-10		6	162,628,110	G/A	AGCAGATAAGCCCACAGGTTTCAGGCGGCAATTTCGAAGTTGGACAAGTGACCTACAGGTTCAACTTCATGAAATCAAGAGGGCCGAGACGGATGCAGTGCAATATCCAGTGCCCTGTAGGCCAGGGCAGCGCTTCGGATCCRYCCAAGGAGAAAACACCCTCGCCGAGTTCCTTGGACCT[GA]AAGAACAAGGCTCCAAGATGGCACGACCATCTTCAGTGTTGGTGCTTAAATTTCCACGGYCGGGTGACCGTCGCCTCCGTGAAGAACTTCCAGCTCGTCGCCACAGCTGGCTCCGGYGGTCCATGGGGCGTCGGAGACGAGGAGACGGTGATCCTGCAGTTCGGGAAGATCGAGGACGATGCGTTTACG

PZA9392-11		6	162,628,170	C/T	AGCAGATAAGCCCACAGGTTTCAGGCGGCAATTTCGAAGTTGGACAAGTGACCTACAGGTTCAACTTCATGAAATCAAGAGGGCCGAGACGGATGCAGTGCAATATCCAGTGCCCTGTAGGCCAGGGCAGCGCTTCGGATCCRYCCAAGGAGAAAACACCCTCGCCGAGTTCCTTGGACCTRAAGAACAAGGCTCCAAGATGGCACGACCATCTTCAGTGTTGGTGCTTAAATTTCCACGG[CT]CGGGTGACCGTCGCCTCCGTGAAGAACTTCCAGCTCGTCGCCACAGCTGGCTCCGGYGGTCCATGGGGCGTCGGAGACGAGGAGACGGTGATCCTGCAGTTCGGGAAGATCGAGGACGATGCGTTTACG
PZA3846-10		6	162,815,074	C/T	TATWACTAACATANNNCTTGGTTCATTCACTTTATTTATTTGCTTTCAGTGGGTTTTGCATGACTGGGGCGATGCTGATTGYATCAAGATACTYAAGAACTGTAAGAAAGCTATACCTTC[C/T]AGGGATGCAGGAGGAAAGGTGATAATCGTTGATATGGTTGTTGGAGGCCAGTYGTCAAACRWTAAGCACAAAGAGACTCAGGTCTTGTTTGATCTCTTCATCATGACGRTCAATGGCGCC
C002PNK-001	SYN26189	6	163,793,713	C/T	GTCGATTATGTGCAATTTCATCATCAAGCCGATTTATCATACAAACCTTAGCCTTCTTAA[C/T]GTTCTCCAATGTCCCAATTTCGCAATCAAATCCGTCTTCCTCATCATCCAGTCTATCGTC
C04880-1		6	164,018,180	G/C	GTAAATTTACTATTGATGGAGATGGAACTCTAACCTACAGGAGGCTGGAGGCTAGAAAGCAATAGTTGGTGGGGGCATTTCAGTCATGCTGCCTAACTAKWACTCTAATCTGCTCAAACAGCGTGGAACCGGGGTGGGGGGGGGGGAGCTTTTGGATTAGAGCTTATTTTATTTATTGAACTGGGGTMATAGTCTAAAGG[G/C]CCAGGCTAGGTTGTTTGGTTACTTTTTTCGAYGCGGGRAAACTGAAAAGTATGTTCGTGATCAGTTTATTGAAAAATGACATTTTTTTGTTTMSWGWTCAYTATGCTGAGTGCTTCTGCTAATAAATTRTCGAGGCTTAAGGAGCAAGCTGAGGAGTATGCAGCCTTGATCATKSAGGAACTTGATCCTGAAGGGCTTGG
C001W0P-001		6	164,186,247	C/T	TCAGCAAGTGACTTGTGTCGTACCCGTGTAGCGCGGACGGCGCTCCAAAC[C/T]GGGTCAAGTTTTAAGAACTCTGATGCACAAAACACCACTCAGTCATATGA
C05007-1		6	164,392,836	T/C	CAGAnGATGnCAATTTTTGGAGATCTACTTGAAGAGCTGTnGGAAGTTTTTTTnCCCACCAAAACCTCTATTCCTATCATAGGAAGGATACAGAGAGGTTCTTGGAGTTGCTCTAATGATTCTATCACCACCAGTTTGTTAATGCAATCCACTCCTGAGTGCGGAATATTAACTCTAGCACTTGTGCTACACTTCATTCA[T/C]CAACAnAnGGGTTTTGCTTCnCCnGAGATGAACATGGGCTGTTTTATGCACTGGATCTTGGAGGGACCAACTTCCGTGTGCTGCGAGTCCAACTAGGAGGAAGGGAGAAACGTGTTGTCAAACAACAGTACGAGGAGGTTTCCATTCCACCGCATTTGATGGTnGGGACTTCCATTGTGAGTACTCGCTGTTCTATTTTT
C04980-1		6	164,782,727	A/G	TCTCGGTCYGGTAGCTTTTCTCTACTATATTAAAGCAACAGTWTTGACGGTCATCCTGTGTCATGTTTTTACAAAGAACCCCTTGCATTTCATAGAAATCTACCCACCGTCTCTCCTCCCGTAACAACTCCCATCTCCGTCRCATCTGAGTGCCCGACTTCCTCGTATGGATAGGYGCCTCCACCTCGCATCCTCCTACC[A/G]TCCCTTCTCTACTTCTACTTTCCYGCTTGATCCAATCTTCATAAATCACGCCGATCGGAACCACCCAGAGAAAGTGGAGCTGAAAAACAATTTTCACCCTCCAACCTTCGTCCTYGTGCAGAGTGTGGGCGACGGTGGACAAGGTCCACATGATGGTGGGAGCAGGCCGCGACCGACGAGGCCGCAAGGGAGGAAGATGA

C04870-1		6	165,173,096	G/C	TTACCTAGTAGCCTAACCTTTCGATTTCCTAGTTTCCGTGTGCACGTAGC<br>GGAGAGCTGGAAGAAGATGGCGTGTCAACATGTGCATnCnTATGCAGTGT<br>AGGACTGGCCACTTGACTACTTGAGGGAGGAGCACACTTGACTTGCCCCC<br>ACCGGGCCCGCCCCCACAGnCCnCnnCCACAGCAGCACACGAGCTGCTTA<br>[G/C]GGGGAATGGCGTAACTTGATCnCnTGCATGTCCGAACGGTTTCCA<br>GAGATTCAGGACTGCnAAGTTTnAAATGGCTAATGGTCTGGGGCTCTGGG<br>CGCCGAGTATACATGTGTCCATCTACTACATTTTCTGGGATCGTAGGACA<br>GnGnACTGCATCTGCATGGAAATGGAGGACCAGCACCAAAnCCCCTGTTG<br>GACCG
PZA1350-20		6	165,640,040	T/G	GGNGCAGATGATAGTTCAGCGAGTCAAAATTACTTTTTTCAGGACAGTAA<br>CCGTGGATTATATATAAATAAAAANGNTAAATTTGGTCAGTAAAGGGCCA<br>AATTCAATTGATTGGAATAAAAGCGAAATGGGACAGTCAGNCAGAGACAG<br>TCAGGCGTGCACATGGAAGGATAAGGTGAGTTGTCCATCCATCNNNNNNC<br>TGGGGCCNGATCGTTCGAGATCCGCGCGCAAGCGCAACCCAATCACCCGC<br>CCCCACGTGGGCCACGTCGATGCCGATGGCGTCCC[T/G]CGCAGGGCCC<br>CACACCGCAGNTGCCGTGGAAAGTTAAAAGCGCCCGCGGGGATACACGAC<br>GACAACACGAGGAGAGACTGCGGCAGCGGAGTGCAGAGAGC
C00283-01		6	165,730,692	A/G	CAACTCATAACACTGGGATGGATCCATCACGGTAGAGCTGACSATGATAT<br>GTCCAAARGCTTCTGCTGCTAGTTGTTGGTCGTCCGCAAGAGACTTGACC<br>C-<br>ACAGGTTTTTTTCGTTGGGCCTCAAGAACTGTAGGTGCATTTTCCATGGA<br>GGGTTATGATGCTTAACCCCACTCTGGTTTCTG[A/G]TGGCTAAAACAT<br>TCTCTCCAGTCGGCTTCTACCATGAGCCCTGGCACTTAGTATATYCCTAA<br>TGTTGTATGATRGTTGAACTTGTAAA
PZA2108-61		6	165,730,833	C/G	CNNGNNNCCCCNNCNNNNNNNNNNNNNNNNNNNNNNNNNGNNTGNNNCNNC<br>ATCATTNGTGTNACGAGGGTGATGAATCCAATGANNNTGNTGCACAANCT<br>GCTGCTGATTATCTNATGAGGCTCGCACTGAAGAAGGGTACCGAGGACAA<br>TATCACTGTCATTGTNGNTTGACTTGAAACCTCGAAAGAAGNCCAAGAGC<br>AACTCANNACACTGGGATGGATCCATCACGGTAGAGCTGAC[C/G]ATGA<br>TATGTCCAAAGGCTTCTGCTGCTAGTTGTTGGTCGTCCGCAAGAGACTTG<br>ACCCNACAGGTTTTTTTCGTTGGGCCTCAAGAACTGTAGGTGCATTTTCC<br>ATGGAGGGTTATGATGCTTAACCCCACTCTGGTTTCTGNTGGNTAAAACA<br>TTCTCTCCAGTCGGCTTCTACCATGANCCCTGGCANTTNGTANATNCNNA<br>ANGTTGTANGATNNTNNAANTNNNNANNNNNGTTNNNNNNNCCNAANNNN<br>NNNTNNANANTNNTNNNANANANNNNNNNAA
PZA14734-27		6	166,021,497	A/G	ACTACATGAGCTTGACCAAGTCTGCNAAGGCGAGGCTGANTGGNTGCAGC<br>GGCNGCAGNCATCACAGGTCGTTCCAGCGACCACGGTCCGGGGACATGTC<br>GAGGGTGACACTGTCTTCGATCGACACCCAGAGCAACGCGGGCTCGGAGA<br>TTTC[A/G]GTCACCTCGAAGAGACTGAACAGCATGTCCCTGAACCTGAA<br>NGGCCGGAGCTTGGACAAGGAGAACGAGGAGGATTGATCCANNNNCCAAC<br>GGACAAAGCAGCTGTCGTAGGTCTGGTGNNAGTACTANCACNCGTTCAAA<br>GCAGCATCTCTGTATTTACGGAATTTACGGAGGAAGACGNNGTTTATCTC<br>TTTCATAAACTCCACACATGTNNNNNNNGANNNNNGAGA

C04582-1		6	166,294,962	C/T	GGCACTCTGNCAGTCTGCCACTGACTGCGGCAGACACGGCCACCCGCGCTCCGTTCTCTTGTACGCACACACGCACACGCATCCAGAGAATTCCTGTCCTGATGATGAGCTTATTACAGCCCNNGGGCGAGNAATGATGGCACGTTGCTTTCCAACATGGGGGGACGACGACGAGGACGATCGGCTCCATCNATCCATGG[C/T]TCGCCCAATAGATACGCCAGGCCCAGGCCGNCCNNAAGCTTCTGCGTCTCGCTCGCCCCCNCTNNTTTGACCAAGGGGTGAAGTGTCGGCAGCTTTCTGCTANNTATCTCCTGACCTCCTCTATGGCTTTGCTAGCTCTATACAGGAGCAAGAGCACGATCGGAACACGGCACGCTAGACACTCGAGCCCGANNGGCCCG
C04608-1		6	166,301,439	A/G	TTACGAGTGACTAAACACTGCTTATGGAAACTGTGGAAAGGCAGCCATGAATGCGGCCGTGGACTCGCGGGCCGGGCTASGTMTAATTAATCGCGACCACGCCGTGTGGCTGTGGTCGACCGCGCGCGTCGTCACGTTGCCTTTTCCTCTGCGCGCTGGTCCACTGTGCAAACGGGGCSGGAGCCGGCSMCGGCCAGCCA[A/G]AGCAGCCAGGCCCGATCGGATCGGATCGGCTACTAATCACTCTTGCGTTGGGCTAGCTAGCTATAGCTGGCTAATCACRTCGCTAATCCCGATCGGCCTCACCAATCACGTCCCAGTWAAWTGGCCATGGCCTGATGGCCTGCCTCTGCCTGCCCTGCCGACCTCCGCGTCCCATCCTGTCCGCACGTCACCCCTGAACA
C04578-1		6	166,309,977	T/C	GCTTCGAGTTCAAAGGAGCAGAGCAGTTACTCCGTTCATAAAAAAAAACGAGACTCGTGANNCCATCGCTGTCAGAGGTTTATACAGATGACTCGCNGTTACTNCTGTGGTGAATTTAGCTTTTNAACNAGAAGGGATCGAGATGTAGGCTTGTCAATTGTCATGATGAAGCAANGACTGAGAGGGAGAGGTTGGTCGGT[T/C]GGAGATCTGGATCCNCACCACACCATATAGGCGTAGACGAAGGAAGAAAGAGGTTAAACGCCCCAAACAAACAGCACGGGCGAGCCGGCGACACGAGGAAACGACTGGAAATGCACGTACGGTCACGGACACCCCGTTCACCACCGCAGCAGCCAGCAGGGGAGGGGCTGAGGGGCCACCAGTCCTGCCACACAGAAGGC
PZA3153-63		6	166,318,078	T/G	TATATAAATAAATGTGTGTGCTATCACGTTCCCACTCTCTGAATGTCTGATCATTAACTTGTTGACCTTGTTCTTAAGGTAAAGCTACTCACTCACTCCCATCTAGTACTGCAATTGATTCTTGTCCCCCCGTGTATGCCTTCTGCAGAAAAAGAAGTTCTGCTTCTAAGATCTGACCATGGAGCAGCTGGTCATGTCCGGAGCCGTTCGTGTATATTGTTGGATCGCGGCTAGTACGGTGAATGATGTAATGTGTCCATCTGTACAAGTGTGTAAC[TG]GAACGGTGTAAGTATAGTAGGAAAACAATGTGTTAGCTGTGCGGTAGCACTTGTGTGCTAGCCTAAATTGTAATCTTGCAAG
C04564-1		6	166,661,962	G/A	GAGTAAATATGACCTCAAGCACCATATACAGAAACAATTACCCYTCACAGATAGTACAAACTATAAGAYGGGTAATGKTYGCTTTACTCAATCAATTGCATTAACTACCCTTTTCTCAGCTCAAAWGTCAATATGTCATCCAAAAGCTATTTGAAACATTCACTCAATTAAGATCCACAAASTACAAGCACAACTATCTT[G/A]AGCAATTATCGTSAATTCCATCTTACGTGAATAATTGWYGTYGAKATTTGGACTTCAGCAGTTGGGAGAGTAAAAGGCAATCAGTCGCKSAYGATCTCACTTCCATCTGAAYTTTCAGAGGCATCAGTAACAGCACCTAGCTCTTCAGCCTCTTCTCTGTCGGTTTCCATAGCATCAACTTCRGACTCGTCATCATCAGT

C04561-1		6	166,805,322	A/C	TGGGGCGCGGCCGGCCAACGCCGTCCTTKATSGCGTCGGTGAGGACGCCG GTGATGAGCACGGAGAAGAGAAGGCCTRSTCACGCATGAACCGAACCCAC AAGTGTCAGAAGAGCCTGAAGAACAGCTGGAACTAACAAACAGGAAGCWK GCCTAGAATSGCGTGGTGCATGTCGTACACGTTCCTCCKCTTCAGGTATA [A/C]CCCGACGAASACGACGATCGGGCCGAWHACSGCGTACACCTGCGC GATTCAATTCMAMAGCAGCCATCTTTCAGTAACCCACCGCCATCGATTTA TCTCTTCTGWCGTCGCAGGGACTGGTCTGAAGGCTGTGTGCGTGCGCGTG TATGCGTGCATGTACCGGCACGGCCCAGACGGGYACGGTGTTGCTCTTCA GCGGG
C04595-1		6	166,805,332	C/G	CCGGCCAACGCCGTCCTTGATGGCGTCGGTGAGGACGCCGGTGATGAGCA CGGAGAAGAGAAGGCCTGCTCACGCATGAACCGAACCCACAAGTGTCAGA AGAGCCTGAAGAACAGCTGGAACTAACAAACAGGAAGCTTGCCTAGAATG GCGTGGTGCATGTCGTACACGTTCCTCCKCTTCAGGTATAMCCCGACGAA [C/G]ACGACGATCGGGCCGAWHACSGCGTACACCTGCGCGATTCAATTC MAAAGCAGCCATCTTTCAGTAACCCACCGCCATCGATTTATCTCTTCTGT CGTCGCAGGGACTGGTCTGAAGGCTGTGTGCGTGCGCGTGTATGCGTGCA TGTACCGGCACGGCCCAGACGGGCACGGTGTTGCTCTTCAGCGGGTACCG GAGGT
PZA16537-57		6	166,895,486	C/G	TATTTTGGCATGCTTGATGGTTTGCTGGCTGCAGAACAGCTTCCTGAGGA ATACCAGGACCGGTGCCAGGTAAACCATTATCGCAGTCCCGGTTTCATTA TTTCATTCATATAGCTCACATTACGTTTATCATCGTTGCGTGGCTGTTTC AGGATATACTGTGTAACGACTGCGAGAGAAAAGGCAGGTCACGGTTTCAT TGGCT[CG]TACCACAAGTGTGGTTTCTGTGGTTCTTACAACACCAGAGT TATCAAGACTGATACGGCAGAGTGTTCTCGCCCTCTATAATTTCTTTTAT GCAATCCATTTGTATATATATATCGACGGGGTAGAAACCTTGTGACCGTA GGTGTGATAGTTTTTTTATAGAGTGAACTGGGGGAGGAACCCCACCTGGA TGTATTATGAGGAAAAAGTGACTGTGCACATGAGGAGTCTGAGAATTACT CGCTCCCGTCCCAAACTAACTGTATTTCTACGATTTGAAATATATTCGCT CATATATTTCCTGTGTTGCCCTTCAGCTGTTTCCTGGGAACATCTCGGTT TGTATCAGCGATACAGTTGGAGACCTTCTGAACACTGTCCCCAACTCAAG CCGACTCAGTTGAGCTGAAGAGCAAATAAATACTCAGGTTGCGATTGACA TGGTACATTCTCGTCTGATCTTTTACTTGGAATGCTCTTCTTACAAGTAT GAAGTATCCTCTTCAGTCCTCTGACCACAAGTAGAATCTTCCAGAGACCC AGAGATCCTAGATTGCGAGCACCTAATATTTTTTTTTGGTATGCCAACGT GTCACATAT
PZA2632-23		6	167,235,258	T/C	GTTGTTTGCYGCACTTAATTTTTTTATATGGTAAYTTTAGTAAGTTATGG ACAACTAAMTGAGTAACTGCGATMTYCAGGAATTGGACAGACAAGTCCCT CTGATGCCTAGAACTGGGACGAAATGGATGATAAGGTCTGTTATCTGGCA GCCATTAGTTTTCGTTGGC[TC]CCTTCTAGAAATTGTCTAGCAACTCTT ATTTTGCTACATAATGATGATTGTAGGTGGATAGAGCCAATGCAGATTTG AAGAATACCAACGTGAGACTGAAGGAGACTGTTCTTCAAGTAAGAACTTA GCACTTCCGTTCTACACTKGCTAACTAATGCTTGAGTAGTTTGGTGACCT CGTACCATTTGTTTTTC
PZA7922-8		6	167,241,015	A/C	AACAAYGATTATGAYGATGAACAATTGAGAGAAGAGCCTCCAGCAAAGGT GCTAATTGAC[A/C]GGGTGCAAAGAAATATCTTGGCCGACAAGCCCCGA GTCACCAAGTTCCCTATCAACTTCC

PZA10105-6		6	167,632,994	G/A	TCTCAGTTAGCACTTTTAACAAGTTATGTTCATTCATGGATCAAACATTGGACCAAATTGTTGGCATGAGACCTTTGATTTGTAGTAATAGAGCAAAGTGTATATTGCATGGTCCCTTCCTCATTATTATTTAATTAATTGGCATTTGTCTACACTATCAATGTAGTTGCTGAATCTGTATTAACATGCCTCACTGGCAGGTACATGCAATTCTGAAGGACCTCGTCCGCAAGGCTATCAATGAAACACATGTAAACTTTCCTCTTCTCTACTATTATATTGTACNNNNNNNNNNNAGGAGCTGAAGCAGTTTCCCACCCTTCGTGTGGAAGTAGGCAATGCAGCTTTTGAGTCATTGGATAGAATGAGGGATGAAAGCAAGAAAAATACATTAAAGCTAGTTGACATGGAATGCAGCTACTTGACAGTAGATTTCTTCAGGAAGCTTCCCCAGGATGTTGAGAGGGGTGGAAATCCAAGCCACTCTATTTTTGATAGATATAATGACTCTTATCTTAGACGAATTGGTAAGGCCTATATGATCTCTGCATTTTTGCTTGATAGTTGATTATTTACTGCTTAACATCTCACTGTACTAAAC[GA]ATCAACAGGACAAACTGTTCTGTCATATGTTAATATGGTGTGTTCGACCTTGAGGAACTCCATCCCAAAATCTATTGTTTATTGCCAAGTGCGAGAGGCCAAGCGCTCGCTGCTCGATCACTTCTTCACTGAACTGGGAGCAAGAGAGGTGAGATTTGTCATCATTTGAAAGGAATGCTTGGCCATATTTTTTATTACCATAGCTTTCATAATTTCATTTAGTAGTGTTCACCTTGTTTATTTGTTCATTCAACATATTGTAGCTTCCTTTTGGTCGTTACTTGCAACTCGTGTGCATAGTTGTGTTGTTAAGGCGTCCCTAAGGCGTCGCTTAGGCACTCCTAAGTGCTAAGGCATGCGAAAACCTTAAGGCGTCCTGGTCGCCTTATCTCGGAGCCTAAGGCGTCCCCTTTGGACGCCTTGGAGTGATGTAGGGTTGGATCCTGACCTGAGCACGTGATTTGAAACCGTGTCCCTGGAGAGTGCCATCAGCAGCGGCGGCGGTGGCCCCTCCCCTCCGCGACCGCGAGCTCCAGCTGCGATGGCGAGGTCCCCTGACCCCCTCACCTCTGCGGCAGCTGGGAGAGAGGGAAAAGCGGC
PZA6564-19		6	No BLAST Hit	T/G	ATTCAGTTGTTGCATGAATTTTGCATGTACAAGTGAACAACATGCGTACAATTGCTCAAATCTTTTCTGTCGAGGAGCAGATCATATTGCCCAAAGTTACTCGGGTATAGRCAATTCTTCTAGATATAAGTTTAACCAAGAGTGTAAAACTGAGCATCCT[T/G]AGTTTGGAGTATGAATCGGACWAYAGGTNNDNNNNNNNNDTCTGTATCCTCGACGACACCCACATGCATNGCTCAGCTCAAANTGGCCACACATATATAATAANCCGATCAGCCACAGGCTAATTAGAGGCAATAGCGAGCCTAATCCTAACGAGCTCTGTTCATCAGCCATCACATTATTCGTCTCYAAGCAATGGCCTCTCAAAGA

Table S4. Markers used to fine map the C6QTL in populations generated from crossing MLN-susceptible elite maize lines with KS23-5 and KS23-6.

Marker	Public Name	B73 V4 Chr	B73 V4 Physical Position	SNP	KS23-6 Call	Sequence
124	SYN10489	6	158,532,450	A/G	G	GAACAAATTTTATTTTCTTCAGCCGGAGATGTTGATGTATATGATCTCCAAGCCTTATGC[A/G]ATAAGGTGACCTTTCATCAATCCCGATATTTCTCAGTATTATGTAGTCAATGAACTTAAT
134	PZE-106103556	6	159,231,857	A/G	A	CCCTTGATCGGGGGCCACCTTAACAATAGTTGCAGCCTCCGGTCACACAA[A/G]CGTCATATAAAAGGGATTAAGAGGCCGACGATTTAGCTAACTGTCGGGGA
139	SYN30728	6	159,374,975	A/G	G	GAACGAGGCCACACGGATCCACTTCGTAGCCGACCATTTCTCGCCTTTTATGACCGGGCA[A/G]CCACCATGGAGGCTCACCGAGTCGGTGGTGCCGTCTGGGTTAAGGTTGAAGAAAAGGAGG
147	SYN23866	6	159,650,213	A/C	A	CCGGTATCCCGACCTCCGTACGTGTCTCAGATACGCGCCGCGCATGCCTCCCCCCATTGC[A/C]GCGCCTGCTTTGTCGCTGACTGAAGAGTGTCCGTGTCCATATTTCAGGCGTTTCTGTGGT
150	SYN23859	6	159,734,571	C/T	T	TCCTGCTGCTGCACAATACAAAACCTCGGTGGCATATAGAACTAAGAGTACAAGAACGTA[A/G]ATACGAGGAAATGCTCTCTGCATGCTTGCTTGAAGCGGACCCCCTATATCCGCTTCAGAA
155		6	159,894,747	A/G	G	GAAGGTACCGACGGCGCGCAGCCTGGGCAGCTCGACGAAGGCCCAGGGCACGAGGAGGAA[A/G]CAGAAGCAGCACGGCGCGACATAGTAGAGCGAGGTGATAGGGTTGAGCGAGATGCCCTTG
233	SYNGENTA17033	6	160,129,054	A/G	A	TTTNNNNNNGCTTGTAACTGTATATAGNNNNTTGTTGAAACTTCGATTTATGCTTTCGAA[A/G]TGGCTGTACAACAATGGCACTCTGCAGAAGTTTATTTTAAGCTTTGTGTTCTCATTGCTT
234	PZE-106105727	6	160,261,241	C/T	C	GCAGATCCAGTCAGCAGCATTTCCAATGGAGGGTAAACTATCTTATAGAG[T/C]CCGGTGGCAATCTTCACGCCAATATGGTGATATCAAAAGGAAAAACAAGG
236	PZE-106105803	6	160,351,140	A/C	C	AGTATGTATCAAGAGGTGGTGGCAGTTACTGGTGATGGAACCAATGATGC[A/C]CCAGCATTGAGTGAGTCAGACATAGGTCTGGCCATGGGAATTGCCGGCAC
162		6	160,352,808	A/G	A	AACCATCCATAAATGCACATCAGTAATGGGGTGTAGGTTCTAGCGAGAAT[A/G]CATGAAACATTGCGCCTGTTCTAATGACGTAGCTGCGTTTAATTTTCTCA

163		6	160,396,421	A/G	G	CAGAGAGGGAGCGATTCGGGAGGGCGGGAAGAAGGCTTTGTGGACGATAAAGTGGGGAGG[A/G]GATTTGCTTATCGCTGGCGAGAGGTCAGGACGACGGTTCCGGTGGGTGTGTGGGGGAGGA
165	SYN28693	6	160,517,876	A/C	A	ACGAGACTGTCTATAGGCACTTGCCTGATCTTTAAGTTTGCTGGAACACTTTGTGAGCCT[A/C]TCTAGTTCAGCTTGCTCACTAGATATGATGCCATCCAGCTCTTGCATTCTGCGAAGCTCG
238		6	160,529,079	A/G	G	GCTTTGGCAGAACTTGCTACGCCATGCTTTGCATTGCAGCACTGCAGCCC[A/G]ACCTGACCATCAAGCATTTCTTCGGCTAATTAGACTTTCTAACTTGATTT
166	PZE-106106224	6	160,539,241	A/G	A	CGTTTCATTTAGCTTGCTTGCGATCTATGCAACGTCGCCTTTATTGGTCG[A/G]TTAAAGATGTACTTACCAAACGGAGTCGACGAGTCGTCTCATCCCGCGTA
239		6	160,544,296	A/G	G	TGGTGGCTCCAACGATATCAACTAGGTTTATGCAGAGTGACATGTCTTTC[A/G]GTCTTGCTGTTGTGCTCCGTGACCGTGACTGAAGCACGCGAGCTCAATGG
240	SYN28691	6	160,545,528	A/G	A	GCAGTTCCCAAGCACCCAGAAAGTGCAACAGATGGTCCCGCAGTGGCTGCAACGGAACTA[A/G]TCGCTGGATCGACTGGGCTTGGGGTGGCACTGTAATCTCATCTTCTTCATCAGTTGGATC
167		6	160,559,144	A/G	G	GAGAAGAGACCAATGATGCAATCTCGTGATGTTGATAAATATTTGAACAAAGCTGACAAC[A/G]TTAACCATGTGCTCATGAACTCTGGCGACATTTCTAAAATTGGTGGTCNCTGCCTGCATG
353		6	160,580,380	T/A	A	TTTTGTACTGGGGATTTCTGGGATCAAACGTYTCATACARAWTTCTGGGA[T/A]CAAACATTTTCCTTGTATGAATTCTAGTGGACGCACTCTATATTTTGCTG
443		6	160,582,950	G/A	A	CAGTGATGCAACAACGAGGGGACCCTTTGCTTATAGTAGGATCTCCATTTG[G/A]CCTCATGTCACCCTTCCATTTCTTCAACAGGTAATGTTTATTCTACTGCTT
371		6	160,584,510	A/G	G	ATGCATTGGTTTATTAGCTATATATAGTTCGGAATCGGGATGTAACTGAT[A/G]CACTTCCAGCAATAATCAATTTGAGTTCACTTAACTTCCTTTGCAAAGAG
309		6	160,585,254	A/C	A	GCCGYGTGCCCGTGGGCCGAGCAGATATGGTCGAGAARTRGCGGCGGCTC[A/C]GCTAAAGCGCCCCGCGGCAGGAGCAGAGCCGACGCCGAGAGCGTGGTCGA
441		6	160,585,382	C/T	C	GAAGATGCGCCGCCACGCCTTTCATTTTCACCAGTWAGCCCTCACCCCTC[C/T]CAATTTTGAGATGTTTTACTCGATATAAAATCGAGCTTTCCTAAAGCCCCG

379		6	160,585,536	G/A	G	ACGGCGGTTAGCRAGATAATACGGGTGASCTGCCGATG<br>GTRCRAAGTCRC[G/A]RACTAACCGGACCGACGATTC<br>GAACCATGGCGCAGAAGTGGCGCGCTGCA
382		6	160,591,137	A/G	G	AACGGCTTTTCCTCCATTCCACCGTCCACCGCCAAGCG<br>CAGACGCGAGGC[A/G]GTGTCTTCCAGGAAGAAGCCC<br>AAGCCAACACCCAACCAGGCCAAACCGGC
470		6	160,591,580	A/C	A	GCCAGGATCCGCGAACGCAACCGCCTCTTCCCTGTCCT<br>CTATCGGTGAGC[A/C]CACTGCTACCTCGCGCCTCCT<br>TCCAATGTGTCGTCCTCCACACCGAGCTC
472		6	160,592,435	G/T	T	TTGCTAAATTTTGAATATTAATGAGAAGTAGCGTTAAT<br>AAGTACATGCAC[G/T]TAGGGAAACTGGACATATATT<br>GCTATCAGTTATTGTATTTTTCTTCCTTT
473		6	160,592,825	T/C	T	GGCGTTCGACCCTAGCGGCTAAACTGGCTGCACCGTGT<br>GCGGCTAGCGGG[T/C]AGCGGGAGCCACCAGCCGCTG<br>CTCCACCAAGGCTCATTGTGCGTATTGTG
477		6	160,598,253	C/T	C	CCGATCATGACGCTTCACCACCTCGACGTAGTGAAGCC<br>GCTGTTCCCGGA[C/T]GCGAGGTCGCGCCCCTCGGCG<br>GTGCGGCGGCTGTTCGACGGGCCGGTGAA
461		6	160,601,357	A/G	A	TGGGAGACCATGCTGCGGGACTCTCTGCGACGAAGCCA<br>GAAAAACACGGACG[A/G]CATGGTCACCATCCTCGGC<br>TCCTTCGACAACTGCCTCTCCGCGTTCGAGGCCGCAAT<br>GCGCCCCACCCAGGTTCGCCCGCCCCGTATCA
422		6	160,633,079	A/T	T	AACAATGCTGGAAAAGTTCGGATTGTGGCAACAACTTT<br>ACGAATTAAAGG[A/T]AAACGAATAGGTTGTGCGTTT<br>TGTGCAGGRGCCGACGGTGTGACAGCAGC
459		6	160,634,877	A/G	A	GTGGTCGAGGTTGTGGCCCGTGGTAAATCGGACCTTGG<br>TCCAAATATTTCTTTCTAAC[A/G]GTTGCGCATTTTG<br>CCTGACTAGTAACGAACATCTCTTATGTCACGTCGGTT<br>TC
341		6	160,663,779	T/C	T	GACTGATAATATGATTTTGGCAGCTTCTGCATAAGAAC<br>AACAAATCAAAAG[T/C]KTGATCAGCTCGGTGCCAAC<br>AAAACCTCAACAACCAAGTTTCATGTCTGA
247		6	160,664,367	A/G	A	TNGGCCAGGTCTGAAGATTCTGAATACCTGAACTAGGA<br>AAGTAGCAACATTCTTGCGCTG[A/G]TCACCTTGGAG<br>CTGAATGACCTGCNNAGCRGNNACAAAYAGATCAGTAT<br>AYYCACCAACC
343		6	160,664,565	A/C	A	AAGAAAGAGAACATCAAGATTCACGGGTTCTAAGGGAC<br>CTGTAAATGCTTGTG[A/C]CCTATATTGTGTGCCTCM<br>ACATATTGGGGAGCTTGRAGCATCGACA
169		6	160,664,891	A/C	C	TATTGCCTCCTTGTATGTTGTGATCTAGCAGTGATTCA<br>TGCTGGGTCTTG[A/C]GGCGGCGTCTGGTTAGTCCGA<br>TGCCGTTTCAGATTCAGACCGGCTCGCGA

243	SYN24071	6	160,666,444	A/C	A	GAGAAGTGCAGATCAAGTAGCGGCATCACCGGTATCTGTTCGACAGCTGGAGAGAGGCAC[A/C]CGAAGAAGGCGTCGTCTTTTGCCCGACTGTGGAGATCATCAAACGGCGACGGCGGGAGGA
245		6	160,667,185	A/G	A	TGTTAAAGGGGATCTGAGCCGAGAGAGGAAGAATCGACAAAGGGCCGAAGCCATGAACTC[A/G]GAGTTGATGGATGAGTTCTCTGAGCTGAAGTCACTGGCAAAACGATATCTTCAAGACTAT
428		6	160,678,238	T/G	T	CGCCCCCATGGCCACCAGCCTTTCTCCRGMGGCGGCATGGCCTCCTCGTA[T/G]CCCCTGACCATGCTCCGGATGGCGGCGCAGCGGCAYTCGCGGCTCACGTC
170	PZA00223.4	6	160,736,377	A/G	A	GGCTACGTCGAGAAGGTGGYCAAGGCGTCGTCGCCCGACGAGTACTACGCGGACTTCGCC[A/G]CGGCGATGGTCAAGATGGGCCGCACCGACGTGCTTGTCGGCGATCATGGGGAGATCAGGC
248		6	160,736,474	A/C	A	CAGCACGCACAGTACANGTTGACAGAAACCCGTACAGATTATCTTCGCATNTTCAACCCA[A/C]TCCTGAACCTAGTCAACAAAAATGCCACACGTTGGCCTGATCTCCCCATGATCGCCGACA
251		6	160,897,158	A/G	A	CTTTTCATCCTCTCTAGCTCTAGGGAGCTACTACTGGTTAGATGCAAAAG[A/G]TCTCTCTGGAGAATTGAAAGCATTAACCATCTGAATACTGAAGCGCGAAT
252		6	160,898,194	A/G	G	GATCAGTTTAATTAGCTCGCTCCTCCTCCTGCGTTTCCTCCGTATCTTGC[A/G]AATTCTTATCAAGATTATGCCGTCGTTTTCCGTTTCTGCCGTCGAACATT
249		6	160,987,729	A/G	G	CTGTTAAATATACGGCCTCATCGACCCTAGAGGTCAAGACGAGCAATTTAATATTCCGCT[A/G]ATGTGTTTCTTTCATGGTAATTAGAGTGACCTCCTCCATCTATTAGCTGATGTCCTGACC
254		6	161,016,898	A/C	C	TGCCTGGAGCCTTGACTGCGCAAGCGCAGCTGCCGTGTGTTGAGTGCTGAGTCGCCGACN[A/C]CACCAGGTACGGCCCGACTGCCGGTACACTCTGTGTGGTATTCTTTTCAATTTCTCTTGA
175	SYN23632	6	161,017,113	A/G	A	GCCAAAAAAAAACACGAAGAATTAAACATCGATTGAAAGGGAAAAGACAATGCCAAGCCT[A/G]AACTGAGAGGCGCACACTGGATTTCGCNTCAGTTCATGTCGTTCCTTACACCGTCAATTT
253		6	161,017,141	A/G	A	TCGATTGAAAGGGAAAAGACAATGCCAAGCCTNAACTGAGAGGCGCACACTGGATTTCGC[A/G]TCAGTTCATGTCGTTCCTTACACCGTCAATTTGACGAATCACCTCGCCACGCCGCCTTGA

269	SYN14803	6	161,634,808	T/C	T	CTTCTGCTGTGGGTGCTGATGACAAGAATGGTACCTGGGACACGACAACAAAGGGCACTC[T/C]ATCTAACGAAAAGGCTGACGACCCTTGGAATAGTAAGCGGGGTAATGATGATGACAGCAA
193	SYN30204	6	162,207,632	A/C	A	ACGGTGGGTCTGTGTTGCGACTTGTCTGTGTGTGCTATACGACCTGCCAAGTGGCAAGAG[A/C]GCCAACGCCGTCTCCTTGCCATGGCCATCCTGAACAGAGCTGACACTAGTCACACTACCA
203	PZE-106109657	6	162,468,362	A/G	A	TTAGTCACAAACACACAAGGTTTTAGTACCAAACCGCTTTGCTGCACCAG[A/G]CCATCAGGCTGTTCATCCGATCAGACTGATGAGGGCCTACCAGCCATGGC
209	PZE-106110286	6	162,597,984	A/G	A	ATAAGTTGTCAACCTTTTGCGACCAGAAACCTTTATGCCGTTATTAGACA[A/G]ATGATTAACGATCCTATGTGTTTTTCCTGCTCCAATGAGCTGCAAGCAGC
218	PZE-106110981	6	163,230,476	A/G	G	GCCGTAGCACTCACTACTGTGTGTCTGCCTTTGTCCTGCGAGAAGGAGAG[A/G]TTGGAGTATAGTGTTTGAAAGCTCGGTGGGACTCGGACGAGCCCAAGTCC
462		6	160,587,319	A/G	G	CTAGATTCTAGCAAGGAGGAGGAAGACGACCCTGTCGCCAGGTCGCGGACC[A/G]TCCGGCCCAGAGCTGCGGACCGTCCGGTGTGACGCAGGGAAGACACCGC

Table S5. Genome sequencing of tropical maize inbred lines. Sequence assembly, genome optical mapping assembly and hybrid scaffolding results are shown along with the names of the inbred lines.

The whole genome sequence is available at: https://data.cimmyt.org/privateurl.xhtml?token=53088e0e-f74c-4f5b-a435-e5a327c289d2

PacBio sequencing

Genetic Material	Total Cells	Total Number of Reads (bp)	Total Length (all cells) (bp)	Average read Length - all cells (bp)
CML543	2	13,105,110	224,813,481,042	17,155
CML536	13	9,605,682	184,998,764,067	19,259
CKL05004	4	16,558,388	319,494,240,661	19,295
CKL05022	4	18,150,734	316,953,300,304	17,462
CKDHL0186	2	63,155,497	886,658,385,041	14,039
KS23-6	24	13,305,952	188,738,959,941	14,185

Illumina Sequencing Chromium 10X sequencing output (PE150)

Sample Name	Paired End Clusters	Single Clusters	Total Clusters	Total Bases
MLN_KS23-6	683,680,068	2,668,869	686,348,937	208,095,874,776

Sequence assembly, Genome optical mapping assembly and Hybrid scaffolding

Input molecule stats (unfiltered)	CML543	CML536	CKL05004	CKL05022	CKDHL0186	KS23-6
Total number of molecules	10,904,985	12,857,942	3,821,615	8,299,095	38,259,765	9,785,131
Total length (Mbp)	862,295.26	1,025,727.39	481,735.35	833,659.22	2,866,808.52	924,092.62
Average length (kbp)	79.073	79.774	126.055	100.452	74.93	94.438
Molecule N50 (kbp)	127.875	136.5	279.75	179.625	131.625	153
Label density (/100kb)	15.384	15.744	16.238	15.653	13.579	15.019

**Input molecule stats (filtered)**						
Total number of molecules	1,307,118	1,588,181	867,462	1,666,346	1,068,690	1,573,035
Total length (Mbp)	340,522.75	463,220.32	302,341.07	459,069.79	385,113.21	426,336.33
Average length (kbp)	260.514	291.667	348.535	275.495	360.36	271.028
Molecule N50 (kbp)	265.24	306.799	388.983	287.022	362.791	276.617
Label density (/100kb)	16.362	16.561	16.475	16.332	14.826	15.972
Coverage of the reference (X)	159.552	217.041	141.662	215.097	180.444	199.759
**Bionano DLS Map statistics**						
Count	83	96	108	187	113	37
Min length (Mbp)	0.244	0.365	0.446	0.278	0.094	0.885
Median length (Mbp)	1.605	1.831	1.875	2.49	0.755	49.798
Mean length (Mbp)	26.211	23.572	21.17	13.901	19.537	58.111
N50 length (Mbp)	97.256	107.109	97.69	99.492	99.158	98.995
Max length (Mbp)	199.454	239.753	197.779	203.658	198.085	201.864
Total length (Mbp)	2,175.53	2,262.95	2,286.38	2,599.49	2,207.70	2,150.12
Average confidence	32.5	38.2	40.9	36.2	43.2	36.1
**PacBio CLR contig statistics**						
Count	903	944	1,408	800	1,511	1,134
Min length (Mbp)	0.06	0.06	0.06	0.06	0.06	0.03
Median length (Mbp)	0.098	0.099	0.103	0.113	0.166	0.063
Mean length (Mbp)	2.471	2.413	1.654	2.778	1.59	1.928
N50 length (Mbp)	50.167	70.954	45.858	49.133	13.273	21.201
Max length (Mbp)	130.208	168.092	153.368	113.139	65.625	64.024
Total length (Mbp)	2,231.25	2,278.13	2,329.08	2,222.60	2,402.94	2,186.91
**Sequence in hybrid scaffold**						
Count	188	242	356	262	275	279
Min length (Mbp)	0.007	0.003	0.003	0.003	0.066	0.001
Median length (Mbp)	0.578	0.28	0.333	0.315	4.54	2.393

Mean length (Mbp)	11.407	9.05	6.172	8.203	7.9	7.561
N50 length (Mbp)	50.167	71.847	49.38	49.106	14.424	21.48
Max length (Mbp)	130.205	168.074	153.301	113.139	65.625	63.997
Total length (Mbp)	2,144.48	2,190.16	2,197.11	2,149.08	2,172.60	2,109.41
**Hybrid scaffold statistics**						
Count	65	62	73	61	36	34
Min length (Mbp)	0.17	0.153	0.075	0.119	1.337	0.727
Median length (Mbp)	6.045	4.981	6.729	12.576	34.666	53.564
Mean length (Mbp)	33.042	35.652	30.704	35.96	60.48	62.908
N50 length (Mbp)	97.164	115.436	97.465	100.997	108.272	98.326
Max length (Mbp)	199.117	239.92	197.509	204.817	239.175	201.537
Total length (Mbp)	2,147.70	2,210.44	2,241.37	2,193.54	2,177.28	2,138.86
**Hybrid scaffold plus non scaffolded**						
Count	852	847	1,275	706	1,282	1,005
Min length (Mbp)	0.001	0	0	0	0	0
Median length (Mbp)	0.089	0.089	0.088	0.089	0.142	0.052
Mean length (Mbp)	2.623	2.714	1.861	3.211	1.878	2.205
N50 length (Mbp)	97.164	112.902	97.465	99.604	100.826	98.326
Max length (Mbp)	199.117	239.92	197.509	204.817	239.175	201.537
Total length (Mbp)	2,234.47	2,298.42	2,373.34	2,267.06	2,407.62	2,216.36

Table S6. Vectors used to transform maize lines for editing sub-regions of the 105 kb genetic interval. CR2, CR3-4, CR8, and CR13-14 were used initially, then CR8, CR8.1 and CR15 were used to edit additional inbreds.

Gene of Interest	Modification	CRISPR (1) NAME	CRISPR (1) SEQ	CRISPR (2) NAME	CRISPR (2) SEQ	Target	Expected Deletion Size
ZM-6.130-MLN-eIF1A	SDN1 - Generate variation at MLN QTL on chr6	ZM-6.130-MLN-CR2	GATCAAAAGCGTGATCAGCT			CML536	
ZM-6.130-MLN-eIF1A	SDN1 -Create eIF1A gene drop out at MLN QTL on chr6	ZM-6.130-MLN-CR3	GGTTTAGGCTGTGCCCAGTG	ZM-6.130-MLN-CR4	GTAATCAGGAACACTCGCCA	CML536	2846 bp
ZM-6.130-MLN-Peptidase	SDN1 - Generate frameshift in peptidase gene	ZM-6.130-MLN-CR8	GAATTGACCAAAGGCATGGA			CML536, CML543, CKL05004	
ZM-6.130-MLN-Intragenic	SDN1 - Create intergenic drop out at MLN QTL on chr6	ZM-6.130-MLN-CR13	GGTGGCGGCCTGCGACAGGT	ZM-6.130-MLN-CR14	GGGTCCTGGTATTCGACTCA	CML536	39885bp
ZM-6.130-MLN-Peptidase	SDN1 - Generate frameshift in peptidase gene, alternate guide for CKL05022	ZM-6.130-MLN-CR8.1	GAATT**T**ACCAAAGGCATGGA			CKL05022	
ZM-6.130-MLN-Peptidase	SDN1 - Generate frameshift in peptidase gene	ZM-6.130-MLN-CR15	GACGGTTGGTGATACGTCT			CML536, CML543, CKL05004, CKL05022	

Table S7. Primers for gene-edited events molecular characterization (GEMC).

Edit goal	Guide	Type	Germplasm	Name	Sequence	Length
Generate variation in 5′ UTR of eIF1A	CR2	SDN1	CML536	ZM-6.130-MLN_CR2_FP	AAATATTTCCGGCCTGTGTAGC	22
				ZM-6.130-MLN_CR2_RP	TCGAGATCAGACATGAAACTTGG	23
Knock out eIF1A gene	CR3+CR4			ZM-6.130-MLN_CR3_FP	CAACCGAGTTTAAGCGAACG	20
				ZM-6.130-MLN_CR3_RP	CGCTGAAAACATTGGACAGG	20
				ZM-6.130-MLN_CR4_FP	TGAGCTCGCTATTGGAGTGG	20
				ZM-6.130-MLN_CR4_RP	TGGGGCACGGATGTTCTTAC	20
Create frameshift in peptidase gene	CR8			ZM-6.130-MLN_CR8_FP	TGCTGCCTTTCTGTGTTAATGG	22
				ZM-6.130-MLN_CR8_RP	TCATTACTGATGGCTGACATGC	22
Create intergenic drop out (39.9kb)	CR13+CR14			ZM-6.130-MLN_CR13_FP	CACTACCAGGTGATCTGC	18
				ZM-6.130-MLN_CR13_RP	TGATGGTCACCACCTATTTG	20
				ZM-6.130-MLN_CR14_FP	TTCTGTCCGTCAGTCTTCTGC	21
				ZM-6.130-MLN_CR14_RP	GGCCAGCTTCATACCTCATAGC	22
Create frameshift in peptidase gene	CR15		CML536, CML543, CKL05004, CKL05022	ZM-6.130-MLN-PEPT_CR2_FP	CCAAGGTTCCAAGAGATGAGC	21
				ZM-6.130-MLN-PEPT_CR2_RP	ATTGCATATCACCCCGTTCG	20
Create frameshift in peptidase gene	CR8		CML543, CKL05004	ZM-6.130-MLN_CR8_FP	TGCTGCCTTTCTGTGTTAATGG	22
				ZM-6.130-MLN_CR8_RP	TCATTACTGATGGCTGACATGC	22
Create frameshift in peptidase gene	CR8.1		CKL05022	ZM-6.130-MLN-PEPT_CR1_FP	TGCTGCCTTTCTGTGTTAATGG	22
				ZM-6.130-MLN-PEPT_CR1_RP	TCATTACTGATGGCTGACATGC	22

Table S8. PCR primers and assays to distinguish edited variants of four elite inbred lines from sub-Saharan Africa.

Trait	dPCR Platform	Forward Primer	Reverse Primer	Probe	Amplicon Size	Quencher	Genotype	Guide	Assay notes
MLN_Variant_AC	QIAcuity	AATGCTGATTAGTTTTGTTGTCCGT	TTGAACGACCCCCAAGAAAAC	TACGGCATGTATATAAC	123	MGB			To distinguish CKL05022 flanking sequence from others
MLN_Variant_GT	QIAcuity	AATGCTGATTAGTTTTGTTGTCCGT	TTGAACGACCCCCAAGAAAAC	TACGACATGCATATAAC	123	MGB			To distinguish CKL05022 flanking sequence from others
ZM-6.130-MLN_001	QIAcuity	CCCACGAGGCAAGAATTTTG	CTGGTAAACATCTTTCTGTCTTTATTTTCT	CCTTCCTGCCTTTGGT	122	MGB	CML536	MLN-CR8	
ZM-6.130-MLN_002	QIAcuity	CCCACGAGGCAAGAATTTTG	CTGGTAAACATCTTTCTGTCTTTATTTTCT	CCTTCATGCCTTTGGTC	122	MGB	CML536	MLN-CR8	
ZM-6.130-MLN_003	QIAcuity	CCCACGAGGCAAGAATTTTG	CTGGTAAACATCTTTCTGTCTTTATTTTCT	AGCGCCTTTGGTCAA	114	MGB	CML536	MLN-CR8	
ZM-6.130-MLN_004	BioRad	TGTTAATGGGTTCATCAGCAGC	CTGATGGCTGACATGCACTGT	CGCCTTCCAGCCTT	169	MGB	CKL05022	MLN-PEPT-CR8.1	
ZM-6.130-MLN_005	QIAcuity	TGTTAATGGGTTCATCAGCAGC	CTGATGGCTGACATGCACTGT	CAATGCCTTTGGTCAATT	171	MGB	CKL05022	MLN-PEPT-CR8.1	
ZM-6.130-MLN_008	BioRad	TGTTAATGGGTTCATCAGCAGC	CTGATGGCTGACATGCACTGT	CCTTCCCCCTTTGGTCA	168	MGB	CKL05004	MLN-CR8	
ZM-6.130-MLN_009	QIAcuity	TGTTAATGGGTTCATCAGCAGC	CTGATGGCTGACATGCACTGT	ATCTATTAAATTGGAAGGCGC	158	MGB	CML543	MLN-CR8	
ZM-6.130-MLN_010	QIAcuity	TGTTAATGGGTTCATCAGCAGC	CTGATGGCTGACATGCACTGT	CCTTCCAAGAAAATAAAGACAG	120	MGB	CML543	MLN-CR8	

ZM-6.130-MLN_101	BioRad	TCCAAGGTTCCAAGAGATGAGC	CTGGTAAACATCTTTCTGTCTTTATTTTCT	CCAAGACACCAACCGT	163	MGB	CKL05022	MLN-PEPT-CR15	
ZM-6.130-MLN_102	BioRad	TCCAAGGTTCCAAGAGATGAGC	CTGGTAAACATCTTTCTGTCTTTATTTTCT	CCCAAGAGCGTATCACCAA	169	MGB	CKL05004	MLN-PEPT-CR15	
ZM-6.130-MLN_103	BioRad	TCCAAGGTTCCAAGAGATGAGC	CTGGTAAACATCTTTCTGTCTTTATTTTCT	CCCAAGAACAAGAACAA	152	MGB	CKL05004	MLN-PEPT-CR15	
ZM-6.130-MLN_104	BioRad	TCCAAGGTTCCAAGAGATGAGC	CTGGTAAACATCTTTCTGTCTTTATTTTCT	AATACCTGAAGCCCAACTG	140	MGB	CML536	MLN-PEPT-CR15	
ZM-6.130-MLN_105	BioRad	TCCAAGGTTCCAAGAGATGAGC	CTGGTAAACATCTTTCTGTCTTTATTTTCT	AGAACAATACCTGAAGCGAA	113	MGB	CML536	MLN-PEPT-CR15	
ZM-6.130-MLN_106	BioRad	TCCAAGGTTCCAAGAGATGAGC	CTGGTAAACATCTTTCTGTCTTTATTTTCT	AAGCCCAAGAGTATCACC	167	MGB	CML536	MLN-PEPT-CR15	
ZM-6.130-MLN_107	BioRad	TCCAAGGTTCCAAGAGATGAGC	CTGGTAAACATCTTTCTGTCTTTATTTTCT	CCCAAGAACGTATCACCA	169	MGB	CML543	MLN-PEPT-CR15	
ZM-6.130-MLN_108	QIAcuity	TCCAAGGTTCCAAGAGATGAGC	CTGGTAAACATCTTTCTGTCTTTATTTTCT	AAGCCCAAGATATCACCA	166	MGB	CML543	MLN-PEPT-CR15	

Table S9. Sequences used to assemble vectors for subcellular localization of peptidase and various fusion proteins.

Product	Sequence Name	Sequence	Calculated Molecular Weight	Normalization	Quantity	fm/ng	Sequence Length	Sequence start-end
gBlocks™ Gene Fragments	Peptidase 1	GGACTCAGATCTCGAGAGGCCCAGGAGATCGCC GCTGCAGCGCGCCACTTCTGCGCCATGGTTCGA ATCGTCGGTCCGGACCCCAAGGCCGTGAAGATG CGCCGCCACGCCTTTCATTTTCACCATTCGGGG TCGACCACGCTCTCGGCGTCGGCTCTGCTCCTG CCGCGGGGCGCTTTAGCTGAGCCGCCGCCATTT CTCGACCATATCTGCTCGGCCCACGGGCACACG GCAGGAGACGTCGCGCTCACGGCTGCTTCCCTC GTCGAGCCGTTCCTGGTCGCGGAGCAGCGCAAT AACTCCGGCGAGGAGCTTCAGCCGAGGTTGGTT CCGGAGACGCGTCTTGATGTGTTTGTTGAGTAT GAGTTGGGGAATGCTCAAGATGGGAAGTCTGGA CCTCCGCGGTGGCTTCCAGCTCGACTGCTTGCC ATGGTTGATGTCCCAACAGCTGCTGTTTCTGCT TTATCCTTATTGAGACATGACGATTCATTCATC AGAAG	308876.65	500ng = 1619fmol	500 Nanograms	3.24	500	1-500
gBlocks™ Gene Fragments	peptidase 2	GATTCATTCATCAGAAGGCCAACTTGGGATGTA GGCTGGTCATTGGCTGATGCTAATCAGAAACAG GTCTCTTTGTTCATCGAATCAAAATCTTCCCTC GAGTCTAACAGGAATAATTCATCTTTGGAGTCA GTAGACTCATTGATGTTGGCCAAGTCTGCCACA AGAATTGCTATTCTAGGAATTTCAACCTCCAAT TTAAATGATGCAAGACGTATCAATGTTTCAGTG ATGCAACAACGAGGGGACCCTTTGCTTATAGTA GGATCTCCATTTGACCTCATGTCACCCTTCCAT TTCTTCAACAGTGTATCAGTTGGTGCTGTTGCA AATTGCCTTCCTCCATGCACTGCAAGGAGCTCA TTACTGATGGCTGACATGCACTGTCTCCCTGGC ATGGAAGGCGCTCCAGTGTTTGACCAAAATTCT TGCCTCGTGGGGCTGCTGATGAACCCATTAACA CAGAAAGGCAGCAATATAGAAGTCCAGCTCGTG ATTAC	308798.67	500ng = 1619fmol	500 Nanograms	3.24	500	501-1000

gBlocks™ Gene Fragments	peptidase 3	CCAGCTCGTGATTACATGGGATGCAATATGCAC<br>GGAATGGAACAGCAAAAAACTGGAGGAAATTGA<br>ACGACCCCCAAGAAAACTACCTAATGACAAAAA<br>TACAGATAGTAAATCTATGGAATTACGGCATGT<br>ATATAACTATGTGAGGGTTTTCTCTTCTACGGA<br>CAACAAAACTAATCAGCATTGCATATCACCCCG<br>TTCGCTCAGAGAGGCTATATCTGCAGTTGTTCT<br>TGTCACGGTTGGTGATACGTCTTGGGCTTCAGG<br>TATTGTTCTGAACAAAAGGGGTTTAGTTCTGAC<br>AAATGCTCATCTCTTGGAACCTTGGAGATTTGG<br>AAGAACTTCACCTTCAGATTTACAAGCCTCGTT<br>CGCTGGAGAACATCTCAATGCTGGAGAAAACAA<br>ATCATTGCAACCACAACAAGGCAAAATTTCCAA<br>TGAAGATGCTGTCAAGCATAAGGTTTCGTCATT<br>TAACTTGGGTTTCAAAAGAGGGAAGAGAATATC<br>TGTTC	308780.91	500ng = 1619fmol	500 Nanogram s	3.24	500	1001-1500
gBlocks™ Gene Fragments	peptidase 4	GAAGAGAATATCTGTTCGTTTGGACCATGAAGA<br>GAGACAGATATGGTGCAATGCTAGTGTGGTTTT<br>CATCTCAAAGGGTCCACTTGATGTTGCATTGCT<br>TCAAATAGAAAAGGTTCCAGTTGAATTAAATAC<br>AATCAGACCAGAATTTGTTTGTCCAACAGCAGG<br>GTCGCCTGTTTATGTTGTTGGGCATGGCCTTTT<br>TGGACCCCGATCAGGCCTACACTCTTCTCTATA<br>CTCGGGGGTTGTGTCAAAGGTTGTCCAAATCCC<br>AGCAAATCAACTTTCTCATCTGGCCCGTGCTGA<br>GGCTGACAATATGGACATACCAGTAATGCTTCA<br>GACAACAGCAGCAGTTCATCCAGGAGCCAGTGG<br>CGGCGTTCTTGTTAATACACATGGGCTAATGGT<br>TGGGATAATAACAAGTAATGCTAAGCATGGTGG<br>TGGAAGCACAATACCTCATCTGAATTTCAGCAT<br>CCCCTGCAAATTACTGGTTGCAGTCTTCGAGTA<br>TTCAG	308799.66	500ng = 1619fmol	500 Nanogram s	3.24	500	1501-2000
gBlocks™ Gene Fragments	peptidase 5	GTCTTCGAGTATTCAGCAAATGGAAACCTCGTG<br>GTTTTGGAGCAGTTGGACAAACCAAATGAAGTG<br>CTCTCATCAGTTTGGGCATTGGCACCATCATCA<br>TCCCCATTTGTCAGGAGCTCCCCAGAAAAAGGC<br>AAAGAGGAAAAGGTCTTGGAGTTCTCTAAGTTT<br>CTTAGTGACAAGCAACAAGCTCTGAAATCTAAC<br>GTAGATCTGAAGGAACTATTTAGGTACAAGACT<br>CCCAGCAAAATATAGGGTACCAATGGCCAGTTA<br>ACAG	165456.09	500ng = 3022fmol	500 Nanogram s	6.04	268	2001-2268

RED BackBone forward primer	TAGTAGGGCCCAGAAGTGAGTTAACCTACT
TAG RFP SKL reverse primer	CTTCTGGGCCCTACTACAGTTTGCTGTTCAATTTGTGACCTAGCTTGGAAGG
TAG RFP SKI reverse primer	CTTCTGGGCCCTACTATATTTTGCTGTTCAATTTGTGACCTAGCTTGGAAGG

Reverse peptidase backbone	CCATGGATCCTAGGCTAAGTTAAAGTCG
Kan forward	CAGCGATCGCGTATTTCGTCTCG
Reverse Kan	GAAATACGCGATCGCTGTTAAAAGG
Linker peptidase for	GTGGAGGCGGCAGCGGTGGCGGAGGCTCCGGAGGCGGTGGCGAGGCCCAGGAGATCGCCG
**RV020150 AC-GFP1 amplified PCR primers**	
Rev ac-gfp linker1	GCTGCCGCCTCCACCCTTGTACAGCTCATCCATGCCG
For ac-gfp	AGCCTAGGATCCATGGTGAGCAAGGGCGCC
KAN For	GTCCTTTTAACAGCGATCGCGTATTTCGTCTC **+ TAGRFPSKI**
REV	CTTCTGGGCCCTACTATATTTTGCTGTTCAATTTGTGACCTAGCTTGGAAGG
KAN For	GTCCTTTTAACAGCGATCGCGTATTTCGTCTC **+ TAGRFPSKL**
REV	CTTCTGGGCCCTACTACAGTTTGCTGTTCAATTTGTGACCTAGCTTGGAAGG
RED BackBone for	TAGTAGGGCCCAGAAGTGAGTTAACCTACT
REV linker GFP	GCTGCCGCCTCCACCCTTGTACAGCTCATCCATGCCG
For Linker Peptidase	GTGGAGGCGGCAGCGGTGGCGGAGGCTCCGGAGGCGGTGGCGAGGCCCAGGAGATCGCCG
KAN rev	GAAATACGCGATCGCTGTTAAAAGG

Supporting Information Appendix

Genomic, mRNA, and amino acid sequences for peptidase from the inbred lines CML536 and KS23-6

Targeted knockout of a host peroxisomal peptidase confers field resistance to maize lethal necrosis

Jung et al.

dhuggaks@gmail.com

```
>CML536_Peptidase_Genomic_DNA
ATGACTTCTTTTACTGCACGGGAGTACAGCTCTCGTCGGGGTCTTCTCCCCCTTCGGCCCTTCCCCCCTCCCTGCGCGCC
ATGGAGGCCCAGGAGATCGCCGCTGCAGCGCGCCACTTCTGCGCCATGGTTCGAATCGTCGGTCCGGTTAGTCTGCGACT
TCGCACCATCGGCAGCTCACCCGTATTATCTTGCTAACCGCCGTGCTTCTCCACTTCTCGGGGCTCCGGCATCTCCGACT
CCAGGACCCCAAGGCCGTGAAGATGCGCCGCCACGCCTTTCATTTTCACCAGTTAGCCCTCACCCCTCCCAATTTTGAGA
TTTTTACTCGATATAAAATCGAGCTTTCCTAAAGCCCCGCGCTGACTTGGGGCTGCAGTTCGGGGTCGACCACGCTCTCG
GCGTCGGCTCTGCTCCTGCCGCGGGGCGCTTTAGCGGAGCCGCCGCTACTTCTCGACCATATCTGCTCGGCCCACGGGCA
CGCGGCAGGAGACGTCGCGCTCACGGCTGCTTCCCTCGTCGAGCCGTTCCTGGTCGCGGAGCAGCGCAATAACTCCGGCG
AGGTTGGTAGGTTACAGTGTCACAGCACCGTGTAATCACTCTTTAGTGCTAAGATTTTGTTTGCTTTTTCTTTGGATGCC
TGCAGGAGCTTCAGCCGAGGTTGGTTCCGGAGACGCGTCTTGATGTGTTTGTTGAGGTAAAGATAATAAGGGCCTGTTTG
GGAATACAGTTTTGAAATACTGTAGTTTTGAGATAGCATAGTTTACAATTGTACATGACATAAATACTACGGTATTGCTT
TACCACAGTAAAACTACAGTATTGCTCAAAACCGAGATCTGTTTGGTTCTATTAGAAAAACAAAGTATATATAGAGAAGA
GAAGAAAACTGAGGTCCTGAGTGGAGTTTCAAAAACTCTAAAAATACCACAGTTTTGGGTAAACCACGGTATTTAAAATT
GAGTTTTGACTGTACAAACCAAACATCTTTTGAGCTCCAATACTATAGTATCATTAAATACCATAGTATTGTTTCAAAAC
CGCAAAAATACTACAATTCCAAACAGGGCCTAAAGCATCGGTGTGGAATTCGTGCTTAGCATTCAATCATTCATGATCAG
TAAAGTGCCTCTTTGCAAAGGAAGTTAAGTGAACTCAAATTGATTATTGCTGGAAGTGCATCAGTTACATCCCGATTCCG
AACTATATATAGCTAATAAACCAATGCATGATGTACAATGTGTGTCCTCGTACCGTAAGGAACTCATGCCTTCCAAGATA
TTAGTTTCAAAACCAGGAAGTTTTTCATGAATTCCTGAACCTTCTGTGATGTGTACAGTATGAGTTGGGGAATGCTCAAG
ATGGGAAGTCTGGACCTCCGCGGTGGCTTCCAGCTCGACTGCTTGCCATGGTCAGTACTGTAATTTTTTCATCTAGATTG
TAGTGTTACTAATAGGTACAGGAAAAAGTAGCAGCCAGGGAGTTAGTTTATACTGTTAATAACAGATGCACGAAAATAGC
AGCCATTATGTTACAAGATAAAGGTTGTTTTCTGAGTCCATTTAGTTTGTGAGGATGTTCTATGTTCAGAAATAGTGGTG
ATGCCCAATTGGACTTAAAAGTTTTACACACTATGTGCATTTTACTTTCAGTTTAAGGAGTTTTGCAACTGTGCATTGCA
GCAGCCTTTATCGGTCTTGATTAAACTATGATTCCAAAATGTTTTTTTGCCAGTGTTCTGAAACTGTTTAAACCTGTAGT
AGTTGCTATCAATTTATTTTCGGTAAGAAGAGTTGCTTAACTTGCTATTGACATCGTACCAATGACCTATATTAAGAATT
TAAGACTGGCCTTGGTTGCTCATTAGGAATAAACTAAGGCCAGTAAGAAAGAGGGAATTTGTTTAGATATCCTTATTACG
AGTAGAAGGACAAGTCTGGTGCAGTGGTGAGACCTGCCTCACTGAGTCGCCAGGTTGTGGTTCGAAGCAGCCTCTTGCAT
TTGCAGGGAAAGTCTTGCTTTGGTTGATCTCTTCTGCAGACCCCACTCACGTGGGAGCCTTCGGCACTGGGTCTGCCTTT
TTTATTTAGTGATCTGCATATGTACACTGCACTCAGCAGAAGAAAGTACACTTCTGTCTTGTAGTAACATAACATTTGTA
GGTTTTGGTTAGTCTTCACCCACGCATACATGATCAACCAGCAGCCTGGTTTCTTCTTTAGGTTGATGTCCCAACAGCTG
CTGTTTCTGCTTTATCCTTATTGAGACATGACGATTCATTCATCAGAAGGCCAACTTGGGATGTAGGCTGGTCATTGGCT
GATGCTAATCAGAAACAGGTCTCTTTGTTCATCGAATCAAAGTATTAAACTGTTGTCCCTCTTTTGTCTTGCATTAGAAC
TTACCGTTACAATTTACAAGGTTGAAAATGACAGCAGATCTTCCCTCGAGTCTAACAGGAATAATTCATCTTTGGAGTCA
GTAGACTCATTGATGTTGGCCAAGTCTGCCACAAGAATTGCTATTCTAGGAATTTCAACCTCCAATTTAAATGTAAGGCA
CATGTCGATATATTTTTCTTTTCCTGAAAATTAGCACTTTGAAAGTTACAAGCTGTAGTTTTTTTGTACTGAGTATTCTT
TGGTCGTTGGTGAACAGGATGCAAGACGTATCAATGTTTCAGTGATGCAACAACGAGGGGACCCTTTGCTTATAGTAGGA
TCTCCATTTGGCCTCATGTCACCCTTCCATTTCTTCAACAGGTAATGTTTATTCTACTGCTTTAGCAGAATGCCAGCATG
TCTTTAACTCTTTAAGTATGCACCAGAATTTAGTGCATGTAACCTGTCGAACATTTTGATTTGATGATGACAAGAACATA
GTCTGGGCATTCATAATGTTGACTTATGCAATGGTAAACTCCAGATGTTTTAAAACTTGATACTGTTCCATATGCATTAT
GGAGAAAACACTAAACTTTTAATATCTAGAGTTTTATTAACTCTTTGCTTTGCACGTCATCATTACATCTTCCAAAGCAT
ATACATTTGTTGTGTTGTGTTTGTTCCCGGTATGTCTAGTGGCCTGGAAAGCGTTAACTAGATACTGATGTTTCTAAAAT
TGAAAACGCCTGGCATGCTTTTGTTGACTATTTTCTTGTGCTACCATTATTAGAAAACAGAATAAGAGTGTCATATTTAT
TTGTATACATATGTGTTTTTTGAGCAGTGTATCAGTTGGTGCTGTTGCAAATTGCCTTCCTCCATGCACTGCAAGGAGCT
CATTACTGATGGCTGACATGCACTGTCTCCCTGGTAAACATCTTTCTGTCTTTATTTTCTTGTATGGTATGACATTAGTT
ATTGATAATCTATTAAATTGACCAAAGGCATGGAAGGCGCTCCAGTGTTTGACCAAAATTCTTGCCTCGTGGGGCTGCTG
ATGAACCCATTAACACAGAAAGGCAGCAATATAGAAGTCCAGGTTAGTTCACTGGTTTCAACAAGATACATTTGCACATC
GAATATTTGTAACTTAATTATCTTAAAATCTACTTACCAGCTCGTGATTACATGGGATGCAATATGCACGGAATGGAACA
GCAAAAAACTGGAGGAAATTGAACGACCCCCAAGAAAACTACCTAATGACAAAAATACAGATAGTAAATCTATGGAATTA
CGGCATGTATATAACTATGTGAGGGTTTTCTCTTCTACGGACAACAAAACTAATCAGCATTGCATATCACCCCGTTCGCT
CAGAGAGGCTATATCTGCAGTTGTTCTTGTCACGGTTGGTGATACGTCTTGGGCTTCAGGTATTGTTCTGAACAAAAGGG
GTTTAGTTCTGACAAATGCTCATCTCTTGGAACCTTGGAGATTTGGAAGAACTTCACCTTCAGATTTACAAGCCTCGTTC
GCTGGAGAACATCTCAATGCTGGAGAAAACAAATCATTGCAACCACAACAAGGCAAAATTTCCAATGAAGATGCTGTCAA
GCATAAGGTTTCGTCATTTAACTTGGGTTTCAAAAGAGGGAAGAGAATATCTGTTCGTTTGGACCATGAAGAGAGACAGA
TATGGTGCAATGCTAGTGTGGTTTTCATCTCAAAGGGTCCACTTGATGTTGCATTGCTTCAAATAGAAAAGGTTCCAGTT
GAATTAAATACAATCAGACCAGAATTTGTTTGTCCAACAGCAGGGTCGCCTGTTTATGTTGTTGGGCATGGCCTTTTTGG
ACCCCGATCAGGTGAAAAATCTTAGAACCGCAGCTTAAATTAGTGTTTGAGCAGTTCGATGTTTTTTTTTATCAATGTGC
ATGGCTGCATGCCATTTCAAATGCTGAGTTTTGTTATTATGGAACTGATGTTTGAGTATCTGAATTTAGGCCTACACTCT
TCTCTATACTCGGGGGTTGTGTCAAAGGTTGTCCAAATCCCAGCAAATCAACTTTCTCATCTGGCCCGTGCTGAGGCTGA
```

CAATATGGACATACCAGTAATGCTTCAGACAACAGCAGCAGTTCATCCAGGAGCCAGTGGCGGCGTTCTTGTTAATACAC
ATGGGCTAATGGTTGGGATAATAACAAGGTATAAAATAGAACCTTTTTGAAGCCTTACATACGTTAACTAGAACCTAAAG
TTCATCAAGTTATTTTGCCATTTCATCGACTGTATCAACTTTCTACATTTTATGTTGTTATGTGTCATTTTATGATATGC
TAAAACATTGCAAGCATAGCATCATGTCATTCATTTTCGTAACTAGAATAATGGTGAAGTATGTATTTTCCAAAGAGCTT
GTCTTTTTTCTGCCTTATTTTTTTTATTACCATATCCAGTAATGCTAAGCATGGTGGTGGAAGCACAATACCTCATCTGA
ATTTCAGCATCCCCTGCAAATTACTGGTTGCAGTCTTCGAGTATTCAGGTGCTAATTAAGTTTGCTCGTATTTTTTCTGC
TCTCAGCTTTAGTCAATATTGATGGTATCAAAACCCTTCTAAGTACATATAAAAATAAGGAATGCATTTCCTTTCTTGGC
AGCAAATGGAAACCTCGTGGTTTTGGAGCAGTTGGACAAACCAAATGAAGTGCTCTCATCAGTTTGGGCATTGGCACCAT
CATCATCCCCATTTGTCAGGAGCTCCCCAGAAAAAGGCAAAGAGGAAAAGGTCTTGGAGTTCTCTAAGTTTCTTAGTGAC
AAGCAACAAGCTCTGAAATCTAACGTAGATCTGAAGGAACTATTTAGGTACAAGACTCCCAGCAAAATATAGAGTGCGTC
CACTAGAATTCATACAAGGAAAATGTTTGTTCCCAGAATTTTGTATGAGACGTTTGATCCCAGAAATCCCCAGTACAAAA
TGAGTTATCAGAGCACAGTGCCTTTTTTTTACAGATGGTTAAGGCATAAGCATCTTTTCTTCGAGTGTCAGGTAGCGCGG
GCTATATGGCATACTGTTCAAATCGCTACAATTTTTGCCCTCCCTCTAGTGTCGTGAATATGTTTAGGTCATAGCTAGGG
GGATTGGTAAAGATTTAAAATTGTTAGCTCTTTTAGGGGCAGCAGCAGTTTGTTGGGCCATTTGACATTGCGTTTCAAAG
AAAATATGTGACAAACTCTTTACAGGTTCTTCGTTTGGTTATTCACTGGCTCCGTTCCTAGGTTGTGCTACAGAAGCCTA
TCTTTCAGGAGTTGGTTTTGGCTACCTGACAACGTTTGGAGCAAATGGTCAGGTTGTTTTTTACCTAGGCTCGCGGGTGG
CAATCTAGTCTTAGGATTGATTTCTACTAGAGTTATTAGGTTGTTAGTCTTCTTTAGACTGTGTGCATCTAGCGCGATGT
AGAAGTTTTGTTGTCAAGAAAGTTGTGACTTATGGTTATCTTGATGCAATAAATTTTCTCTTCTCGAAAAAAATGATGAT
GCCGCTGGGAATGTAATAAACTTATATAGGAACAGTTTTTACAATTGAAAATGTCAATTACC

>CML536_Peptidase_mRNA
ATGACTTCTTTTACTGCACGGGAGTACAGCTCTCGTCGGGGTCTTCTCCCCCTTCGGCCCTTCCCCCCTCCCTGCGCGCC
ATGGAGGCCCAGGAGATCGCCGCTGCAGCGCGCCACTTCTGCGCCATGGTTCGAATCGTCGGTCCGGACCCCAAGGCCGT
GAAGATGCGCCGCCACGCCTTTCATTTTCACCATTCGGGGTCGACCACGCTCTCGGCGTCGGCTCTGCTCCTGCCGCGGG
GCGCTTTAGCGGAGCCGCCGCTACTTCTCGACCATATCTGCTCGGCCCACGGGCACGCGGCAGGAGACGTCGCGCTCACG
GCTGCTTCCCTCGTCGAGCCGTTCCTGGTCGCGGAGCAGCGCAATAACTCCGGCGAGGAGCTTCAGCCGAGGTTGGTTCC
GGAGACGCGTCTTGATGTGTTTGTTGAGTATGAGTTGGGGAATGCTCAAGATGGGAAGTCTGGACCTCCGCGGTGGCTTC
CAGCTCGACTGCTTGCCATGGTTGATGTCCCAACAGCTGCTGTTTCTGCTTTATCCTTATTGAGACATGACGATTCATTC
ATCAGAAGGCCAACTTGGGATGTAGGCTGGTCATTGGCTGATGCTAATCAGAAACAGGTCTCTTTGTTCATCGAATCAAA
ATCTTCCCTCGAGTCTAACAGGAATAATTCATCTTTGGAGTCAGTAGACTCATTGATGTTGGCCAAGTCTGCCACAAGAA
TTGCTATTCTAGGAATTTCAACCTCCAATTTAAATGATGCAAGACGTATCAATGTTTCAGTGATGCAACAACGAGGGGAC
CCTTTGCTTATAGTAGGATCTCCATTTGGCCTCATGTCACCCTTCCATTTCTTCAACAGTGTATCAGTTGGTGCTGTTGC
AAATTGCCTTCCTCCATGCACTGCAAGGAGCTCATTACTGATGGCTGACATGCACTGTCTCCCTGGCATGGAAGGCGCTC
CAGTGTTTGACCAAAATTCTTGCCTCGTGGGGCTGCTGATGAACCCATTAACACAGAAAGGCAGCAATATAGAAGTCCAG
CTCGTGATTACATGGGATGCAATATGCACGGAATGGAACAGCAAAAAACTGGAGGAAATTGAACGACCCCCAAGAAAACT
ACCTAATGACAAAAATACAGATAGTAAATCTATGGAATTACGGCATGTATATAACTATGTGAGGGTTTTCTCTTCTACGG
ACAACAAAACTAATCAGCATTGCATATCACCCCGTTCGCTCAGAGAGGCTATATCTGCAGTTGTTCTTGTCACGGTTGGT
GATACGTCTTGGGCTTCAGGTATTGTTCTGAACAAAAGGGGTTTAGTTCTGACAAATGCTCATCTCTTGGAACCTTGGAG
ATTTGGAAGAACTTCACCTTCAGATTTACAAGCCTCGTTCGCTGGAGAACATCTCAATGCTGGAGAAAACAAATCATTGC
AACCACAACAAGGCAAAATTTCCAATGAAGATGCTGTCAAGCATAAGGTTTCGTCATTTAACTTGGGTTTCAAAAGAGGG
AAGAGAATATCTGTTCGTTTGGACCATGAAGAGAGACAGATATGGTGCAATGCTAGTGTGGTTTTCATCTCAAAGGGTCC
ACTTGATGTTGCATTGCTTCAAATAGAAAAGGTTCCAGTTGAATTAAATACAATCAGACCAGAATTTGTTTGTCCAACAG
CAGGGTCGCCTGTTTATGTTGTTGGGCATGGCCTTTTTGGACCCCGATCAGGCCTACACTCTTCTCTATACTCGGGGGTT
GTGTCAAAGGTTGTCCAAATCCCAGCAAATCAACTTTCTCATCTGGCCCGTGCTGAGGCTGACAATATGGACATACCAGT
AATGCTTCAGACAACAGCAGCAGTTCATCCAGGAGCCAGTGGCGGCGTTCTTGTTAATACACATGGGCTAATGGTTGGGA
TAATAACAAGTAATGCTAAGCATGGTGGTGGAAGCACAATACCTCATCTGAATTTCAGCATCCCCTGCAAATTACTGGTT
GCAGTCTTCGAGTATTCAGCAAATGGAAACCTCGTGGTTTTGGAGCAGTTGGACAAACCAAATGAAGTGCTCTCATCAGT
TTGGGCATTGGCACCATCATCATCCCCATTTGTCAGGAGCTCCCCAGAAAAAGGCAAAGAGGAAAAGGTCTTGGAGTTCT
CTAAGTTTCTTAGTGACAAGCAACAAGCTCTGAAATCTAACGTAGATCTGAAGGAACTATTTAGGTACAAGACTCCCAGC
AAAATATAGAGTGCGTCCACTAGAATTCATACAAGGAAAATGTTTGTTCCCAGAATTTTGTATGAGACGTTTGATCCCAG
AAATCCCCAGTACAAAATGAGTTATCAGAGCACAGTGCCTTTTTTTTACAGATGGTTAAGGCATAAGCATCTTTTCTTCG
AGTGTCAGGTAGCGCGGGCTATATGGCATACTGTTCAAATCGCTACAATTTTTGCCCTCCCTCTAGTGTCGTGAATATGT
TTAGGTCATAGCTAGGGGGATTGGTAAAGATTTAAAATTGTTAGCTCTTTTAGGGGCAGCAGCAGTTTGTTGGGCCATTT
GACATTGCGTTTCAAAGAAAATATGTGACAAACTCTTTACAGGTTCTTCGTTTGGTTATTCACTGGCTCCGTTCCTAGGT
TGTGCTACAGAAGCCTATCTTTCAGGAGTTGGTTTTGGCTACCTGACAACGTTTGGAGCAAATGGTCAGGTTGTTTTTTA
CCTAGGCTCGCGGGTGGCAATCTAGTCTTAGGATTGATTTCTACTAGAGTTATTAGGTTGTTAGTCTTCTTTAGACTGTG
TGCATCTAGCGCGATGTAGAAGTTTTGTTGTCAAGAAAGTTGTGACTTATGGTTATCTTGATGCAATAAATTTTCTCTTC

TCGAAAAAAATGATGATGCCGCTGGGAATGTAATAAACTTATATAGGAACAGTTTTTACAATTGAAAATGTCAATTACC

>CML536_Peptidase_Protein
MEAQEIAAAARHFCAMVRIVGPDPKAVKMRRHAFHFHHSGSTTLSASALLLPRGALAEPPLLLDHICSAHGHAAGDVALT
AASLVEPFLVAEQRNNSGEELQPRLVPETRLDVFVEYELGNAQDGKSGPPRWLPARLLAMVDVPTAAVSALSLLRHDDSF
IRRPTWDVGWSLADANQKQVSLFIESKSSLESNRNNSSLESVDSLMLAKSATRIAILGISTSNLNDARRINVSVMQQRGD
PLLIVGSPFGLMSPFHFFNSVSVGAVANCLPPCTARSSLLMADMHCLPGMEGAPVFDQNSCLVGLLMNPLTQKGSNIEVQ
LVITWDAICTEWNSKKLEEIERPPRKLPNDKNTDSKSMELRHVYNYVRVFSSTDNKTNQHCISPRSLREAISAVVLVTVG
DTSWASGIVLNKRGLVLTNAHLLEPWRFGRTSPSDLQASFAGEHLNAGENKSLQPQQGKISNEDAVKHKVSSFNLGFKRG
KRISVRLDHEERQIWCNASVVFISKGPLDVALLQIEKVPVELNTIRPEFVCPTAGSPVYVVGHGLFGPRSGLHSSLYSGV
VSKVVQIPANQLSHLARAEADNMDIPVMLQTTAAVHPGASGGVLVNTHGLMVGIITSNAKHGGGSTIPHLNFSIPCKLLV
AVFEYSANGNLVVLEQLDKPNEVLSSVWALAPSSSPFVRSSPEKGKEEKVLEFSKFLSDKQQALKSNVDLKELFRYKTPS
KI

>KS23-6_Peptidase_Genomic_DNA
ATCCAAACAGCTTTCAAGGAGAAGCGTACAGTCCCTGCTATACAGCTCTCGTCGGGGTCTTCTCCCCCTTCGGCCCTTCC
CCCCTCCCTGCGCGCCATGGAGGCCCAGGAGATCGCCGCTGCAGCGCGCCACTTCTGCGCCATGGTTCGAATCGTCGGTC
CGGTTAGTCCGCGACTTCGCACCATCGGCAGCTCACCCGTATTATCTTGCTAACCGCCGTGCTTCTCCACTTCTCGGGGC
TCCGGCATCTCCGACTCCAGGACCCCAAGGCCGTGAAGATGCGCCGCCACGCCTTTCATTTTCACCAGTTAGCCCTCACC
CCTCTCAATTTTGAGATGTTTTACTCGATATAAAATCGAGCTTTCCTAAAGCCCCGCGCTGACTTGGGGCTGCAGTTCGG
GGTCGACCACGCTCTCGGCGTCGGCTCTGCTCCTGCCGCGGGGCGCTTTAGCTGAGCCGCCGCCATTTCTCGACCATATC
TGCTCGGCCCACGGGCACACGGCAGGAGACGTCGCGCTCACGGCTGCTTCCCTCGTCGAGCCGTTCCTGGTCGCGGAGCA
GCGCAATAACTCCGGCGAGGTTGGTAGGTTACAGTGTCACAGCACCGTGTAATCACTCTTTAGTGCTAAGATTTTGTTTG
CTTTTTCTTTGGATGCCTGCAGGAGCTTCAGCCGAGGTTGGTTCCGGAGACGCGTCTTGATGTGTTTGTTGAGGTAAAGA
TAATAAGGGCCTGTTTGGGAATACAGTTTTGAAATACTGTAGTTTTGAGATAGCATAGTTTACAATTGTACATGACATAA
ATACTACGGTATTGCTTTACCACAGTAAAACTACAGTATTGCTCAAAACCGAGATCTGTTTGGTTCTATTAGAAAAACAA
AGTATATAGAGAGAAGAGAAGAAAACTGAGGTCCTGAGTGGAGTTTCAAAAACTCTAAAAATACCACAGTTTTGGGTAAA
CCACGGTATTTAAAACTGAGTTTTGACTGTACAAACCAAACACCTTTTGAGCTCCAATACTATAGTATCATTAAATATCA
TAGTATTGTTTCAAAACCGCAAAAATACTACAATTCCAAACAGGGCCTAAAGCATCGGTGTGGAATTCGTGCTTAGCATT
CAATCATTCATGATCAGTAAAGTGCCTCTTTGCAAAGGAAGTTAAGTGAACTCAAATTGATTATTGCTGGAAGTGCATCA
GTTACATCCCGATTCCGAACTATATATAGCTAATAAACCAATGCATGATGTACAATGTGTGTCCTCGTACCGTAAGGAAC
TCGTGCCTTCCAAGATATTAGTTTCAAAACCAGGAAGTTTTTCATGAATTCCTGAACCTTCTGTGATGTGTACAGTATGA
GTTGGGGAATGCTCAAGATGGGAAGTCTGGACCTCCGCGGTGGCTTCCAGCTCGACTGCTTGCCATGGTCAGTACTGTAA
TTTTTTCATCTAGATTGTAGTGTTACTAATAGGTACAGGAAAAAGTAGCAGCCAGGGAGTTAGTTTATACTGTTAATAAC
AGATGCACGAAAATAGCAGCCATTATGTTACAAGATAAAGGTTGTTTTCTGAGTCCATTTAGTTTGTGAGGATGTTCTAT
GTTCAGAAATAGTGGTGATGCCCAATTGGACTTAAAAGTTTTACACACTATGTGCATTTTACTTTCAGTTTAAGGAGTTT
TGCAACTGTGCATTGCAGCAGCCTTTATCGGTCTTGATTAAACTATGATTCCAAAATGTTTTTTTGCCAGTGTTCTGAAA
CTGTTTAAACCTGTAGTAGTTGCTATCAATTTATTTTCGGTAAGAAGAGTTGCTTAACTTGCTATTGACATCGTACCAAT
GACCTATATTAAGAATTTAAGACTGGCCTTGGTTGCTCATTAGGAATAAACTAAGGCCAGTAAGAAAGAGGGAATTTGTT
TAGATATCCTTATTACGAGTAGAAGGACAAGTCTGGTGCAGTGGTGAGACCTGCCTCACTGAGTCGCCAGGTTGTGGTTC
GAAGCAGCCTCTTGCATTTGCAGGGAAAGTCTTGCTTTGGTTGATCTCTTCTGCAGACCCCACTCACGTGGGAGCCTTCG
GCACTGGGTCTGCCTTTTTTATTTAGTGATCTGCATATGTACACTGCACTCAGCAGAAGAAAGTACACTTCTGTCTTGTA
GTAACATAACATTTGTAGGTTTTGGTTAGTCTTCACCCACGCATACATGATCAACCAGCAGCCTGGTTTCTTCTTTAGGT
TGATGTCCCAACAGCTGCTGTTTCTGCTTTATCCTTATTGAGACATGACGATTCATTCATCAGAAGGCCAACTTGGGATG
TAGGCTGGTCATTGGCTGATGCTAATCAGAAACAGGTCTCTTTGTTCATCGAATCAAAGTATTAAACTGTTGTCCCTCTT
TTGTCTTGCATTAGAACTTACCGTTACAATTTACAAGGTTGAAAATGACAGCAGATCTTCCCTCGAGTCTAACAGGAATA
ATTCATCTTTGGAGTCAGTAGACTCATTGATGTTGGCCAAGTCTGCCACAAGAATTGCTATTCTAGGAATTTCAACCTCC
AATTTAAATGTAAGGCACATGTCGATATATTTTTCTTTTCCTGAAAATTAGCACTTTGAAAGTTACAAGCTGTAGTTTTT
TTGTACTGAGTATTCTTTGGTCGTTGGTGAACAGGATGCAAGACGTATCAATGTTTCAGTGATGCAACAACGAGGGGACC
CTTTGCTTATAGTAGGATCTCCATTTGACCTCATGTCACCCTTCCATTTCTTCAACAGGTAATGTTTATTCTACTGCTTT
AGCAGAATGCCAGCATGTCTTTAACTCTTTAAGTATGCACCAGAATTTAGTGCATGTAACCTGTCGAACATTTTGATTTG
ATGATGACAAGAACATAGTCTGGGCATTCATAATGTTGACTTATGCAATGGTAAACTCCAGATGTTTTAAAACTTGATAC
TGTTCCATATGCATTATGGAGAAAACACTAAACTTTTAATATCTAGAGTTTTATTAACTCTTTGCTTTGCACGTCATCAT
TACATCTTCCAAAGCATATACATTTGTTGTGTTGTGTTTGTTCCCGGTATGTCTAGTGGCCTGGAAAGCGTTAACTAGAT
ACTGATGTTTCTAAAATTGAAAACGCCTGGCATGCTTTTGTTGACTATTTTCTTGTGCTACCATTATTAGAAAACAGAAT
AAGAGTGTCATATTTATTTGTATACATATGTGTTTTTTGAGCAGTGTATCAGTTGGTGCTGTTGCAAATTGCCTTCCTCC
ATGCACTGCAAGGAGCTCATTACTGATGGCTGACATGCACTGTCTCCCTGGTAAACATCTTTCTGTCTTTATTTTCTTGT

ATGGTATGACATTAGTTATTGATAATCTATTAAATTGACCAAAGGCATGGAAGGCGCTCCAGTGTTTGACCAAAATTCTT
GCCTCGTGGGGCTGCTGATGAACCCATTAACACAGAAAGGCAGCAATATAGAAGTCCAGGTTAGTTCACTGGTTTCAACA
AGATACATTTGCACATCGAATATTTGTAACTTAATTATCTTAAAATCTACTTACCAGCTCGTGATTACATGGGATGCAAT
ATGCACGGAATGGAACAGCAAAAAACTGGAGGAAATTGAACGACCCCCAAGAAAACTACCTAATGACAAAAATACAGATA
GTAAATCTATGGAATTACGGCATGTATATAACTATGTGAGGGTTTTCTCTTCTACGGACAACAAAACTAATCAGCATTGC
ATATCACCCCGTTCGCTCAGAGAGGCTATATCTGCAGTTGTTCTTGTCACGGTTGGTGATACGTCTTGGGCTTCAGGTAT
TGTTCTGAACAAAAGGGGTTTAGTTCTGACAAATGCTCATCTCTTGGAACCTTGGAGATTTGGAAGAACTTCACCTTCAG
ATTTACAAGCCTCGTTCGCTGGAGAACATCTCAATGCTGGAGAAAACAAATCATTGCAACCACAACAAGGCAAAATTTCC
AATGAAGATGCTGTCAAGCATAAGGTTTCGTCATTTAACTTGGGTTTCAAAAGAGGGAAGAGAATATCTGTTCGTTTGGA
CCATGAAGAGAGACAGATATGGTGCAATGCTAGTGTGGTTTTCATCTCAAAGGGTCCACTTGATGTTGCATTGCTTCAAA
TAGAAAAGGTTCCAGTTGAATTAAATACAATCAGACCAGAATTTGTTTGTCCAACAGCAGGGTCGCCTGTTTATGTTGTT
GGGCATGGCCTTTTTGGACCCCGATCAGGTGAAAAATCTTAGAACCGCAGCTTAAATTAGTGTTTGAGCAGTTCGATGTT
TTTTTATCAATGTGCATGGCTGCATGCCATTTCAAATGCTGAGTTTTGTTATTATGGAACTGATGTTTGAGTATCTGAAT
TTAGGCCTACACTCTTCTCTATACTCGGGGGTTGTGTCAAAGGTTGTCCAAATCCCAGCAAATCAACTTTCTCATCTGGC
CCGTGCTGAGGCTGACAATATGGACATACCAGTAATGCTTCAGACAACAGCAGCAGTTCATCCAGGAGCCAGTGGCGGCG
TTCTTGTTAATACACATGGGCTAATGGTTGGGATAATAACAAGGTATAAAATAGAACCTTTTTGAAGCCTTACATACGTT
AACTAGAACCTAAAGTTCATCAAGTTATTTTGCCATTTCATCGACTGTATCAACTTTCTACATTTTATGTTGTTATGTGT
CATTTTATGATATGCTAAAACATTGCAAGCATAGCATCATGTCATTCATTTTCGTAACTAGAATAATGGTGAAGTATGTA
TTTTCCAAAGAGCTTGTCTTTTTTCTGCCTTATTTTTTTTATTACCATATCCAGTAATGCTAAGCATGGTGGTGGAAGCA
CAATACCTCATCTGAATTTCAGCATCCCCTGCAAATTACTGGTTGCAGTCTTCGAGTATTCAGGTGCTAATTAAGTTTGC
TCGTATTTTTTCTGCTCTCAGCTTTAGTCAATATTGATGGTATCAAAACCCTTCTAAGTACATATAAAAATAAGGAATGC
ATTTCCTTTCTTGGCAGCAAATGGAAACCTCGTGGTTTTGGAGCAGTTGGACAAACCAAATGAAGTGCTCTCATCAGTTT
GGGCATTGGCACCATCATCATCCCCATTTGTCAGGAGCTCCCCAGAAAAAGGCAAAGAGGAAAAGGTCTTGGAGTTCTCT
AAGTTTCTTAGTGACAAGCAACAAGCTCTGAAATCTAACGTAGATCTGAAGGAACTATTTAGGTACAAGACTCCCAGCAA
AATATAGAGTGCGTCCACTAGAATTCATACAAGGAAAATGTTTGTTCCCAGAAATTTGTATGAGACGTTTGATCCCAGAA
ATCCCCAGTACAAAATGAGTTATCAGAGCACAGTGCCTTTTTTTTACAGATGGTTAAGGCATAAGCATCTTTTCTTCGAG
TGTCAGGTAGCGCGGGCAATATGGCATACTGTTCAAATCGCTACAATTTTTACCCTCCCTCTAGTGTCGTGAATATGTTT
AGGTCATAGCTAGGGGGATTGGTAAAGATTTAAAATTGTTAGCTCTTTTAGGGACAGCAGCAGTTTGTTGGGCCATTTGA
CATTGCGTTTCAAAGAAAATATGTGACAAACTCTTTACAGGTTCTTCGTTTGGTTATTCACTGGCTCCGTTCCTAGGTTG
TGCTACAGAAGCCTATCTTTCAGGAGTTGGTTTTGGCTACCTGACAACGTTTGGAGCAAATGGTCAGGTTGTTTTTTACC
TAGGCTCGCGGGTGGCAATCTAGTCTTAGGATTGATTTCTACTAGAGTTATTAGGTTGTTAGTCTTCTTTAGACTGTGTG
CATCTAGCGCGATGTAGAAGTTTTGTTGTCAAGAAAGTTGTGACTTATGGTTATCTTGATGCAATAAATTTTCTCTTCTC
GAAAAAAATGATGATGCCGCTGGGAATGTAATAAACTTATATAGGAACAGTTTTTACAATTGAAAATGTCAATTACC

>KS23-6_Peptidase_mRNA
ATCCAAACAGCTTTCAAGGAGAAGCGTACAGTCCCTGCTATACAGCTCTCGTCGGGGTCTTCTCCCCCTTCGGCCCTTCC
CCCCTCCCTGCGCGCCATGGAGGCCCAGGAGATCGCCGCTGCAGCGCGCCACTTCTGCGCCATGGTTCGAATCGTCGGTC
CGGACCCCAAGGCCGTGAAGATGCGCCGCCACGCCTTTCATTTTCACCATTCGGGGTCGACCACGCTCTCGGCGTCGGCT
CTGCTCCTGCCGCGGGGCGCTTTAGCTGAGCCGCCGCCATTTCTCGACCATATCTGCTCGGCCCACGGGCACACGGCAGG
AGACGTCGCGCTCACGGCTGCTTCCCTCGTCGAGCCGTTCCTGGTCGCGGAGCAGCGCAATAACTCCGGCGAGGAGCTTC
AGCCGAGGTTGGTTCCGGAGACGCGTCTTGATGTGTTTGTTGAGTATGAGTTGGGGAATGCTCAAGATGGGAAGTCTGGA
CCTCCGCGGTGGCTTCCAGCTCGACTGCTTGCCATGGTTGATGTCCCAACAGCTGCTGTTTCTGCTTTATCCTTATTGAG
ACATGACGATTCATTCATCAGAAGGCCAACTTGGGATGTAGGCTGGTCATTGGCTGATGCTAATCAGAAACAGGTCTCTT
TGTTCATCGAATCAAAATCTTCCCTCGAGTCTAACAGGAATAATTCATCTTTGGAGTCAGTAGACTCATTGATGTTGGCC
AAGTCTGCCACAAGAATTGCTATTCTAGGAATTTCAACCTCCAATTTAAATGATGCAAGACGTATCAATGTTTCAGTGAT
GCAACAACGAGGGGACCCTTTGCTTATAGTAGGATCTCCATTTGACCTCATGTCACCCTTCCATTTCTTCAACAGTGTAT
CAGTTGGTGCTGTTGCAAATTGCCTTCCTCCATGCACTGCAAGGAGCTCATTACTGATGGCTGACATGCACTGTCTCCCT
GGCATGGAAGGCGCTCCAGTGTTTGACCAAAATTCTTGCCTCGTGGGGCTGCTGATGAACCCATTAACACAGAAAGGCAG
CAATATAGAAGTCCAGCTCGTGATTACATGGGATGCAATATGCACGGAATGGAACAGCAAAAAACTGGAGGAAATTGAAC
GACCCCCAAGAAAACTACCTAATGACAAAAATACAGATAGTAAATCTATGGAATTACGGCATGTATATAACTATGTGAGG
GTTTTCTCTTCTACGGACAACAAAACTAATCAGCATTGCATATCACCCCGTTCGCTCAGAGAGGCTATATCTGCAGTTGT
TCTTGTCACGGTTGGTGATACGTCTTGGGCTTCAGGTATTGTTCTGAACAAAAGGGGTTTAGTTCTGACAAATGCTCATC
TCTTGGAACCTTGGAGATTTGGAAGAACTTCACCTTCAGATTTACAAGCCTCGTTCGCTGGAGAACATCTCAATGCTGGA
GAAAACAAATCATTGCAACCACAACAAGGCAAAATTTCCAATGAAGATGCTGTCAAGCATAAGGTTTCGTCATTTAACTT
GGGTTTCAAAAGAGGGAAGAGAATATCTGTTCGTTTGGACCATGAAGAGAGACAGATATGGTGCAATGCTAGTGTGGTTT
TCATCTCAAAGGGTCCACTTGATGTTGCATTGCTTCAAATAGAAAAGGTTCCAGTTGAATTAAATACAATCAGACCAGAA
TTTGTTTGTCCAACAGCAGGGTCGCCTGTTTATGTTGTTGGGCATGGCCTTTTTGGACCCCGATCAGGCCTACACTCTTC

TCTATACTCGGGGGTTGTGTCAAAGGTTGTCCAAATCCCAGCAAATCAACTTTCTCATCTGGCCCGTGCTGAGGCTGACA
ATATGGACATACCAGTAATGCTTCAGACAACAGCAGCAGTTCATCCAGGAGCCAGTGGCGGCGTTCTTGTTAATACACAT
GGGCTAATGGTTGGGATAATAACAAGTAATGCTAAGCATGGTGGTGGAAGCACAATACCTCATCTGAATTTCAGCATCCC
CTGCAAATTACTGGTTGCAGTCTTCGAGTATTCAGCAAATGGAAACCTCGTGGTTTTGGAGCAGTTGGACAAACCAAATG
AAGTGCTCTCATCAGTTTGGGCATTGGCACCATCATCATCCCCATTTGTCAGGAGCTCCCCAGAAAAAGGCAAAGAGGAA
AAGGTCTTGGAGTTCTCTAAGTTTCTTAGTGACAAGCAACAAGCTCTGAAATCTAACGTAGATCTGAAGGAACTATTTAG
GTACAAGACTCCCAGCAAAATATAGAGTGCGTCCACTAGAATTCATACAAGGAAAATGTTTGTTCCCAGAAATTTGTATG
AGACGTTTGATCCCAGAAATCCCCAGTACAAAATGAGTTATCAGAGCACAGTGCCTTTTTTTTACAGATGGTTAAGGCAT
AAGCATCTTTTCTTCGAGTGTCAGGTAGCGCGGGCAATATGGCATACTGTTCAAATCGCTACAATTTTTACCCTCCCTCT
AGTGTCGTGAATATGTTTAGGTCATAGCTAGGGGGATTGGTAAAGATTTAAAATTGTTAGCTCTTTTAGGGACAGCAGCA
GTTTGTTGGGCCATTTGACATTGCGTTTCAAAGAAAATATGTGACAAACTCTTTACAGGTTCTTCGTTTGGTTATTCACT
GGCTCCGTTCCTAGGTTGTGCTACAGAAGCCTATCTTTCAGGAGTTGGTTTTGGCTACCTGACAACGTTTGGAGCAAATG
GTCAGGTTGTTTTTTACCTAGGCTCGCGGGTGGCAATCTAGTCTTAGGATTGATTTCTACTAGAGTTATTAGGTTGTTAG
TCTTCTTTAGACTGTGTGCATCTAGCGCGATGTAGAAGTTTTGTTGTCAAGAAAGTTGTGACTTATGGTTATCTTGATGC
AATAAATTTTCTCTTCTCGAAAAAAATGATGATGCCGCTGGGAATGTAATAAACTTATATAGGAACAGTTTTTACAATTG
AAAATGTCAATTACC

>KS23-6_Peptidase_Protein
MEAQEIAAAARHFCAMVRIVGPDPKAVKMRRHAFHFHHSGSTTLSASALLLPRGALAEPPPFLDHICSAHGHTAGDVALT
AASLVEPFLVAEQRNNSGEELQPRLVPETRLDVFVEYELGNAQDGKSGPPRWLPARLLAMVDVPTAAVSALSLLRHDDSF
IRRPTWDVGWSLADANQKQVSLFIESKSSLESNRNNSSLESVDSLMLAKSATRIAILGISTSNLNDARRINVSVMQQRGD
PLLIVGSPFDLMSPFHFFNSVSVGAVANCLPPCTARSSLLMADMHCLPGMEGAPVFDQNSCLVGLLMNPLTQKGSNIEVQ
LVITWDAICTEWNSKKLEEIERPPRKLPNDKNTDSKSMELRHVYNYVRVFSSTDNKTNQHCISPRSLREAISAVVLVTVG
DTSWASGIVLNKRGLVLTNAHLLEPWRFGRTSPSDLQASFAGEHLNAGENKSLQPQQGKISNEDAVKHKVSSFNLGFKRG
KRISVRLDHEERQIWCNASVVFISKGPLDVALLQIEKVPVELNTIRPEFVCPTAGSPVYVVGHGLFGPRSGLHSSLYSGV
VSKVVQIPANQLSHLARAEADNMDIPVMLQTTAAVHPGASGGVLVNTHGLMVGIITSNAKHGGGSTIPHLNFSIPCKLLV
AVFEYSANGNLVVLEQLDKPNEVLSSVWALAPSSSPFVRSSPEKGKEEKVLEFSKFLSDKQQALKSNVDLKELFRYKTPS
KI
